# Surface Water Microbial Community Response to the Biocide 2,2-Dibromo-3-Nitrilopropionamide, Used in Unconventional Oil and Gas Extraction

**DOI:** 10.1128/AEM.01336-19

**Published:** 2019-10-16

**Authors:** Maria Fernanda Campa, Stephen M. Techtmann, Mallory P. Ladd, Jun Yan, Megan Patterson, Amanda Garcia de Matos Amaral, Kimberly E. Carter, Nikea Ulrich, Christopher J. Grant, Robert L. Hettich, Regina Lamendella, Terry C. Hazen

**Affiliations:** a Bredesen Center for Interdisciplinary Research and Graduate Education, University of Tennessee, Knoxville, Tennessee, USA; b Biosciences Division, Oak Ridge National Laboratory, Oak Ridge, Tennessee, USA; c Department of Biological Sciences, Michigan Technological University, Houghton, Michigan, USA; d Chemical Sciences Division, Oak Ridge National Laboratory, Oak Ridge, Tennessee, USA; e Key Laboratory of Pollution Ecology and Environmental Engineering, Institute of Applied Ecology, Chinese Academy of Sciences, Shenyang, Liaoning, People’s Republic of China; f Department of Microbiology, University of Tennessee, Knoxville, Tennessee, USA; g Department of Civil and Environmental Engineering, University of Tennessee, Knoxville, Tennessee, USA; h Department of Biology, Juniata College, Huntingdon, Pennsylvania, USA; i Department of Earth and Planetary Sciences, University of Tennessee, Knoxville, Tennessee, USA; j Institute for a Secure and Sustainable Environment, Knoxville, Tennessee, USA; University of California, Davis

**Keywords:** 16S rRNA, DBNPA, hydraulic fracturing, microbial communities, microbial ecology, unconventional oil and gas, water contamination

## Abstract

Production of unconventional oil and gas continues to rise, but the effects of high-density hydraulic fracturing (HF) activity near aquatic ecosystems are not fully understood. A commonly used biocide in HF, 2,2-dibromo-3-nitrilopropionamide (DBNPA), was studied in microcosms of HF-impacted (HF+) versus HF-unimpacted (HF−) surface water streams to (i) compare the microbial community response, (ii) investigate DBNPA degradation products based on past HF exposure, and (iii) compare the microbial community response differences and similarities between the HF biocides DBNPA and glutaraldehyde. The microbial community responded to DBNPA differently in HF-impacted versus HF-unimpacted microcosms in terms of the number of 16S rRNA gene copies quantified, alpha and beta diversity, and differential abundance analyses of microbial community composition through time. The differences in microbial community changes affected degradation dynamics. HF-impacted microbial communities were more sensitive to DBNPA, causing the biocide and by-products of the degradation to persist for longer than in HF-unimpacted microcosms. A total of 17 DBNPA by-products were detected, many of them not widely known as DBNPA by-products. Many of the brominated by-products detected that are believed to be uncharacterized may pose environmental and health impacts. Similar taxa were able to tolerate glutaraldehyde and DBNPA; however, DBNPA was not as effective for microbial control, as indicated by a smaller overall decrease of 16S rRNA gene copies/ml after exposure to the biocide, and a more diverse set of taxa was able to tolerate it. These findings suggest that past HF activity in streams can affect the microbial community response to environmental perturbation such as that caused by the biocide DBNPA.

**IMPORTANCE** Unconventional oil and gas activity can affect pH, total organic carbon, and microbial communities in surface water, altering their ability to respond to new environmental and/or anthropogenic perturbations. These findings demonstrate that 2,2-dibromo-3-nitrilopropionamide (DBNPA), a common hydraulic fracturing (HF) biocide, affects microbial communities differently as a consequence of past HF exposure, persisting longer in HF-impacted (HF+) waters. These findings also demonstrate that DBNPA has low efficacy in environmental microbial communities regardless of HF impact. These findings are of interest, as understanding microbial responses is key for formulating remediation strategies in unconventional oil and gas (UOG)-impacted environments. Moreover, some DBNPA degradation by-products are even more toxic and recalcitrant than DBNPA itself, and this work identifies novel brominated degradation by-products formed.

## Abstract (precis)

Unconventional oil and gas activity can affect pH, total organic carbon, and microbial communities in surface water, altering their ability to respond to new environmental and/or anthropogenic perturbations. These findings demonstrate that 2,2-dibromo-3-nitrilopropionamide (DBNPA), a common hydraulic fracturing (HF) biocide, affects microbial communities differently as a consequence of past HF exposure, persisting longer in HF-impacted (HF+) waters. These findings also demonstrate that DBNPA has low efficacy in environmental microbial communities regardless of HF impact. These findings are of interest, as understanding microbial responses is key for formulating remediation strategies in unconventional oil and gas (UOG)-impacted environments. Moreover, some DBNPA degradation by-products are even more toxic and recalcitrant than DBNPA itself, and this work identifies novel brominated degradation by-products formed.

## INTRODUCTION

Unconventional oil and gas (UOG) extraction has revolutionized the energy industry in the United States. The use of hydraulic fracturing (HF) has made previously unreachable UOG reserves available for economically feasible extraction and pushed the United States toward energy independence (1). Multiple environmental concerns have accompanied this energy production growth. Among the most commonly added chemicals to HF fluids are biocides. Biocides are used in HF operations to control microbially induced corrosion of casings and pipes and gas souring caused by acid-producing and sulfate-reducing bacteria (2). However, biocides have warranted concern for several reasons. Biocides have various degrees of reported efficacy due to potential resistance or inactivation of the biocides in HF conditions (2–5). Additionally, their toxicity and potential impact on the environment remain a contentious topic (2, 6). The fate of these biocides in the environment and their impact on microbial communities are poorly understood.

The biocide 2,2-dibromo-3-nitrilopropionamide (DBNPA) is the second most commonly used biocide in UOG after glutaraldehyde. DBNPA is a fast-acting electrophilic biocide; it is quick and effective in contact, but the protection is not long lasting (7). This biocide inhibits essential biological functions by reacting with nucleophiles (particularly sulfur-containing nucleophiles) inside the cell (8). DBNPA, and some of its degradation products, can also be harmful to humans and animals. These associated compounds have been demonstrated to be moderately to highly toxic by ingestion and inhalation, can be corrosive to eyes, and have been shown in terrestrial and aquatic animal studies to cause developmental issues (9, 10).

DBNPA is not toxic to all life, however, as it is biodegradable under both aerobic and anaerobic conditions, with a reported biotic half-life of less than 4 h under both conditions at neutral pH (10). However, the hydrolysis and aquatic photolysis half-life of this compound are pH-dependent, with faster degradation occurring at a more alkaline pH. For example, the abiotic half-lives of DBNPA at pH 5, 7, and 9 are 67 days, 63 h, and 73 min, respectively (10). Conversely, low pH has been characteristic of HF-impacted streams (11, 12), which thus provide favorable conditions for the stability of DBNPA and its degradation products.

The products of DBNPA biodegradation are the same under aerobic and anaerobic metabolism (10). Still, the relative abundance of these degradation intermediates and their reported half-lives vary depending on conditions such as pH, hydrolysis, photolysis, nucleophile presence, and aerobic or anaerobic conditions (10, 13). There are two known degradation pathways of DBNPA (Fig. S1). The first pathway involves the hydrolysis of DBNPA into dibromoacetonitrile (DBAN), then dibromoacetamide (DBAM), and finally dibromoacetic acid. DBAN is more recalcitrant and three times more toxic than DBNPA (13). Dibromoacetic acid, a problematic disinfection by-product (14), has a half-life of 300 days and breaks down into glyoxylic acid, oxalic acid, bromide ions, and carbon dioxide (15). However, a higher presence of total organic carbon (TOC) and/or nucleophilic reactions under UV light favors a second degradation pathway, where DBNPA degrades to monobromonitrilopropionamide (MBNPA), a compound two times less toxic than DBNPA (13), and then to cyanoacetamide (CAM) (13, 15). It was previously shown that HF-impacted streams have larger amounts of dissolved organic carbon than HF-unimpacted streams (16), which may impact DBNPA degradation products in impacted environments.

DBNPA can reach the environment in many ways, including surface spills into the soil, surface water, and aquifers; incomplete removal after water treatment; groundwater contamination after equipment failure (leakage); and unintended fractures or abandoned wells (2). DBNPA environmental contamination could also occur in several of the steps associated with HF operations, e.g., the transportation of chemicals to the site; mixing of HF fluids and chemicals on site; subsurface injection of the HF fluids; handling, collection, and storage of produce water; and disposal of the produced water (17). Understanding the impacts of surface and shallow groundwater spills, leaks, and disposal of poorly treated HF wastewater in the environment is of great concern, as several studies have reported cases of the accumulation of toxic chemicals (such as hydrocarbons, benzene, toluene, ethylbenzene, and xylene, diesel, and chlorinated solvents, among others) in groundwater, streams, soils, and sediments at HF operating sites (18–22). However, no studies have investigated DBNPA degradation by-products and the microbial community changes over time in aerobic stream waters impacted by HF. This study aims to (i) understand the differences in local stream microbial community responses to DBNPA, (ii) identify DBNPA degradation by-products in streams impacted and unimpacted by HF operations, and (iii) compare the microbial community response differences and similarities between the HF biocides DBNPA and glutaraldehyde.

## RESULTS AND DISCUSSION

### Quantification of bacterial 16S rRNA gene abundance over time.

**FIG 1:**
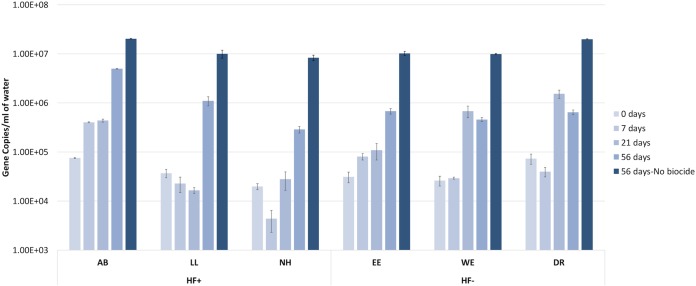
Impacts of DBNPA on abundance of 16S rRNA gene copies/ml over time. Data shown are divided by HF-impacted (first three clusters: Alex Branch [AB], Little Laurel [LL], and Naval Hollow [NH]) and HF-unimpacted (East Elk [EE], West Elk [WE], and Dixon Run [DR]) microcosms at day 0 before DBNPA addition, days 7, 21, and 56 after DBNPA addition, and day 56 for the no-DBNPA-added control. The bars are colored on a gradient over time, with the last bar representing the no-DBNPA control at day 56. Each bar represents *n* = 3, and the error bars represent one standard error.

The 16S rRNA gene abundance was quantified at various points through the course of the experiment (Fig. 1). Prior to DBNPA addition, the starting mean 16S rRNA gene concentrations were 4.03 ± 0.60 × 10^4^ gene copies/ml in the HF-impacted (HF+) stream microcosms and 4.38 ± 0.50 × 10^4^ gene copies/ml in HF-unimpacted (HF−) stream microcosms. This difference was not statistically significant. Bacterial 16S rRNA gene copies in microcosms from two HF+ streams (Little Laurel [LL] and Naval Hollow [NH]) decreased immediately following addition of DBNPA and then increased, while the Alex Branch (AB) microcosm displayed an increase in bacterial 16S rRNA gene concentration by day 7. In contrast, 16S rRNA gene abundance in microcosms from two HF− streams (East Elk [EE] and West Elk [WE]) increased, while the Dixon Run (DR) microcosm experienced a decrease in 16S rRNA gene concentration by day 7. Specifically, 7 days after addition of DBNPA, an average decrease of –0.16 log_2_ fold change (FC) in 16S rRNA gene copies/ml was observed in HF+ microcosms, and a small average increase of 0.22 log_2_ FC was observed in HF− microcosms, indicating more sensitivity to DBNPA in HF+ microcosms. However, by day 56, the HF+ microcosms experienced a 4.9 log_2_ FC and HF− microcosms experienced a 3.9 log_2_ FC. The difference in average number of 16S rRNA gene copies/ml through time (day 7 to 56) between HF+ and HF− microcosms was statistically significant (*P* < 0.05). At day 56, the HF+ and HF− controls (microcosms with no DBNPA added) were not significantly different from each other; both experienced an 8.3 log_2_ FC from the number of initial gene copies/ml at day 0. The similitude in starting microbial abundance prior to DBNPA addition indicates that the difference in microbial abundance observed after DBNPA addition was due to the initial impact of DBNPA on the microbial community, followed by its response and adaptation to the biocide and low biocidal activity of DBNPA over time.

Quantification of the 16S rRNA gene shows that the HF− microbial communities were initially more resistant and/or tolerant to DBNPA perturbation, as shown by the overall positive log fold change in the gene copy number at day 7. DBNPA is a fast-kill biocide, and thus resistance at the initial time point is indicative of inefficacy of microbial control in HF− microcosms. Through time, both HF− and HF+ microcosms showed strong resilience and adaptation to decreasing concentration of DBNPA; however, by day 56, HF+ microcosms had an overall greater number of gene copies/ml (Fig. 1) and higher log fold change than HF− microcosms.

There is no indication that the HF+ streams had any prior exposure to DBNPA prior to this experiment. UOG operators in the area have disclosed the use of other biocides, such as glutaraldehyde (reported via the self-disclosure website fracfocus.org). Thus, prior exposure to HF activity not involving DBNPA did not appear to provide “priming” or a competitive advantage to DBNPA exposure based on 16S rRNA gene copies/ml alone, but it provided favorable conditions for quicker resilience (23). Furthermore, quantification of the 16S rRNA gene copy number shows that there is overall environmental tolerance to high concentration of DBNPA, indicating that DBNPA is not as effective in controlling complex and dynamic microbial communities as in controlling environmental isolates or engineered systems (9, 24).

### Microbial community structural changes.

**FIG 2:**
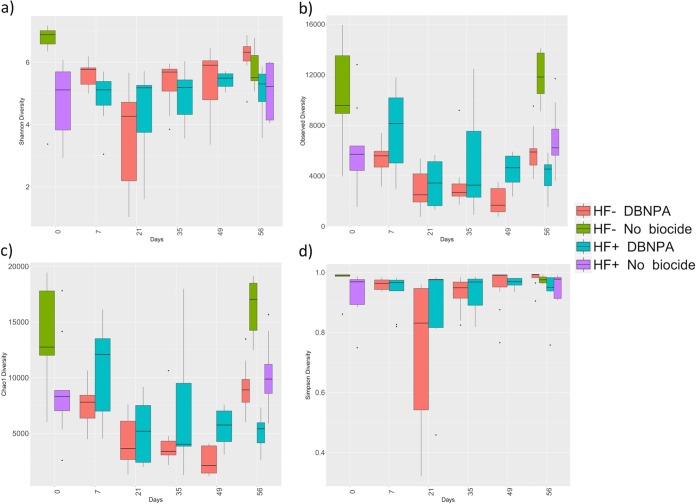
Four different richness and evenness alpha diversity estimators comparing HF-impacted and HF-unimpacted microcosms over time. The estimators used were (a) Shannon diversity, (b) observed diversity, (c) Chao1, and (d) Simpson diversity. Red and green represent HF-unimpacted microcosms. Red boxes represent the changes after DBNPA addition in HF-unimpacted microcosms (days 7 to 56), while the green boxes represent the alpha diversity without DBNPA addition in HF-unimpacted microcosms (days 0 and 56). Blue and purple boxes represent HF-impacted microcosms. Blue boxes represent the changes after DBNPA addition in HF-impacted microcosms (days 7 to 56), while the purple boxes represent the alpha diversity without DBNPA addition in HF-impacted microcosms (days 0 and 56). The box-and-whisker plot describes the distribution of the data points. The beginning of the whiskers to the beginning of the box covers the upper and lower quartiles. The box represents the interquartile range, which represents 50% of the data points (*n* = 9). The vertical line inside the box represents the median.

Microorganisms in headwater ecosystems are environmental regulators of natural geochemical cycles and organic matter cycling (25, 26). Microorganisms are very sensitive to perturbation, which makes them good sensors of environmental change and effective for tracking contaminants (27). Before DBNPA addition, HF− microcosms had an overall higher evenness and richness than HF+ microcosms. After addition of DBNPA, evenness and richness were affected through time in both HF+ and HF− microcosms. Shannon diversity measurements, which account for the abundance and evenness of species present, showed that HF+ microcosms experienced a smaller decrease in evenness and richness—even though HF− microcosms had an overall higher diversity (Fig. 2a) (*P* < 0.01). Meanwhile, while not statistically significant, Simpson diversity measurements (Fig. 2d), which also account for the abundance of species present, indicated minimal changes in diversity over time except for that in HF− microcosms at day 21. Still, diversity increased by day 35. In contrast, Chao1 (*P* < 0.05) and observed (*P* < 0.05) measurements (Fig. 2b and c), which include unique and rare operational taxonomic units (OTUs) in their calculations, experienced a more prominent decrease in diversity, as fewer OTUs dominated over time. In contrast, when comparing day 0 with day 56 controls to test the bottle effect, the changes detected by day were not significant, and HF− microcosms maintained higher diversity than that of HF+ microcosms.

**FIG 3:**
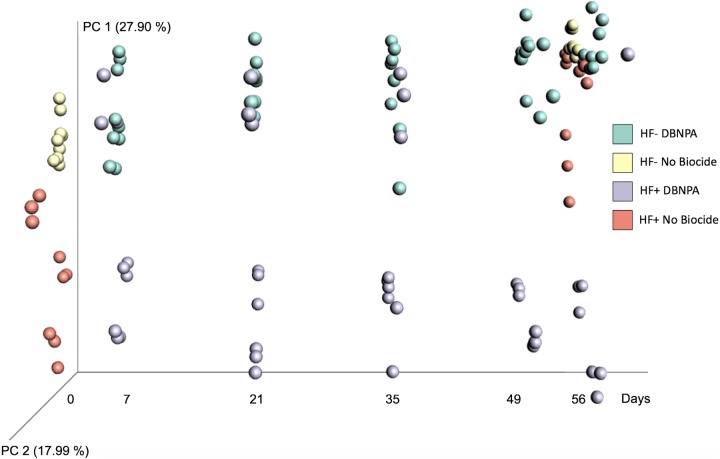
Directional principal coordinate analysis (PCoA) plots of weighted UniFrac distances between microcosms. Samples were plotted on the *x* axis from left to right according to days sampled (0, 7, 21, 35, 49, and 56). Samples are colored by hydraulic fracturing (HF) impact history and DBNPA addition. Green, HF-unimpacted plus DBNPA addition; yellow, HF-unimpacted without biocide addition; purple, HF-impacted plus DBNPA addition; pink, HF-impacted without biocide addition. Samples without biocide addition were measured only on days 0 and 56.

Analysis of weighted UniFrac distances between samples revealed that there was a difference in phylogenetic composition response between HF+ and HF− microbial populations. The weighted UniFrac distances were plotted on a directional principal coordinate analysis (PCoA). Principal coordinate 1 (PC1) explained 27.90% of the sample variance, while PC2 explained 17.99% of the sample variance (Fig. 3). At day 0, prior to DBNPA addition, HF+ and HF− microcosms already clustered separately along the PC1 axis, but after DBNPA addition, HF+ and HF− visibly separated more over time, showing that the HF+ and HF− microcosms became more dissimilar over time after addition of DBNPA. Meanwhile the HF+ and HF− no-DBNPA-added controls clustered together at day 56. Permutational multivariate analysis of variance (PERMANOVA) indicated that there were statistically significant differences between HF+ and HF− microbial communities through time (Table 1). This difference indicates that DBNPA selected for different sets of taxa based on HF exposure.

**TABLE 1:** Nested PERMANOVA^*a*^ of weighted UniFrac distances

Source of variation	No. of degrees of freedom	Sum of squares	Mean square	*F* statistic value	*R*^2^	*P* value
HF_ImpactStatus	1	0.9515	0.95148	30.3412	0.15771	0.001
Biocide	1	0.4506	0.45056	14.3678	0.07468	0.001
Biocide: Days	2	0.7840	0.39199	12.5000	0.12995	0.001
HF_ImpactStatus: Biocide	1	0.1381	0.13806	4.4024	0.02288	0.001
HF_ImpactStatus: Biocide: Days	2	0.1653	0.08266	2.6359	0.02740	0.001
Residuals	113	3.5436	0.03136		0.58737	
Total	120	6.0330			1.00000	

a PERMANOVA, permutational multivariate analysis of variance.

### Differentially enriched taxa over time and between HF+ and HF− microcosms.

**FIG 4:**
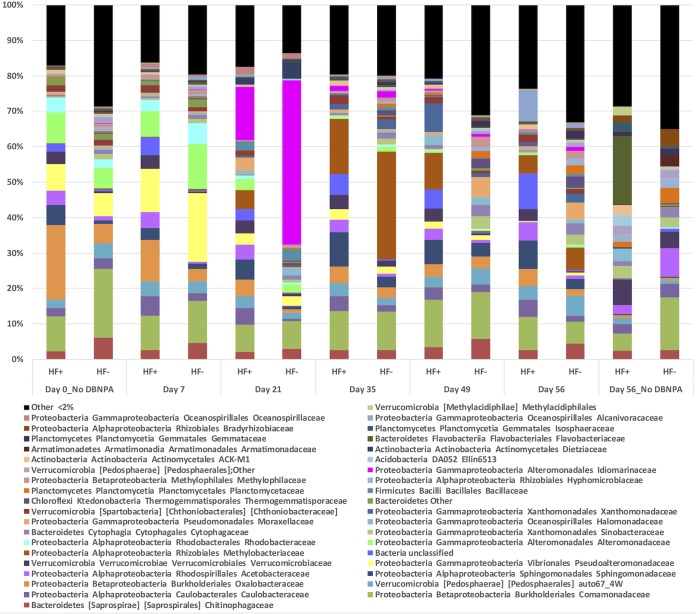
Temporal changes of microbial community relative abundance in averaged hydraulic fracturing-impacted (HF+) and hydraulic fracturing-unimpacted (HF−) microcosms treated with the biocide DBNPA. Microbial taxa are summarized to the family level.

The initial bacterial population (before DBNPA amendment) in all microcosms, regardless of HF history, was predominantly Proteobacteria, which comprised more than 75.5% ± 4.8% of 16S rRNA gene reads in the HF+ group and more than 64.4% ± 3.2% in the HF− group. *Proteobacteria* species were expected to dominate, as previous studies on these Pennsylvania streams reported this phylum as the principal population (11, 12, 16). However, initial proportions of Betaproteobacteria, Alphaproteobacteria, and Gammaproteobacteria, in that order of abundance, differed between the HF+ and HF− groups. Taxon plots illustrate the differences in microbial community structure over time (Figure S2a and S2b). *Gammaproteobacteria* were the first responders in both HF+ and HF− groups after 7 days of DBNPA addition, with Pseudoalteromonadaceae as the most dominant family at this time, comprising 12.3% ± 4.0% of HF+ microcosms and 19.4% ± 2.2% of HF− microcosms. A strong correlation between *Gammaproteobacteria* and HF+ streams has been shown before (11, 12). By day 35, *Alphaproteobacteria*, specifically, the genus Methylobacterium, was the most dominant taxon (15.6% ± 7.7% in HF+ and 30.5% ± 6.2% in HF− microcosms). However, by day 56, a more diverse microbial composition was observed, with few overall dominant taxa. In HF+ microcosms, the most dominant taxa were unclassified bacteria (10% ± 5.1%), Comamonadaceae (9.5% ± 2.7%), Alcanivoracaceae (8.9% ± 7.8%), and Sphingomonadaceae (7.9% ± 2.4%), and in HF− microcosms the most dominant taxa were *Comamonadaceae* (6.3% ± 0.8%), auto67_4w from the order Pedosphaerales (5.7% ± 1.3%), and Methylobacteriaceae (6.3% ± 2.7%) (Fig. 4).

There were important changes to the microbial community structure in both HF+ and HF− microcosms following amendment with DBNPA. Seven days after DBNPA amendment, the relative abundances of 29 taxa were significantly different (DESeq2, Wald test; *P* ≥ 0.01) in both HF+ and HF− microcosms compared to those at day 0; 24 of these taxa increased in relative abundance, and 5 decreased (Table S1). The two taxa with the greatest increases were identified as the family AEGEAN 185 (7.43 log_2_FC) from the SAR404 phylum and family SAR 324 (7.26 log_2_FC), a member of the class Deltaproteobacteria. Both of these taxa were reported in a similar experiment using the biocide glutaraldehyde (28). AEGEAN 185 matches to sequences of a clone library from the North Aegean Sea, but its metabolic profile is unknown (29). Sequenced members of SAR 324 are known to possess genes for methane monooxygenase and dehalogenases that, if expressed, can cometabolize halogenated compounds such as DBNPA (30–32). The only enriched genus with a relative abundance greater than 2% at all time points in the experiment was Alcanivorax (3.27 log_2_FC)*. Alcanivorax* is a known oil degrader (33) and was also enriched in glutaraldehyde microcosms, showing a wide range of xenobiotic compounds that it is capable of tolerating and even possibly degrading (28). In addition to the three previously mentioned taxa, 9 more were enriched with both glutaraldehyde and DBNPA, namely, Achromobacter, Synechococcus, SarSea-WGS and Artic95A-2 from the SAR 406 clade, Acidimicrobiales, Nitrospina, Sphingopyxis, and Euryarchaeota marine group II and III. Of the five suppressed OTUs, three were from the order Burkholderiales. Differential abundance analysis between HF+ and HF− microcosms at day 7 showed that 51 taxa had significantly different abundances; 30 were enriched in HF+ microcosms, and 21 were enriched in HF− microcosms (Table S2). The most substantial logFC was that of Micrococcus (6.14 log_2_FC), and the taxa that were enriched and comprised more than 2% relative abundance in HF+ microcosms were Verrucomicrobiaceae, Caulobacteraceae, Janthinobacterium, Novosphingobium, Oxalobacteraceae, and Limnohabitans.

The microbial communities at days 21, 35, 49, and 56 (Tables S3, S4, S5, and S6, respectively) followed a similar trend, with approximately 100 differentially enriched taxa in both HF+ and HF− microcosms compared to taxa at day 0. Through time, many OTUs related to marine environments, such as Idiomarina, SAR 324, Aegean-185, Alteromonadaceae, ZD017, Halomonas, and Alcanivorax, were enriched. Enrichment of marine taxa is notable, as osmotic regulation and efflux pumps, which are important attributes of marine microbes, have been linked to biocide tolerance (34–37), but mechanistic details about microbial tolerance to DBNPA have not been previously reported. Marine organisms are found in low abundance in freshwater streams, and they can bloom when conditions are favorable (38), which indicates a potential competitive advantage of halotolerant bacteria when exposed to DBNPA. For example, a halotolerant Halomonadaceae species was shown to be enriched in HF-exposed anaerobic sediments treated with DBNPA (39). Other halotolerant bacteria have also emerged as bacterial biomarkers of UOG impacts in freshwater streams (40). HF fluids contain high abundances of halophilic and halotolerant bacteria (41), which can be displaced to streams in the event of an HF fluid spill.

Other organisms that were differentially enriched between days 21, 35, 49, and 56 (Tables S3, S4, S5, and S6, respectively) included Dietzia, Bacillus, Methylobacterium, Verrucomicrobiaceae, Novosphingobium, and Caulobacteraceae, among others. *Dietzia* was previously shown to resist antimicrobials in freshwater and wastewater ecosystems (42). *Bacillus* has been reported to possess intrinsic resistance to antimicrobials, as it can form spores when antimicrobial pressure is encountered (43, 44). Bacterial spores are the least susceptible to biocidal action (43). *Methylobacterium* is a common environmental microbe that was previously shown to be enriched and dominant after a freshwater consortium was exposed to glutaraldehyde (28), and species in this genus have been shown to be resistant to other antimicrobials (45). Another microcosm study using an anaerobic mixture of sediment and water showed that DBNPA exposure decreased *Methylobacteriaceae* abundance (39), indicating that oxygen availability is needed for *Methylobacterium* resistance and enrichment in the presence of DBNPA. *Novosphingobium* species are commonly found in environments impacted by anthropogenic activity (46). They are known to be effective biodegraders of toxic and recalcitrant compounds (46). However, abundance of the family *Sphingomonadaceae*, of which *Novosphingobium* is a member, also was shown to decrease after exposure to DBNPA in a previous anaerobic sediment and water microcosm study (39), indicating again that oxygen or sediment presence affects tolerance and resistance of these taxa. Taxa enriched in HF+ but not HF− microcosms included *Verrumicrobiaceae* and *Caulobacteraceae*, which were shown by another study to be susceptible to a low dosage of DBNPA in sediments that were not exposed to HF (39). The enrichment of these taxa in HF+ microcosms only may indicate that prior exposure to HF fluids can build tolerance to DBNPA in *Verrumicrobiaceae* and *Caulobacteraceae* regardless of whether HF fluids contained DBNPA. Furthermore, *Caulobacteraceae* has been previously identified as a microbial biomarker of UOG activity in streams in Pennsylvania (40).

At day 56, the negative control had 209 differentially enriched taxa compared to taxa at day 0, which can be attributed to the bottle effect (Table S11). Meanwhile, at day 56, the experimental and negative-control microcosms (no DBNPA added) had 181 differentially enriched taxa. Of those, 111 were enriched in the experimental microcosms (Table S12), which, when summarized at genus level, reveals that Bacillus, Idiomarina, Glaciecola, Alcanivorax, Acinetobacter, Vibrio, Dietzia, Methylobacterium, Pseudoalteromonas, Marinobacter, Novosphingobium, Stenotrophomonas, Burkholderia, and Oxalobacteraceae (unclassified genus) show tolerance and adaptation to DBNPA.

Another study used 0.0025% (vol/vol) DBNPA with and without the addition of FeOOH in anaerobic microcosms constructed with sediment inoculum from upstream and downstream of a UOG wastewater treatment facility to understand how UOG wastewater processing affects downstream microbial communities and how those changes affect anaerobic microbial responses to HF fluid additives (39). That study found three families that were enriched in the UOG downstream microcosms amended with only DBNPA, and two of those, *Halomonadaceae* and Staphylococcaceae, were also found in this study. Conversely, the same UOG downstream samples were amended with FeOOH and DBNPA, and six families were enriched, two of which were also detected in this study, namely, Rhodospirillaceae (enriched over time in HF+ compared to HF− microcosms; Tables S2 and S7 to S10), and Ignavibacteriaceae (enriched at days 21and 35; Table S3 and S4). However, the study by Mumford et al. (39) only sampled at day 42 after incubation, and the low DBNPA concentration and sediment and anaerobic conditions used are expected to result in large differences between that study and the one described here.

### Microbial community responses to DBNPA versus glutaraldehyde.

We recently conducted a similar study using 100 mg/liter of the biocide glutaraldehyde (28). The changes in microbial abundance observed after treatment with DBNPA contrast with the results of the glutaraldehyde experiment. In the glutaraldehyde study, all of the six stream microcosms experienced an initial decrease in microbial abundance. On average, HF+ communities were initially more resistant to the biocide, as observed by a smaller log fold change of 16S rRNA gene copies/ml by day 7. By day 56, HF− communities showed stronger resilience by having a bigger positive log fold change. These results show that the microbial abundance adaptation response in these microbial communities is biocide specific.

*Methylobacterium*, *Idiomarina*, *Bacillus*, *and Alcanivorax*, among others, were enriched in the presence of both DBNPA and glutaraldehyde (28). These enrichments indicate that these taxa have a competitive advantage when exposed to these two electrophilic biocides. Previous studies have shown that glutaraldehyde resistance may be caused by the expression of efflux pumps (36, 47). However, the mechanisms for DBNPA resistance are not known, and functional genomics and transcriptomics analyses are needed to better understand these mechanisms.

Furthermore, weighted UniFrac beta diversity (Fig. 3) showed a distinct phylogenetic response between HF+ and HF− microcosms. This was similar to what was observed in previous work (28), yet glutaraldehyde showed more significant phylogenetic distances on a PCoA plot. The primary axis explained 65.4% of the variation and the secondary axis explained 10%, while for DBNPA, PC1 and PC2 explained 27.90% and 17.99%, respectively, showing that the response and phylogenetic changes due to DBNPA addition were not as pronounced as those for glutaraldehyde. Even though both are electrophilic biocides, DBNPA is a fast-kill biocide, while glutaraldehyde biocidal properties are longer lasting (2). Glutaraldehyde is also more persistent over time (28), with a biotic half-life of 33.8 days in HF− and a biotic half-life of 51.9 in HF+ microcosms, potentially explaining the more pronounced differences in the phylogenetic distribution of glutaraldehyde-treated microcosms over time. Furthermore, the alpha diversity changes and the differentially enriched taxa suggested that the microcosms contain a higher quantity of OTUs that are able to tolerate and adapt to DBNPA, rather than having just a few OTUs becoming enriched as in the case of glutaraldehyde (28). For example, *Methylobacterium* was the most dominant taxon by day 35 in the microcosms exposed to DBNPA (15.6% in HF+ and 30.5% in HF−), but by day 56, there is no clear dominating taxon. In contrast, when exposed to glutaraldehyde, *Methylobacterium* dominated the community from day 21 throughout day 56. At this final time point, *Methylobacterium* represented 70.6% of the observed microbial community in HF+ and 84.2% in HF− microcosms (28). Based on this comparison, combined with the significant increase in 16S rRNA gene copies, it seems that DBNPA tolerance is more ubiquitous. There are at least two possible explanations for this result, as follows: (i) changes in the microbial community structure and/or adaptation of individual community members improve resilience of the community as a whole, or (ii) DBNPA is degraded (either biotically or abiotically) more rapidly than glutaraldehyde (28, 48, 49).

### Abiotic and biotic transformation of DBNPA.

We evaluated the degradation of DBNPA over 56 days, using both biotic and abiotic microcosms constructed from HF+ and HF− streams (Fig. S3 and S4). However, the degradation rate of DBNPA could not be calculated, as quantification by high-performance liquid chromatography with diode-array detection (HPLC-DAD) revealed a sharp increase in DBNPA signal at day 14 at two of the HF+ sites with documented spills (AB and LL, both biotic and abiotic samples). The sharp increase in DBNPA signal could not be attributed to human error or equipment malfunction (Fig. S3 and S4). It is possible that a coeluting compound was absorbed in the same region or interfered with the HPLC-DAD measurement, which could explain the spike at day 14 (Fig. S4) due to chromophores and/or similar degradation products that may not be distinguishable with this method (50). However, DBNPA nondetection was achieved by all HF− biotic microcosm sets (28 days for EE, 49 days for WE, and 56 days for DR), while only one HF+ biotic microcosm set reached nondetection (56 days for NH). Conversely, only one HF− abiotic microcosm set reached DBNPA nondetection (49 days for EE), and only one HF+ abiotic microcosm set reached nondetection (56 days for NH). These observations indicate that HF− microbial communities were better at tolerating and degrading DBNPA.

DBNPA degradation has been documented previously (10, 13, 15). It was shown by others that degradation rate of this biocide is pH dependent, with degradation rates inversely proportional to pH (10, 15). In this study, HF+ streams had an average pH of 4.9 ± 0.13, HF− streams had a pH of 6.5 ± 0.46 (Table S15), and HF− microcosms depleted DBNPA faster than HF+ microcosms, which agrees with the pH-dependent degradation trends previously reported (10, 15). The only biotic HF+ microcosm set to reach nondetection was NH, which had the least acidic pH of the set (Table S15). However, pH-based hydrolysis was not the only factor contributing to degradation, as abiotic microcosms were not able to reach nondetection at the same speed, indicating that microbial biodegradation also played a role.

To evaluate whether a contaminant or degradation product with similar absorbance and retention time as DBNPA may be contributing to the DBNPA signal, the biotic and abiotic samples from days 0, 7, 14, 21, and 28 from the HF+ sites (AB and LL) and also from two HF− sites for comparison (WE and EE) were analyzed using nano-HPLC–high-resolution mass spectrometry. High mass accuracy measurements (± 5 ppm) and fragmentation data from liquid chromatography-mass spectrometry (LC-MS) were used to qualitatively evaluate the results, first by searching for DBNPA and known degradation products and then by comparing the number of brominated compounds detected. Then, relative abundance values and integrated peak area were used to evaluate the trends of these compounds across the five time points within each sample set. The DBNPA molecular ion ([M+H] ^+^ = 240.8606 *m/z*) was not detected in most of the samples analyzed, which may be due to prolonged storage or to multiple freeze-thaw cycles (each sample experienced 2 freeze-thaw cycles). However, because bromine (Br) has a unique isotopic signature (Fig. S5), multiple other brominated species were observed; some of these were known DBNPA degradation products, but many were previously unreported species and potentially novel brominated degradation products (Table S13). Across the four sites (WE, EE, AB, and LL), five time points (days 0, 7, 14, 21, and 28), and two microcosm conditions (biotic or abiotic) analyzed (*n* = 40), 18 brominated species were observed, including DBNPA and four known degradation products, CAM, MBNPA, DBAN, and DBAM (Fig. S1, Table S13). The detected mass to charge ratio, predicted elemental formula, and putative structure of some of these brominated products are described in Table S13. More brominated species were detected in the abiotic samples (an average of 14.1 ± 2.8) compared to those in the biotic samples (11.7 ± 4.4) (Fig. S6). There were also more brominated species in the biotic HF− samples (13 ± 5.6) than in the biotic HF+ samples (10.4 ± 3.3) and more in the abiotic HF− samples (15.2 ± 3.5) than in the abiotic HF+ samples (13 ± 1.7) (Fig. S6). The number of brominated compounds increased through time in all samples (Fig. 4), indicating the formation of by-products of degradation or the reaction of bromide with available organics in the water. Similar to the trend observed by HPLC-DAD, the number of brominated species detected by LC-MS in the HF+ abiotic samples (AB and LL) increased sharply from day 0 to day 14 (Fig. S6). The total “brominated signal” (summed, integrated peak areas at each time point) also increased sharply at day 14 in the abiotic HF+ samples (Fig. S7). While not as strong, the brominated signal also increased at day 14 in the two abiotic HF− samples. For the biotic samples, a steady increase in brominated signal over time was observed regardless of the microcosm, with the highest signal occurring at day 21. The qualitative trends are consistent with the initial HPLC-DAD measurement, which suggests that these brominated degradation products may indeed have impacted the signal response in the initial measurement.

**FIG 5:**
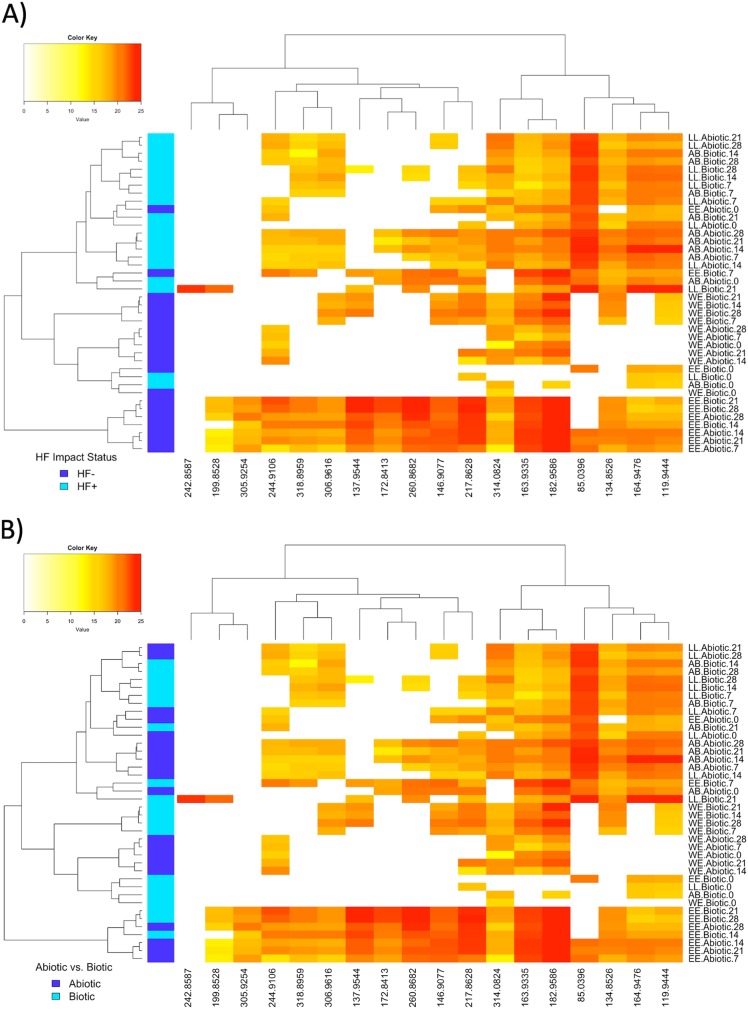
Heat maps of the normalized log_2_ peak areas for brominated species detected by nano-HPLC-HRMS. The dendrograms cluster samples using the Ward method of agglomeration. Rows represent samples (described by stream location, condition, and day of collection), and columns represent *m/z* ratios of the brominate species detected. The top dendrogram is clustered by brominated species that varied similarly across the data set. (A) The left dendrogram clusters first by HF-unimpacted (HF−; dark blue) or HF-impacted (HF+; light blue) microcosms, and then by abiotic and biotic microcosms. (B) The left dendrogram is color coded by abiotic (dark blue) and biotic (light blue) samples.

MBNPA and CAM, two known degradation products of the less-toxic degradation pathway (Fig. S1), were detected in both abiotic and biotic HF+ microcosms (AB and LL) and in one HF− microcosm (EE). DBAN, a toxic pathway degradation product (Fig. S1) was detected in the biotic LL microcosms (HF+) and in abiotic and biotic EE microcosms (HF−), while DBAM, another toxic degradation pathway product, was detected in both biotic AB and LL (HF+) and only abiotic LL, and both abiotic and biotic WE and EE (HF−). Others have shown that the preference for one degradation pathway is dependent on total organic carbon (TOC) content and that higher TOC selects for the less toxic pathway, with MBNPA as an intermediate (13). It is documented that HF+ streams in Pennsylvania, including AB and LL, have higher dissolved organic carbon than HF− streams due to land clearing practices from well pad development (16). Here, mean TOC (Table S14) at day 0 was significantly higher in HF+ samples (7.81 ± 1.11 mg/liter) than in HF− samples (4.09 ± 0.95 mg/liter; *t* test, *P* = 0.02), which could explain the presence of the nontoxic pathway intermediates at the HF+ microcosms, TOC could also be reacting with bromine left after complete DBNPA degradation. Other factors to consider include the different enzymatic capabilities of the microbial communities present within the samples or different water chemistries favoring one pathway over another. The water chemistry measured *in situ* (Table S15) was reported previously, as follows: temperature (HF+: 16.8°C ± 1.96; HF−: 12.8°C ± 0.58), pH (HF+: 4.9 ± 0.13; HF−: 6.5 ± 0.46), conductivity (HF+: 29.2 ± 3.67μS/cm; HF−: 33.7 ± 5.66 μS/cm), and total dissolved solids (HF+: 20.8 ± 2.80 mg/liter; HF−: 23.9 ± 4.01 mg/liter) (28). Even though the differences in these parameters were not significantly different between HF+ and HF− streams, the differences in pH may affect the stability of DBNPA, as discussed previously. This observation is also supported by cluster analysis, as the detected brominated species clustered by HF impact history (Fig. 5A). Samples also clustered by biotic and abiotic conditions by stream, indicating that microbial presence affected the degradation by-products (Fig. 5B). Overall, these results suggest that DBAM and other brominated species may be persistent degradation products of DBNPA that, depending on the history of the watershed, may be preferentially selected over the desired less-toxic pathway with MBAN and CAM as intermediates. More DBNPA degradation kinetic experiments are needed to better understand the conditions dictating intermediate formation.

### Environmental implications.

Our findings demonstrate that previous HF exposure causes surface water microbial communities to respond differently than HF-unimpacted communities to the biocide DBNPA. HF exposure history, and its effect in water chemistry and microbial interactions, may also affect the formation of DBNPA brominated degradation products. In a similar experiment using glutaraldehyde, distinct microbial communities were enriched in HF+ and HF− microcosms after glutaraldehyde perturbation. In the glutaraldehyde experiment, the HF+ microbial community showed higher tolerance to glutaraldehyde based on higher diversity and a smaller log fold decrease of the 16S rRNA gene concentration, but the HF− microbial community was able to degrade glutaraldehyde faster. The faster glutaraldehyde biodegradation in HF− microcosms was attributed to biotic-abiotic interactions, as HF+ microcosms had more acidic pH than HF− microcosms (28) because glutaraldehyde activity is enhanced in alkaline pH (51). Alkaline pH forms more reactive sites in the cell wall, and this effect allows more bacteria to be susceptible to glutaraldehyde while depleting the glutaraldehyde in solution as it cross-links with the bacterial wall (51).

DBNPA caused a different microbial response than the biocide glutaraldehyde. First, HF− microcosms were better at tolerating DBNPA, based on initial 16S rRNA log fold change, which is opposite to what was observed with glutaraldehyde. This could be in part due to DBNPA’s faster hydrolysis at increasing pH, causing HF− microcosms to deplete DBNPA faster than the more acidic HF+ microcosms. Second, even though similar microbial groups were enriched, a more diverse microbial population was able to resist DBNPA compared to glutaraldehyde, as *Methylobacterium* enrichment represented up to 92% of glutaraldehyde microcosms (28). The difference in microbial response may be caused by the DBNPA fast-kill approach, where its biocidal activity is more potent at the moment of initial contact, while glutaraldehyde works over a period of days to weeks. However, both DBNPA and glutaraldehyde depletion was faster in HF− microcosms.

This study revealed that DBNPA and associated degradation products can be persistent in stream water. TOC could have a role in the formation of degradation products. These findings are of importance, as environmental persistence may further hinder the return of the microbial communities to preimpacted states, affecting the nutrient cycle and further retarding microbial natural biodegradation capabilities (i.e., microbial attenuation) in the environment, potentially requiring intervention to stimulate the affected area to enhance the preference for DBNPA nontoxic degradation pathways. Environmental persistence of the brominated disinfectant by-products can cause harm to the public and environmental health. For example, the persistence of these disinfectant by-products may affect ecosystem function, e.g., microbial primary production and natural attenuation, which could have unknown cascading effects to higher trophic levels (10, 52–55). Broad HF impacts have already been shown to affect micro- and macroinvertebrates, fish, and other aquatic organisms in the streams used as source water for the microcosms (16, 56).

Many of the taxa enriched in this study have been previously reported as being capable of degrading or cometabolizing xenobiotic compounds. Although a genetic pathway for microbial biodegradation of DBNPA has not been determined, as it is a halogenated compound, it is likely that the aerobic degradation pathways would involve cometabolism, aerobic assimilation, or dehalogenation (57, 58). In the nontoxic degradation pathway of DBNPA (Fig. S1), the bromines are substituted by hydrogen, which could be achieved by microbial reductive dehalogenation (59) and by abiotic mechanisms. For example, AB- and LL (HF+ streams)-derived microcosms showed intermediates of the less toxic degradation pathway in both biotic and abiotic conditions, but MBNPA was an order of magnitude higher in biotic conditions (Fig. 5B), leading to the conclusion that microbial biodegradation is active and rapid compared to abiotic degradation alone. Further research is needed to understand which microbes can use DBNPA as a carbon source, electron donor, or electron acceptor in metabolism.

Additional research is needed to determine a complete degradation pathway, including quantification of all brominated intermediates, and to better understand when one DBNPA degradation pathway is preferred over the other to adequately handle a HF chemical spill containing DBNPA. Furthermore, differences in degradation kinetics of DBNPA and associate degradation products between HF+ and pristine watersheds should be determined to quantify and determine under what conditions HF+ microbial communities are more efficient at debrominating DBNPA and its degradation by-products. DBNPA may not persist in the environment, but its brominated degradation products, such as DBAN, have a longer half-life and could be more harmful to the public and environmental health.

## MATERIALS AND METHODS

### Stream selection and sample collection.

For comparison purposes, sample collection was identical and done at the same time as that of Campa et al. (28). Briefly, sample selection employed GIS surveys and the Pennsylvania Department of Environmental Protection (PADEP) records to minimize watershed variation caused by industrial activities other than UOG extraction. Streams selected were in forested areas, with no indication of past mining activity or other anthropogenic impacts in the PADEP records. HF-impacted (HF+) streams had active UOG wells within the watershed. These streams were Alex Branch (AB), Little Laurel (LL), and an unnamed tributary to Naval Hollow (NH). AB and LL had reported surface spills (60–62). The spills occurred in 2009 when a pipe carrying flowback water burst, leaking into LL and to a lesser extent into AB. In the same year, HF chemicals were accidentally spilled into AB. The three HF unimpacted (HF−) streams had construction development involving well pads, but no HF activity had started. These streams were unnamed tributary East Elk (EE), an unnamed tributary to West Elk (WE), and Dixon Run (DR). Refer to Table S15 for geological coordinates of the watersheds. A detailed description of the sites, screening process, and selection has been described previously (11–13, 56, 63).

Collection of stream water from three HF+ and three HF− streams in northwestern Pennsylvania occurred in June 2015 under low-flow conditions. Samples were collected in sterile Nalgene bottles and stored at 4°C until use. Conductivity, pH, temperature, and total dissolved solids were measured at collection time using a weekly calibrated Eutech PCSTestr 35 multiparameter test probe.

### Microcosm setup.

Dow Chemicals’ literature showed effective killing (>6-log reduction) of acid-producing bacteria and sulfate-reducing bacteria using 25 mg/liter of DBNPA (24); nevertheless, biocide usage in HF is highly variable, with reports of between 10 to 800 mg/liter (6). Thus, microcosms were constructed using 125 mg/liter DBNPA in 235 ml of stream water. DBNPA was purchased from Sigma-Aldrich (CAS 10222-01-2). Abiotic controls were autoclaved to kill all microbes present and were used to measure abiotic degradation of DBNPA. Negative biological controls (no DBNPA added) were used to examine the bottle effect in microbial communities with no biocide added. Abiotic and biological controls were set at a volume of 20 ml. All microcosms were set in triplicates at ambient temperature (∼21°C) under aerobic conditions and minimal light exposure for 56 days. Microcosms were uncovered only for sampling events and were shaken before sampling. Samples were collected every 7 days for chemical analysis and on days 0, 7, 21, 35, 49, and 56 for microbial analyses. TOC was measured before the beginning of the experiment using a Shimadzu TOC-L Series analyzer with an ASI-L autosampler (Shimadzu, Kyoto, Japan) following the protocol described in Campa et al. (28).

### Quantification of bacterial 16S rRNA gene.

DNA was collected by filtering 25 ml of microcosm water through a 0.2-μm nylon filter (Sterivex), and frozen at –20°C until use. The frozen filter was cut with sterile pliers. The filter membrane was cut with a sterile razor, and DNA was extracted from the membrane using a Mo Bio PowerSoil DNA isolation kit following the manufacturer’s specifications. Bacterial primers Bac1055YF and Bac1392R were used to quantify the 16S rRNA gene in a QuantStudio 12K Flex real-time PCR system (Thermo Fisher Scientific). For reaction mixture and quantitative PCR (qPCR) parameters refer to Campa et al. (28).

### 16S rRNA gene amplicon library preparation, sequencing, and data analyses.

After DNA extraction, the v4 region of the 16S rRNA gene was amplified using the primers and protocol described previously (64). Refer to Campa et al. (28) for the description of library preparation. The final libraries were run on an Illumina MiSeq instrument (San Diego, CA, USA) using a v2 2 × 150-bp read kit following the manufacturer’s specifications.

Data analyses were done in QIIME (v1.9.1) following the protocol described in Campa et al. (28). Briefly, after joining forward and reverse reads and demultiplexing, quality filtration was set to an average *Q* score of more than 19. *De novo* and reference-based chimera detection was done using UCHIME in the USEARCH package (65, 66). OTU picking was done using the Greengenes database (May 2013 version) (67) and applying a 97% sequence identity cutoff using UCLUST (65). Representative sequences for each OTU were aligned using the PyNAST method (68), and taxonomy was assigned using the RDP classifier (69). The OTU table was filtered further to remove sequences with counts below 0.005%, any samples with fewer than 1,000 sequences were discarded, and the OTU table was cumulative-sum scaling (CSS) normalized (70) for beta diversity and weighted UniFrac distance matrix calculation (71). A weighted UniFrac distance matrix was visualized using a directional principal coordinate analysis (PCoA) in EMPeror (72) and forcing the *x* axis by days. The OTU table and weighted Unifrac matrix were then imported into R, and the packages phyloseq (73) and vegan (74) were used for statistical analyses as described below. An unnormalized OTU table was also exported into R for alpha diversity and DESeq2 analyses (75, 76). Difference in alpha diversity metrices—Chao 1, Simpson, Shannon, and observed species—were computed using the package phyloseq (73) to understand the differences in evenness and richness between HF-impacted and HF-unimpacted microcosms before and after DBNPA addition. Statistical analyses were performed as described in the next section.

The DESeq2 (76) R package was used to identify differentially enriched taxa through time and between HF+ and HF− microcosms at each time point (days 7, 21, 35, 29, and 56) compared to day 0 no-DBNPA-added controls. Day 56 DBNPA-added microcosms and day 56 no-DBNPA-added controls were compared as well. For each time point, comparisons between HF impact status were also made. For each comparison, a Wald test was performed using the parametric fit type, and the *P* value was adjusted using the Benjamini and Hochberg method. Reported OTUs had a Bonferroni adjusted *P* value of <0.01 and a calculated ≥2-log_2_ fold change.

### Statistics.

For comparison purposes, statistical analysis was similar to that in Campa et al. (28). To understand the effect of DBNPA on microbial community, 16S rRNA gene abundance was compared using a complete randomized design (CRD) with a split plot using impact status (HF+ versus HF−) as the whole-plot factor and time (days) as the split-plot factors using a mixed effect analysis of variance (ANOVA) model in the R package nlme (77). The least-squares means were computed and separated with the Bonferroni method using the R package emmeans (78). 16S rRNA gene copies/ml were log10 transformed to meet normality and variance assumptions for ANOVA. To compare the no-biocide control at day 0 and at the end of the experiment (day 56), the same model was used. To determine the differences between HF+ and HF− microcosms at day 0, an independent-sample *t* test was performed with data for only that time point. Microbial community alpha diversity (Chao 1, Simpson, Shannon, and observed species) values were rank transformed and compared using the same model as for 16S rRNA gene copies/ml. Finally, a microbial community beta diversity weighted UniFrac distance matrix was used to compare temporal differences between HF+ and HF− microcosms before and after DBNPA addition by applying a nested PERMANOVA with 999 permutations using the adonis command in the R package vegan (74). All statistical tests were performed using R, and *P* value significance was set at *P* = 0.05. See Supplemental Methods for R scripts used.

### Quantification of DBNPA using HPLC-DAD.

Every week, 1 ml of microcosm water was collected to compare the difference between the rates of abiotic and biotic DBNPA degradation in HF+ and HF− microcosms. After collection, samples were filter sterilized using a 0.2-μm nylon filter, acidified to pH 2.5 with phosphoric acid to minimize hydrolysis of DBNPA as described by Blanchard et al. (13), and then frozen at –20°C until analysis.

DBNPA quantification was performed with an Agilent 1200 HPLC system using a modified method described by Blanchard et al. (13). An Agilent Eclipse XDB-C_18_ column (5 μm, 4.6 × 150 mm) was used for separation, with a flow rate of 1 ml/min, and a diode array detector (DAD) was set at 210 nm for detection. The mobile phases and elution gradient were as follows: the initial composition was 75% deionized water (adjusted to pH 2.5 with phosphoric acid; eluent A) and 25% acetonitrile (eluent B), and eluent B increased linearly to 60% over 6 min and further to 85% over an additional 4-min time period. Eluent B was held at 85% for 1 min before the column was equilibrated to initial conditions.

### Detection of DBNPA degradation products using nano-high-performance liquid chromatography–high-resolution mass spectrometry.

Filtered stream water samples were kept frozen in amber bottles in the dark at –20°C until analysis by nano-HPLC-HRMS. Measurements were collected using a Dionex UltiMate 3000 HPLC pump (Thermo Fisher Scientific) coupled to an LTQ-Orbitrap Velos Pro mass spectrometer (Thermo Fisher Scientific) equipped with a nano-electrospray ionization (ESI) source (Proxeon, Denmark) operated in positive mode under direct control of XCalibur software v2.2 SP1.48 (Thermo Fisher Scientific). The nano-electrospray column/emitter was prepared manually in-house using 100-μm interior diameter fused silica (Polymicro Technologies), which was laser pulled and pressure packed to 20 cm with Kinetex C18-RP material (5 μm, 100 Å; Phenomenex). The column was aligned in front of the MS capillary inlet, and 300 nl of the sample was manually injected directly onto the column. LC-MS-grade acetonitrile (ACN) and water (both degassed) were purchased from EMD Millipore, and formic acid (FA) was purchased from Sigma-Aldrich. Nano-flow rates were achieved with a split-flow setup prior to the injection loop (∼250 nl · min^−1^ at the nanospray tip) and separations were conducted by initially holding at 100% A (95% ACN/5% H_2_O/0.1% FA) for 5 min, increasing linearly over 60 min to 100% B (70% ACN/30% H_2_O/0.1% FA), and then holding at 100% B for 5 min before reequilibrating the column at 100% A for 20 min prior to the next injection.

The mass spectrometer was externally calibrated for mass accuracy on the day of analysis using the positive calibration solution (Pierce, Thermo Fisher Scientific). The ESI source capillary voltage was set to 3.0 kV and the capillary temperature to 275°C. High-resolution full scans were acquired in centroid mode at a resolving power of 30,000 over a mass range of 50 –to 1,000 *m/z*. Fragmentation ion spectrum (MS^2^) data were also collected using collision-induced dissociation [CID; He_(g)_] and a data-dependent acquisition approach on the top 5 most abundant ions in each molecular ion spectrum (MS^1^) full scan. High-resolution (15,000 resolving power) MS^2^ spectra were collected using a 2 *m/z* precursor isolation width and an optimized 30% normalized CID energy for fragmentation. Raw LC-MS data were analyzed using Thermo XCalibur Qual software. Integrated LC peak areas were obtained from the extracted ion chromatograms (10 ppm tolerance).

### Data availability.

Mass spectrometry data were uploaded to the Center for Computation Mass Spectrometry (UCSD) online database MassIVE. The MassIVE identification (ID) number is MSV000082488. Microbial 16S rRNA gene amplicon sequences for both DBNPA-treated and glutaraldehyde-treated microcosms were deposited in NCBI Sequence Read Archive (SRA) under SRA accession number SRP151211, BioProject accession number PRJNA476929, and BioSample accession numbers SAMN09459387 to SAMN09459570 and SAMN09475542 to SAMN09475579.

## Supplementary Material

Supplemental file 1
