# Supplementary material for: Surface Water Microbial Community Response to the Biocide 2,2-Dibromo-3-Nitrilopropionamide, Used in Unconventional Oil and Gas Extraction
Source: Appl Environ Microbiol. 2019 Oct 16;85(21):e01336-19. doi: 10.1128/AEM.01336-19 (PMC6803298; doi:10.1128/AEM.01336-19)
Supplement: Supplemental file 1 [file AEM.01336-19-s0001.pdf]

Supporting Information for: Surface water microbial community response to the biocide 2-2-dibromo-3-nitrilopropionamide used in unconventional oil and gas extraction

Maria Fernanda Campa[1,2], Stephen M. Techtmann[3], Mallory P. Ladd[1,4], Jun Yan[5,6], Megan Patterson[6], Amanda Garcia de Matos Amaral[6], Kimberly E. Carter[7], Nikea Ulrich[8], Christopher Grant[8], Robert L. Hettich[1,4], Regina Lamendella[8], Terry Hazen[*,1,2,6,7,9,10].

[1] Bredesen Center for Interdisciplinary Research and Graduate Education, University of Tennessee, Knoxville, TN.

[2] Biosciences Division, Oak Ridge National Laboratory, Oak Ridge, TN.

[3]Department of Biological Sciences, Michigan Technological University, Houghton, MI

[4] Chemical Sciences Division, Oak Ridge National Laboratory, Oak Ridge, TN

[5] Key Laboratory of Pollution Ecology and Environmental Engineering, Institute of Applied Ecology, Chinese Academy of Sciences, Shenyang, Liaoning, P.R. China

[6] Department of Microbiology, University of Tennessee, Knoxville, TN

[7] Department of Civil and Environmental Engineering, University of Tennessee, Knoxville, TN

[8] Department of Biology, Juniata College, Huntingdon, PA

[9] Earth & Planetary Sciences, University of Tennessee, Knoxville, TN

[10] Institute for a Secure and Sustainable Environment, Knoxville, TN.

*Corresponding Author, e-mail: tchazen@utk.edu. Phone: 865-974-7709. Address: 507 SERF, University of Tennessee, Knoxville, TN 37996-1605

Table of Contents:

Supplemental Methods

R code used for 16S rRNA gene abundance statistics

R code used for Alpha diversity statistics

R code used for weighted UniFrac distance statistics

Figure S1: DBNPA and known degradation products.

Figure S2: Microbial Community Shifts Over Time. A) Phylum, B) Genus

Figure S3: Biotic and abiotic degradation of DBNPA over time. Data is shown averaged by HF+ and HF-.

Figure S4: Biotic and abiotic DBNPA degradation over time. Data is shown by water source location.

Figure S5: High-resolution mass spectrum of DBNPA standard.

Figure S6: Number of brominated species detected by nano-HPLC-HRMS in two HF- (left) and two HF+ (right) sets of microcosm samples, biotic and abiotic, from days 0, 7, 14, 21, and 28.

Figure S7: Summed peak areas for all brominated compounds at each time point (0, 7, 14, and 28 days), normalized to each sample set (stream), analyzed by nano-HPLC-HRMS.

Table S1: DESeq2 results, OTU enrichment 7 days after glutaraldehyde addition

Table S2: DESeq2 results HF- vs HF+ enrichment at day 7

Table S3: DESeq2 results, enriched OTU at day 21 vs 0

Table S4: DESeq2 results, enriched OTU at day 35 vs 0

Table S5: DESeq2 results, enriched OTU at day 49 vs 0

Table S6: DESeq2 results, enriched OTU at day 56 vs 0

Table S7: DESeq2 results HF- vs HF+ enrichment at day 21

Table S8: DESeq2 results HF- vs HF+ enrichment at day 35

Table S9: DESeq2 results HF- vs HF+ enrichment at day 49

Table S10: DESeq2 results HF- vs HF+ enrichment at day 56

Table S11: DESeq2 results day 56 no-GA vs day 0 no-GA

Table S12: DESeq2 results day 56 vs day 56 no-DBNPA

Table S13: Putative DBNPA brominated degradation products detected by nano-HPLC-HRMS.

Table S14: Total Organic Carbon (TOC) concentration in source water prior to DBNPA addition.

Table S15: Geological coordinates and watershed physiochemical parameters.

Supplemental References

Supplemental Methods

R code used for 16S rRNA gene abundance statistics

```
library (phyloseq)
library (ggplot2)
library(vegan)
library (nlme)
library(lmerTest)
library(lme4)

##Goal: to do a two-way ANOVA to compare HF+ and HF- 16S rRNA log fold
change

qPCR_dbnpa<-read.csv("qPCR_DBNPA_metadata_12152017.csv",header=T)
head(qPCR_dbnpa)
qPCR_dbnpa$Impacted<-as.factor(qPCR_dbnpa$Impacted)
summary(qPCR_dbnpa$Impacted)
qPCR_dbnpa$Days<-as.factor(qPCR_dbnpa$Days)
summary(qPCR_dbnpa$Days)
qPCR_dbnpa$Sample_Name<-as.factor(qPCR_dbnpa$Sample_Name)
summary(qPCR_dbnpa$Sample_Name)
qPCR_dbnpa$Location<- as.factor(qPCR_dbnpa$Location)
summary(qPCR_dbnpa$Location)
qPCR_dbnpa$Mean<- as.numeric(qPCR_dbnpa$Mean)
summary(qPCR_dbnpa$Mean)

####Comparision between timepoint 0####
T0=subset(qPCR_dbnpa,subset = Days=="0")
HFP=subset(T0, subset = Impacted=="HF+")
HFN=subset(T0, subset = Impacted=="HF-")

HFP_mean<-HFP$Mean
HFN_mean<-HFN$Mean

HFP_mean_num<- as.numeric(as.character(HFP_mean))
HFN_mean_num<- as.numeric(as.character(HFN_mean))

t.test(HFP_mean_num, HFN_mean_num, paired=FALSE, var.equal = FALSE,
conf.level =0.95 )
#result= t = -0.21082, df = 14.234, p-value = 0.836, difference not
significant

####Anova day 7 to 56###
subset7_56=subset(qPCR_dbnpa, Days %in% c("7","21", "56"))

#Log 10 transformed data
lme_LOGqPCR_dbnpa <- lme (log(Mean, 10) ~ Impacted*Days,
random=~1|Sample_Name, correlation = corCAR1(form=~Days|Sample_Name),
```

```
                    data = subset7_56, na.action=na.omit)
summary(lme_LOGqPCR_dbnpa)
anova(lme_LOGqPCR_dbnpa)

#This brings the results from Type I to Type III:
anova.lme(lme_LOGqPCR_dbnpa, type="marginal", adjustSigma=FALSE)


##POSTHOC TEST##
library(lsmeans)
library(emmeans)
lsmeansLT(lme_simpson_v2_rank, list(Density ~ Day), adjust = "tukey")
lsmeans(m1, list(pairwise ~ treatment), adjust = "tukey")
lsmeans(m1, list(pairwise ~ size:treatment), adjust = "tukey")
difflsmeans(lme_LOGqPCR_dbnpa, test.effs = "Impacted" , adjust= "bon")

######
lsm<-lsmeans(lme_LOGqPCR_dbnpa,list("Impacted", "Days"))
print(lsm)
print(pairs(lsm, adjust="bon"))

################################
# normality test for residuals##
################################
residual<-resid(lme_LOGqPCR_dbnpa)
hist(residual)
shapiro.test(residual)
sh.test<-round(shapiro.test(residual)$statistic,2)
if (sh.test>=0.90) {
  print("Normality should be satisfactory")
}else{print(paste("Normality is an concern (W=",sh.test,")",sep="" ))}

#####################
#equal variance test#
#####################

resd<-cbind(subset7_56, residual)
aggregate(resd$residual, list(resd$Impacted,resd$Days), function(x)
c(mean = mean(x), sd = sd(x)))
#boxplot by each factor
boxplot(residual~Impacted,data=resd, xlab="Impacted", ylab="residual")
boxplot(residual~Days,data=resd, xlab="Days", ylab="residual")
boxplot(residual~Impacted*Days,data=resd, xlab="Impacted*Days",
ylab="residual")
#generate levene test results
Level<-aggregate(resd$residual, list(resd$Impacted,resd$Days), sd )$x
fold<-ceiling(max(Level)/min(Level))
if (fold<1 ) {
  print("Equal variance should be satisfactory")
}else{if(fold<5)  {print(paste("Equal variance is potentially an issue
(",fold, "-fold std dev difference)",sep="" ))}
```

```
  else {print(paste("Equal variance is a serious issue (",fold, "-fold
std dev difference)",sep="" ))
  }
}

####Comparison 0 and 56c ######

subset0_56c=subset(qPCR_dbnpa, Days %in% c("0","56bc"))

lme_LOGqPCR_dbnpa_Control <- lme (log(Mean, 10) ~ Impacted*Days,
random=~1|Sample_Name, correlation = corCAR1(form=~Days|Sample_Name),
                    data = subset0_56c, na.action=na.omit)
summary(lme_LOGqPCR_dbnpa_Control)
anova(lme_LOGqPCR_dbnpa_Control)

#This brings the results from Type I to Type III:
anova.lme(lme_LOGqPCR_dbnpa_Control, type="marginal",
adjustSigma=FALSE)

#bonferroni correction

lsm<-lsmeans(lme_LOGqPCR_dbnpa_Control,list("Impacted", "Days"))
print(lsm)
print(pairs(lsm, adjust="bon"))
```

R code used for alpha diversity statistics

```
#load libraries
library (nlme)
library(lmerTest)

Alpha<-read.csv("DBNPA_Alpha.csv",header=T)
Alpha<-read.csv("Alpha_BioControlREmoved_Nov92017.csv",header=T)
head(Alpha)
Alpha$Density<-as.factor(Alpha$Density)
summary(Alpha$Density)
Alpha$Day<-as.factor(Alpha$Day)
summary(Alpha$Day)
Alpha$Location<-as.factor(Alpha$Location)
summary(Alpha$Location)

#print table
mytable <- xtabs(~Density+Day+Location, data=Alpha)
ftable(mytable)

#CRD repeated measures
###USEFUL INFO:  Looking at the NLME documentation, corAR1 represents
an autocorrelation structure of order 1.
###The corCAR1 argument represents an autocorrelation structure of
order 1, with a continuous time covariate.
```

```
###In addition, Singer and Willet (2003) note the important
distinction is that corCAR1 allows for unequally spaced
###time covariates or observations, while corAR1 requires equally
spaced time intervals.

#rank transform data
Alpha$rank_Simpson <- rank(Alpha$Simpson)
Alpha$rank_Chao1 <- rank(Alpha$Chao1)
Alpha$rank_Observed <- rank(Alpha$Observed)
Alpha$rank_Shannon <- rank(Alpha$Shannon)

######Comparison between Control day 0 and 56c###########
AlphaControl_subset = subset(AlphaControl,Day %in% c("0", "c"))

#Rank data
AlphaControl_subset$rank_Simpson <- rank(AlphaControl_subset$Simpson)
AlphaControl_subset$rank_Chao1 <- rank(AlphaControl_subset$Chao1)
AlphaControl_subset$rank_Observed <-
rank(AlphaControl_subset$Observed)
AlphaControl_subset$rank_Shannon <- rank(AlphaControl_subset$Shannon)

#Simspon
lmeCONTROLS_simpson_v2_rank <- lme (rank_Simpson ~ Density*Day,
random=~1|SampleID,data = AlphaControl_subset, na.action=na.omit)
summary(lmeCONTROLS_simpson_v2_rank)
anova(lmeCONTROLS_simpson_v2_rank)
##This brings the results from Type I to Type III:
anova_lmeCONTROLS_typeIII_rank_simpson <-
anova.lme(lmeCONTROLS_simpson_v2_rank, type="marginal",
adjustSigma=FALSE)

#Chao1
lmeCONTROLS_Chao1_v2_rank <- lme (rank_Chao1 ~ Density*Day,
random=~1|SampleID,data = AlphaControl_subset, na.action=na.omit)
summary(lmeCONTROLS_Chao1_v2_rank)
anova(lmeCONTROLS_Chao1_v2_rank)
#TYPE III
anova_lmeCONTROLS_typeIII_rank_Chao1 <-
anova.lme(lmeCONTROLS_Chao1_v2_rank, type="marginal",
adjustSigma=FALSE)

#Observed
lmeCONTROLS_Observed_v2_rank <- lme (rank_Observed ~ Density*Day,
random=~1|SampleID,data = AlphaControl_subset, na.action=na.omit)
summary(lmeCONTROLS_Observed_v2_rank)
anova(lmeCONTROLS_Observed_v2_rank)
#TYPE III
anova_lmeCONTROLS_typeIII_rank_Observed <-
anova.lme(lmeCONTROLS_Observed_v2_rank, type="marginal",
adjustSigma=FALSE)

######Comparison between day 7 through 56###########
```

```
AlphaDBNPA_subset = subset(Alpha,Day %in% c("7", "21", "35", "49",
"56"))

#SIMPSON
lme_simpson_v2_rank <- lme (rank_Simpson ~ Density*Day,
random=~1|X.SampleID,data = AlphaDBNPA_subset, na.action=na.omit)
summary(lme_simpson_v2_rank)
anova(lme_simpson_v2_rank)
anova_lme_typeIII_rank_Simpson <- anova.lme(lme_simpson_v2_rank,
type="marginal", adjustSigma=FALSE)
anova_lme_typeIII_rank_Simpson

#CHAO1
lme_chao1_v2_rank <- lme (rank_Chao1 ~ Density*Day,
random=~1|X.SampleID,data = AlphaDBNPA_subset, na.action=na.omit)
summary(lme_chao1_v2_rank)
anova(lme_chao1_v2_rank)
anova_lme2_typeIII_rank_chao1 <- anova.lme(lme_chao1_v2_rank,
type="marginal", adjustSigma=FALSE)
anova_lme2_typeIII_rank_chao1


#Observed
lme_observed_v2_rank <- lme (rank_Observed ~ Density*Day,
random=~1|X.SampleID,data = AlphaDBNPA_subset, na.action=na.omit)
summary(lme_observed_v2_rank)
anova(lme_observed_v2_rank)
anova_lme2_typeIII_rank_observed <- anova.lme(lme_observed_v2_rank,
type="marginal", adjustSigma=FALSE)
anova_lme2_typeIII_rank_observed

#Shannon
lme_shannon_v2_rank <- lme (rank_Shannon ~ Density*Day,
random=~1|X.SampleID,data = AlphaDBNPA_subset, na.action=na.omit)
summary(lme_shannon_v2_rank)
anova(lme_shannon_v2_rank)
anova_lme2_typeIII_rank_shannon <- anova.lme(lme_shannon_v2_rank,
type="marginal", adjustSigma=FALSE)
anova_lme2_typeIII_rank_shannon

###Comparison timepoint 0####

#Chao1
HFP<-T0$Chao1[T0$Density=="HF+"]
HFN<-T0$Chao1[T0$Density=="HF-"]
t.test(HFP, HFN, paired=FALSE, var.equal = FALSE, conf.level =0.95 )


#Simpson
HFP<-T0$Simpson[T0$Density=="HF+"]
HFN<-T0$Simpson[T0$Density=="HF-"]
```

```
t.test(HFP, HFN, paired=FALSE, var.equal = FALSE, conf.level =0.95 )


#Observed
HFP<-T0$Observed[T0$Density=="HF+"]
HFN<-T0$Observed[T0$Density=="HF-"]
t.test(HFP, HFN, paired=FALSE, var.equal = FALSE, conf.level =0.95 )

#Shannon
HFP<-T0$Shannon[T0$Density=="HF+"]
HFN<-T0$Shannon[T0$Density=="HF-"]
t.test(HFP, HFN, paired=FALSE, var.equal = FALSE, conf.level =0.95 )
```

R code used for weighted UniFrac distance statistics

```
library (phyloseq)
library (ggplot2)
library(vegan)

#adonis
#first make data frame from phyloseq object
adonis_data <- as(sample_data(merged_data), "data.frame")
str(adonis_data)

#nested permanova
adonis(w_unifrac_d~HF_History*(biocide/Days), adonis_data, strata=
adonis_data$Days)
```

R code used for differential abundance analyses

```
library (phyloseq)
library (ggplot2)
library(vegan)
library(grid)
library (DESeq2)
```

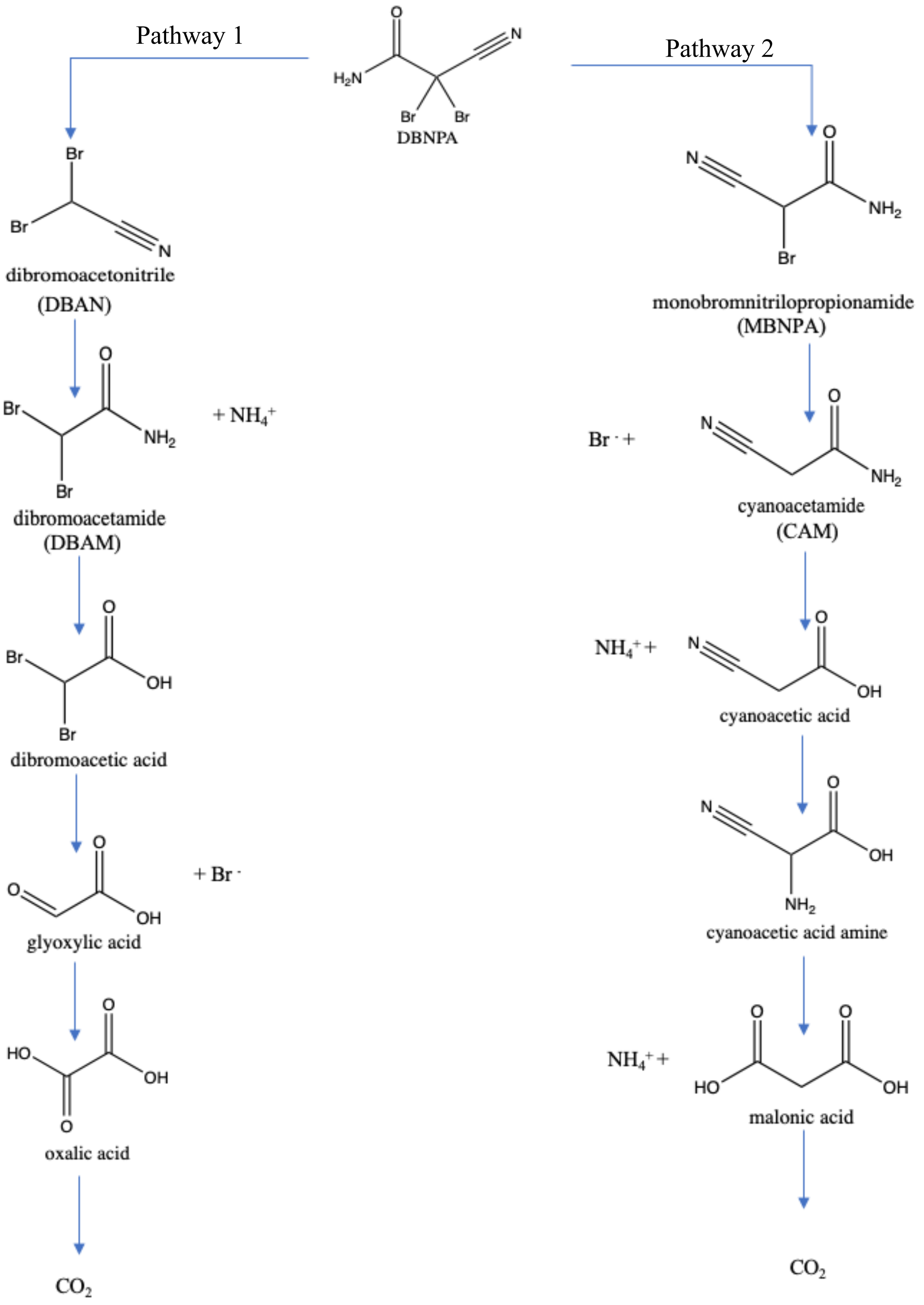

Figure S1: DBNPA and known degradation products. Pathway compiled from literature (Exner et al., 1973; Blanchard et al., 1987). Pathway 2 (MBNPA) is preferred as it is less toxic and recalcitrant than DBAN.

A)

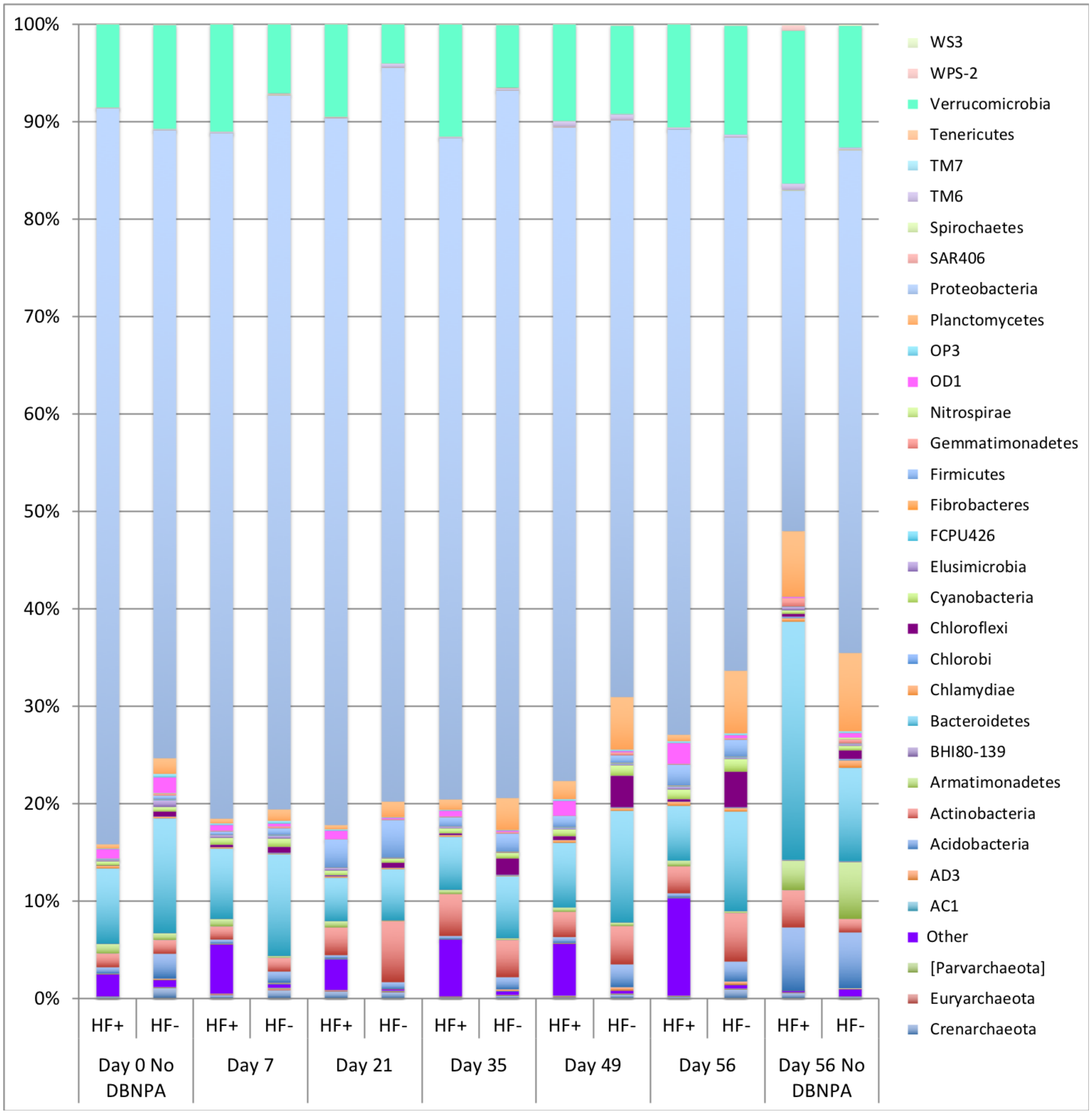
100%
90%
80%
70%
60%
50%
40%
30%
20%
10%
0%
HF+
HF-
Day 0 No DBNPA
Day 7
Day 21
Day 35
Day 49
Day 56
Day 56 No DBNPA
WS3
WPS-2
Verrucomicrobia
Tenericutes
TM7
TM6
Spirochaetes
SAR406
Proteobacteria
Planctomycetes
OP3
OD1
Nitrospirae
Gemmatimonadetes
Firmicutes
Fibrobacteres
FCPU426
Elusimicrobia
Cyanobacteria
Chloroflexi
Chlorobi
Chlamydiae
Bacteroidetes
BHI80-139
Armatimonadetes
Actinobacteria
Acidobacteria
AD3
AC1
Other
[Parvarchaeota]
Euryarchaeota
Crenarchaeota

B)

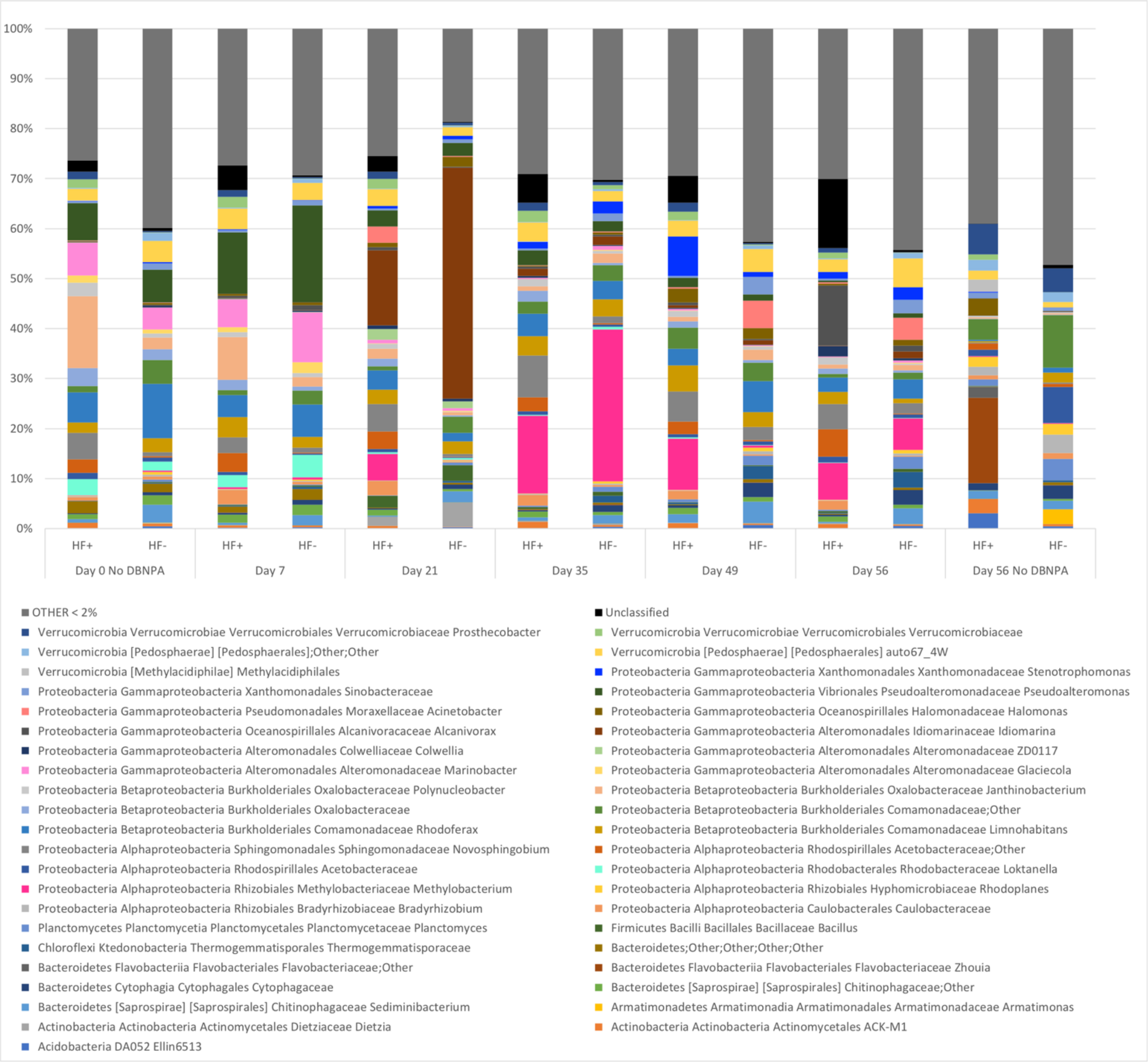

Figure S2. Microbial community taxonomy changes over time. The first set is the microbial community prior to DBNPA addition, then day 7 to 56 after DBNPA addition, and the last set is the no-DBNPA control at day 56. A) Phylum microbial community changes over time. B) Genus level microbial community changes over time.

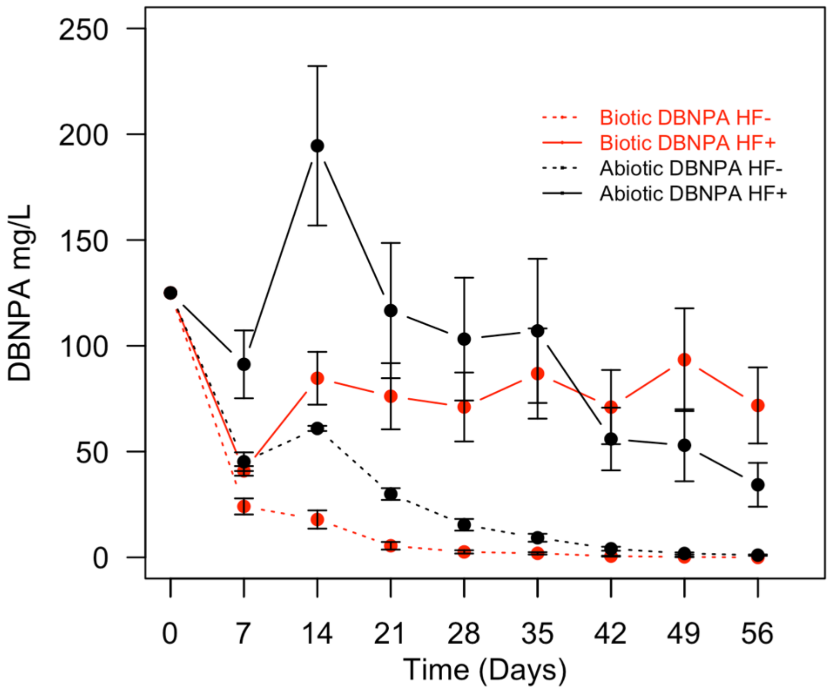

Figure S3. Biotic and abiotic degradation of DBNPA over time. The red lines represent the biotic microcosms while the black lines represent the abiotic. Each dashed line represents the HF- microcosm (n=9, three source streams and three replications) while the solid lines are HF+ microcosms (n= 9, three source streams and three replications). Measurement at time zero could not be established, known added concentration of 125 ppm was used for the figure. Error bars represent one standard error.

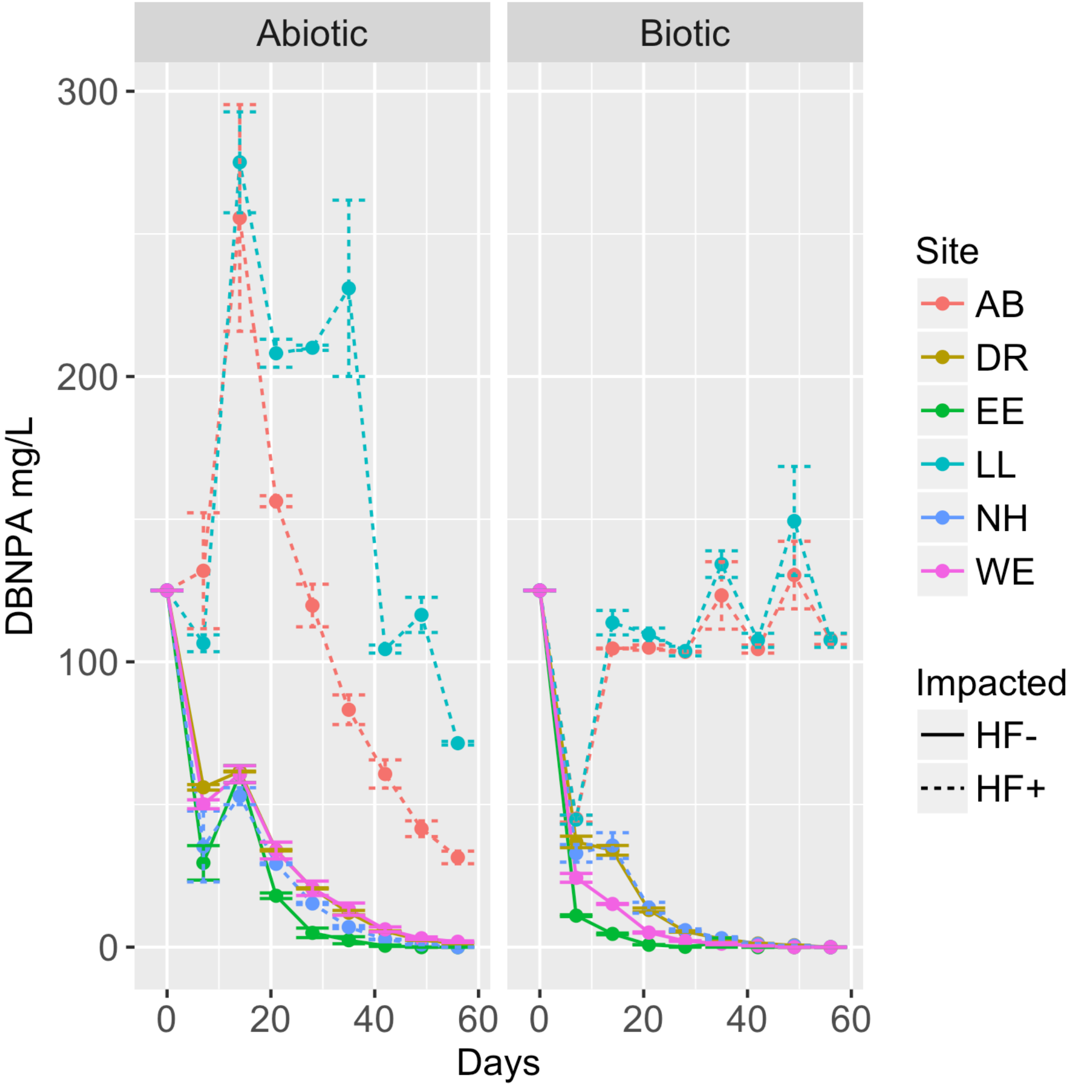

Figure S4. Abiotic and biotic DBNPA degradation over time. Data is presented by water source visualized by different colors. HF-impacted line trends are displayed with a solid line, while HF-unimpacted are displayed with a dashed line. Each data point is n=3, and the error bars represent one standard error. Measurement at time zero could not be established, known added concentration of 125 ppm was used for the figure.

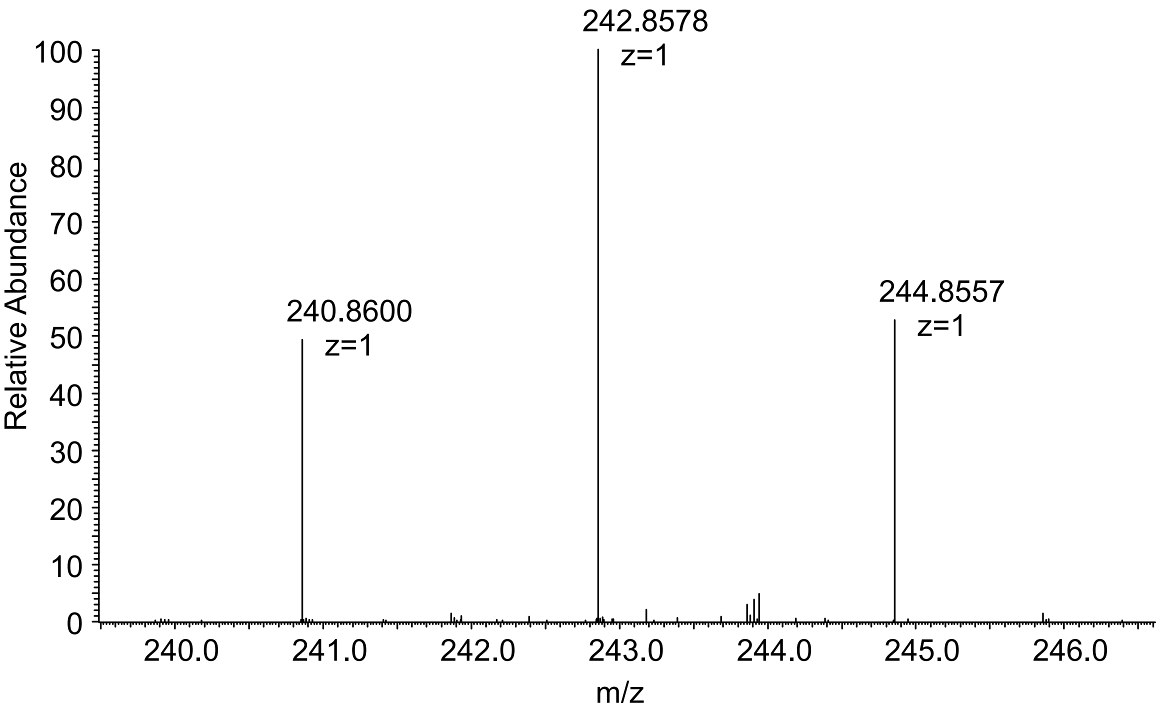

Figure S5. High-resolution mass spectrum of DBNPA standard (monoisotopic mass: 239.8534 Da) collected by direct infusion in positive-ion mode showing characteristic isotopic signature for dibrominated compounds. The monoisotopic ion ($[M+H]^+$) can be seen at 240.8600 *m/z* (Δ2.5 ppm mass error), the M+2 at 242.8578 *m/z*, and the M+4 at 244.8557 *m/z*.

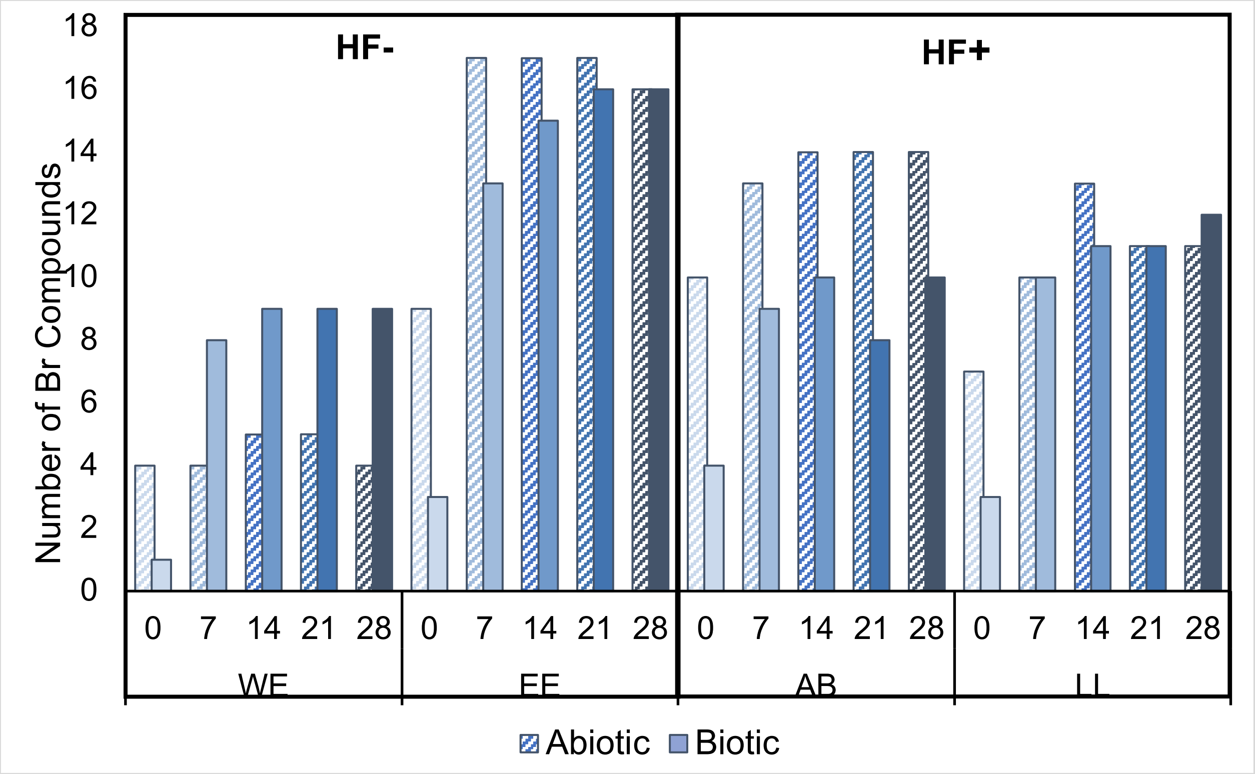

Figure S6. Number of brominated species detected by nano-HPLC-HRMS in two HF- (left) and two HF+ (right) sets of microcosm samples, biotic and abiotic, from days 0, 7, 14, 21, and 28. Abiotic samples are indicated by textured bars and biotic samples are shown with solid color.

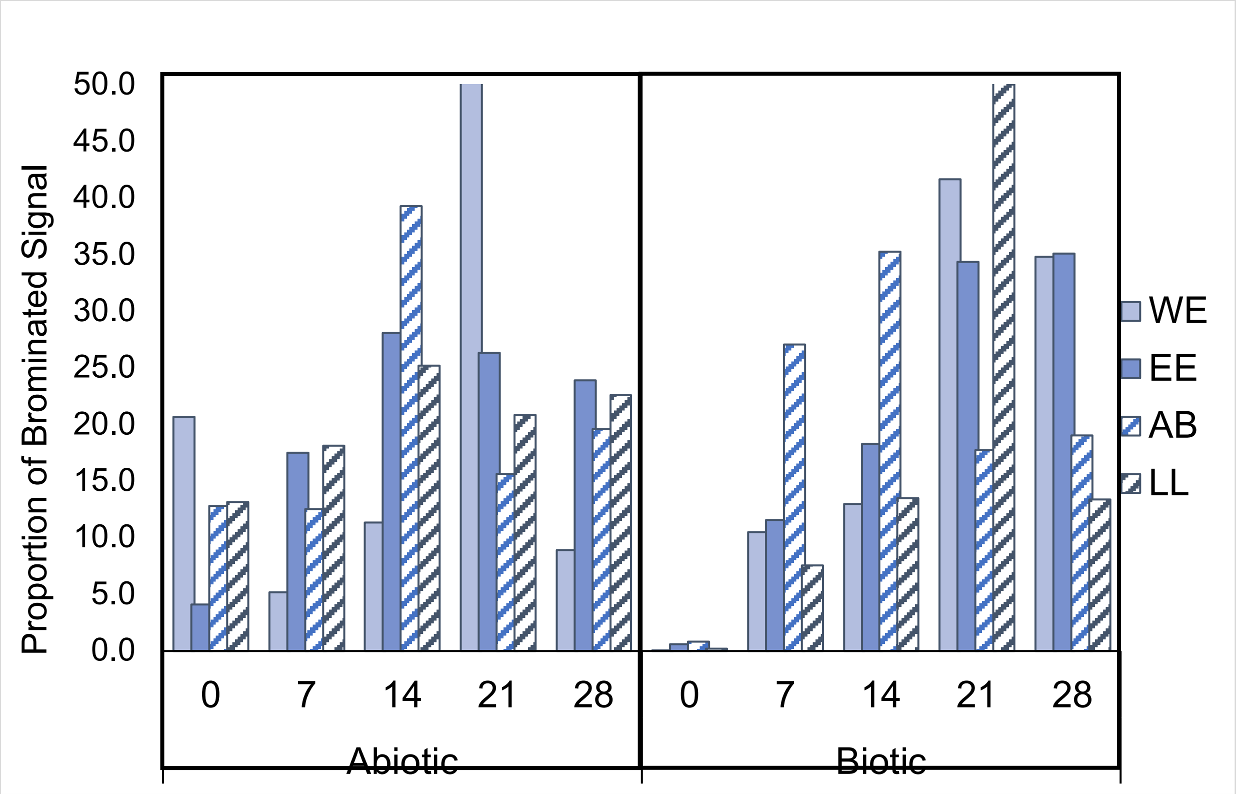

Figure S7. Summed peak areas for all brominated compounds at each time point (0, 7, 14, and 28 days), normalized to each sample set (stream), analyzed by nano-HPLC-HRMS. The abiotic samples are shown on the left and the biotic on the right with the HF+ samples indicated by textured bars and the HF- samples shown with solid color.

Table S1. DESeq2 Results Enrichment Day 7 vs Day 0

OTU	baseMean	log2FoldChange	lfcSE	stat	pvalue	padj	Kingdom	Phylum	Class	Order	Family	Genus
834104	12.5877522	7.431403463	1.63753553	4.5381632	5.67E-06	0.00064691	Bacteria	SAR406	AB16	ZA3648c	AEGEAN_185	
833419	32.0642952	7.265376761	1.30845218	5.55264981	2.81E-08	8.02E-06	Bacteria	Proteobacteria	Deltaproteobacteria	Sva0853	SAR324	
351294	84.1030873	6.923456803	1.17284571	5.90312669	3.57E-09	2.03E-06	Archaea	Euryarchaeota	Thermoplasmata	E2	Marine group II	
562576	18.0772408	6.117587237	1.13555391	5.38731553	7.15E-08	1.63E-05	Bacteria	Proteobacteria	Alphaproteobacteria	Kiloniellales		
279204	9.9668701	5.857000877	1.28322135	4.56429508	5.01E-06	0.00063482	Bacteria	Bacteroidetes	Flavobacteriia	Flavobacteriales	Flavobacteriaceae	Salegentibacter
832374	7.78422476	5.695807069	1.54715798	3.68146444	0.0002319	0.00967429	Bacteria	SAR406	AB16	Arctic96B-7	A714017	Arctic95A-2
1101451	20.4988778	5.660588942	0.92361696	6.12871915	8.86E-10	1.01E-06	Bacteria	Actinobacteria	Actinobacteria	Actinomycetales	Micrococcaceae	Micrococcus
674655	29.9045471	5.652118499	1.08290214	5.21941759	1.79E-07	3.26E-05	Bacteria	Proteobacteria	Alphaproteobacteria	Sphingomonadales	Sphingomonadaceae	Sphingopyxis
838996	12.4737272	5.638653417	1.2964858	4.34918255	1.37E-05	0.00141615	Bacteria	Proteobacteria	Deltaproteobacteria	Desulfobacterales	Nitrospinaceae	Nitrospina
110119	18.5963608	5.572219322	1.31120964	4.24967843	2.14E-05	0.0017432	Archaea	Euryarchaeota	Thermoplasmata	E2	Marine group III	
839363	19.5910696	5.305024325	1.26830094	4.18278039	2.88E-05	0.00195205	Bacteria	Actinobacteria	Acidimicrobiia	Acidimicrobiales	ZA3409c	
248432	76.3841116	5.269313664	1.2310515	4.2803357	1.87E-05	0.00163644	Bacteria	Proteobacteria	Gammaproteobacteria	Oceanospirillales	Oceanospirillaceae	Oleibacter
834856	14.3356926	5.12710727	1.3712443	3.73901812	0.00018474	0.00842416	Bacteria	SAR406	AB16	Arctic96B-7	A714017	SargSea-WGS
1107076	7.25574706	5.046946831	1.32325567	3.81403756	0.00013671	0.00677629	Bacteria	Bacteroidetes	Flavobacteriia	Flavobacteriales	NS9	
557211	59.9172587	5.040727221	0.8720532	5.78029784	7.46E-09	2.83E-06	Bacteria	Cyanobacteria	Synechococcophycideae	Synechococcales	Synechococcaceae	Synechococcus
New.Referen	13.0091762	4.544513723	1.24376192	3.65384538	0.00025834	0.00981699	Bacteria	Verrucomicrob	Verruco-5	WCHB1-41	RFP12	
274442	12.6814303	4.484579391	1.22020979	3.67525276	0.00023761	0.00967429	Bacteria	Tenericutes	Mollicutes	Acholeplasmatales	Acholeplasmataceae	Acholeplasma
103229	17.4392959	4.033190846	1.0732274	3.75800214	0.00017128	0.00813558	Bacteria	Bacteroidetes	Flavobacteriia	Flavobacteriales	Cryomorphaceae	Fluviicola
534401	25.6921651	3.890352736	1.0467592	3.71656895	0.00020195	0.00885458	Bacteria	Bacteroidetes	Cytophagia	Cytophagales	Cytophagaceae	Leadbetterella
1005844	212.74181	3.474558113	0.83116964	4.18032366	2.91E-05	0.00195205	Archaea	Crenarchaeota	Thaumarchaeota	Cenarchaeales	Cenarchaeaceae	Nitrosopumilus
41476	361.373096	3.27334028	0.79532244	4.11573989	3.86E-05	0.00219985	Bacteria	Proteobacteria	Gammaproteobacteria	Oceanospirillales	Alcanivoracaceae	Alcanivorax
539866	56.8671782	3.261065468	0.89043323	3.66233578	0.00024993	0.00981699	Bacteria	Proteobacteria	Gammaproteobacteria	Thiotrichales	Piscirickettsiaceae	
579608	41.268171	3.189871485	0.69617506	4.58199623	4.61E-06	0.00063482	Bacteria	Firmicutes	Bacilli	Lactobacillales	Streptococcaceae	Streptococcus
558264	90.6959621	2.825006752	0.69591424	4.05941796	4.92E-05	0.0026706	Bacteria	Proteobacteria	Betaproteobacteria	Burkholderiales	Alcaligenaceae	Achromobacter
944197	39.0861013	-2.071341929	0.50056567	-4.1380024	3.50E-05	0.00219985	Bacteria	Proteobacteria	Betaproteobacteria	Burkholderiales	Oxalobacteraceae	Herminiimonas
225453	154.172374	-2.291302281	0.56771205	-4.0360289	5.44E-05	0.00281702	Bacteria	Proteobacteria	Gammaproteobacteria	Alteromonadales	Alteromonadaceae	Cellvibrio
646549	313.436754	-2.321757658	0.5376411	-4.3184155	1.57E-05	0.00149296	Bacteria	Proteobacteria	Gammaproteobacteria	Pseudomonadales	Pseudomonadaceae	Pseudomonas
566578	123.483142	-2.690723444	0.63670425	-4.2260177	2.38E-05	0.00180776	Bacteria	Proteobacteria	Betaproteobacteria	Burkholderiales	Comamonadaceae	Limnobacter
791738	82.82309	-3.160496702	0.60787442	-5.1992592	2.00E-07	3.26E-05	Bacteria	Proteobacteria	Betaproteobacteria	Burkholderiales	Comamonadaceae	Polaromonas

Table S2. DESeq2 Results Differentially Enriched HF- vs HF+ at Day 7

OTU	baseMean	log2FoldChang	lfcSE	stat	pvalue	padj	Kingdom	Phylum	Class	Order	Family	Genus
New.Refe	12.3577096	7.144708795	1.43448917	4.98066416	6.34E-07	2.56E-05	Bacteria	Verrucomicr	[Pedosphaerae]	[Pedosphaerales]	Ellin517	
4575	7.55009459	6.418746732	1.55135162	4.13751895	3.51E-05	0.00087644	Bacteria	Proteobacteri	Alphaproteobacteria	Rhodospirillales	Rhodospirillaceae	Oleomonas
4437895	3.71922156	5.412933454	1.65673707	3.26722542	0.00108607	0.00974783	Bacteria	Proteobacteri	Alphaproteobacteria	Sphingomonadale	Erythrobacteraceae	Lutibacterium
1501738	24.6288822	5.280334564	0.87695417	6.02122065	1.73E-09	2.84E-07	Bacteria	Proteobacteri	Betaproteobacteria	Rhodocyclales	Rhodocyclaceae	
576852	17.4820424	4.704351793	1.14619126	4.10433404	4.05E-05	0.00095457	Bacteria	Proteobacteri	Deltaproteobacteria	Desulfobacterales	Desulfobacteraceae	
816208	2.20467988	4.669685327	1.37471788	3.39683174	0.00068171	0.00726727	Bacteria	Acidobacteria				
577244	2.54536141	4.433334505	1.31155397	3.38021509	0.00072429	0.00757823	Bacteria	Proteobacteri	Gammaproteobacteri	Xanthomonadales	Xanthomonadaceae	Lysobacter
New.Refe	5.30869766	3.924034586	1.17588911	3.33707877	0.00084664	0.00839213	Bacteria	BRC1	PRR-11			
335484	5.62024515	3.888548861	0.95231514	4.08325846	4.44E-05	0.00100363	Bacteria	Proteobacteri	Betaproteobacteria	Rhodocyclales	Rhodocyclaceae	Uliginosibacterium
274442	38.5777809	3.865258531	1.18017569	3.2751552	0.00105604	0.00974783	Bacteria	Tenericutes	Mollicutes	Acholeplasmatales	Acholeplasmataceae	Acholeplasma
226364	8.80233276	3.764348833	1.04050483	3.61781004	0.00029711	0.00419663	Bacteria	Acidobacteri	[Chloracidobacteria]	RB41		
876170	31.9584705	3.458993073	0.62503324	5.53409462	3.13E-08	1.96E-06	Bacteria	Actinobacteri	Actinobacteria	Actinomycetales	Microbacteriaceae	Salinibacterium
3038080	26.6866937	3.316351998	0.82993064	3.99593872	6.44E-05	0.00134843	Bacteria	Proteobacteri	Gammaproteobacteri	Alteromonadales	211ds20	
837885	6.75637197	2.957683293	0.74340143	3.97858166	6.93E-05	0.00135069	Bacteria	Firmicutes	Clostridia	Clostridiales	[Tissierellaceae]	Sedimentibacter
998905	28.17695	2.685227643	0.62685465	4.28365272	1.84E-05	0.00057708	Bacteria	Proteobacteri	Alphaproteobacteria	Caulobacterales	Caulobacteraceae	Mycoplana
New.Refe	19.8660156	2.682878375	0.70179706	3.82286922	0.00013191	0.00240413	Bacteria	Proteobacteri	Betaproteobacteria	SC-I-84		
610107	50.7867878	2.660558056	0.77335187	3.44029433	0.00058108	0.00643748	Bacteria	Bacteroidetes	Cytophagia	Cytophagales	Cytophagaceae	Flectobacillus
149505	98.3227391	2.609388593	0.7396667	3.52778974	0.00041904	0.00538092	Bacteria	Proteobacteri	Alphaproteobacteria	Rhodobacterales	Hyphomonadaceae	Oceanicaulis
837424	79.9889241	2.583840215	0.5102042	5.0643256	4.10E-07	1.93E-05	Bacteria	Proteobacteri	Alphaproteobacteria	Rhodobacterales	Hyphomonadaceae	
4458583	6.46435994	2.52466227	0.73202279	3.44888477	0.00056291	0.00643748	Bacteria	Proteobacteri	Betaproteobacteria	Nitrosomonadales		
712582	17.0455398	2.385891098	0.73017849	3.26754504	0.00108485	0.00974783	Bacteria	Fibrobacteres	Fibrobacteria	258ds10		
New.Refe	238.358815	-2.159982956	0.6183632	-3.4930652	0.00047751	0.00586507	Bacteria	Cyanobacteri	Chloroplast	Stramenopiles		
754628	236.400756	-2.231245495	0.38356935	-5.8170589	5.99E-09	6.53E-07	Bacteria	Proteobacteri	Alphaproteobacteria	Rhizobiales	Methylocystaceae	
1106617	4397.60332	-2.299457104	0.62848471	-3.658732	0.00025347	0.00376864	Bacteria	Proteobacteri	Betaproteobacteria	Burkholderiales	Comamonadaceae	Limnohabitans
587098	47.5626325	-2.485915514	0.58832918	-4.225382	2.39E-05	0.00067386	Bacteria	Proteobacteri	Betaproteobacteria	Gallionellales	Gallionellaceae	Gallionella
1049393	171.743421	-2.494567666	0.67630415	-3.6885293	0.00022555	0.00353995	Bacteria	Verrucomicr	[Spartobacteria]	[Chthoniobacterale	[Chthoniobacterace	Chthoniobacter
831824	808.685855	-2.513999444	0.56345279	-4.4617747	8.13E-06	0.00027015	Bacteria	Proteobacteri	Alphaproteobacteria	Rhodospirillales	Acetobacteraceae	
786420	1201.4296	-2.588226489	0.73642002	-3.5146064	0.00044041	0.00552955	Bacteria	Verrucomicr	[Spartobacteria]	[Chthoniobacterale	[Chthoniobacterace	Candidatus Xiphinematobacter
221405	32.7369112	-2.67916839	0.75377649	-3.5543273	0.00037895	0.00509774	Bacteria	Armatimonad	Armatimonadia	Armatimonadales	Armatimonadaceae	
590601	2096.42922	-2.719323752	0.67884187	-4.005828	6.18E-05	0.00134297	Bacteria	Proteobacteri	Betaproteobacteria	Burkholderiales	Oxalobacteraceae	
1039699	88.877164	-2.89916368	0.68466217	-4.2344441	2.29E-05	0.00067386	Bacteria	Armatimonad	Chthonomonadetes	Chthonomonadale	Chthonomonadaceae	
937848	32.8459991	-2.900430595	0.8877956	-3.2670027	0.00108693	0.00974783	Bacteria	Verrucomicr	[Spartobacteria]	[Chthoniobacterale	[Chthoniobacterace	heteroC45_4W
1017063	36.7637617	-2.923562549	0.8221354	-3.5560597	0.00037646	0.00509774	Bacteria	Proteobacteri	Deltaproteobacteria	Myxococcales	Polyangiaceae	
177975	3110.05496	-2.939462653	0.65612383	-4.4800425	7.46E-06	0.00026353	Bacteria	Proteobacteri	Alphaproteobacteria	Sphingomonadale	Sphingomonadacea	Novosphingobium
New.Refe	390.190681	-3.032291374	0.81872259	-3.703686	0.00021249	0.00344038	Bacteria	Verrucomicr	[Methylacidiphilae]	Methylacidiphilale	LD19	
4300564	2096.46007	-3.068412163	0.74227003	-4.133822	3.57E-05	0.00087644	Bacteria	Proteobacteri	Alphaproteobacteria	Caulobacterales	Caulobacteraceae	Asticcacaulis
1068470	22.2743803	-3.079461978	0.6302794	-4.885868	1.03E-06	3.88E-05	Bacteria	Bacteroidetes	Sphingobacteriia	Sphingobacteriales	Sphingobacteriacea	Pedobacter
1060517	6427.81926	-3.123985101	0.79160763	-3.9463808	7.93E-05	0.00149426	Bacteria	Proteobacteri	Betaproteobacteria	Burkholderiales	Oxalobacteraceae	Janthinobacterium
New.Refe	404.166191	-3.456290507	0.59668745	-5.7924638	6.94E-09	6.53E-07	Bacteria	Armatimonad	[Fimbriimonadia]	[Fimbriimonadales	[Fimbriimonadacea	Fimbriimonas
37406	252.957463	-3.61136644	0.68946472	-5.2379278	1.62E-07	9.18E-06	Bacteria	Proteobacteri	Alphaproteobacteria	Rhodospirillales	Rhodospirillaceae	Telmatospirillum
2139822	2768.98709	-3.723425044	0.7249864	-5.135855	2.81E-07	1.44E-05	Bacteria	Proteobacteri	Alphaproteobacteria	Caulobacterales	Caulobacteraceae	
516569	1334.94317	-3.768884424	0.74931568	-5.0297685	4.91E-07	2.13E-05	Bacteria	Verrucomicr	Verrucomicrobiae	Verrucomicrobiale	Verrucomicrobiacea	Prosthecobacter
1028297	67.3806468	-3.883087226	1.10018106	-3.5294983	0.00041635	0.00538092	Bacteria	Verrucomicr	[Spartobacteria]	[Chthoniobacterale	[Chthoniobacterace	Ellin506
845780	1129.17843	-3.985953378	0.6383295	-6.2443509	4.26E-10	1.20E-07	Bacteria	Bacteroidetes	Sphingobacteriia	Sphingobacteriales	Sphingobacteriaceae	
547579	2.59601643	-4.139107672	1.13384635	-3.6505014	0.00026173	0.00379171	Bacteria	Chlamydiae	Chlamydiia	Chlamydiales	Parachlamydiaceae	Candidatus Protochlamydia
1017181	4.24569529	-4.749779282	1.28270709	-3.7029337	0.00021312	0.00344038	Bacteria	Actinobacteri	Actinobacteria	Actinomycetales	Micrococcaceae	Rothia
New.Clear	1969.40868	-4.883524076	0.64639427	-7.5550238	4.19E-14	2.37E-11	Bacteria	Verrucomicr	Verrucomicrobiae	Verrucomicrobiale	Verrucomicrobiaceae	
279204	67.9759646	-5.129791034	0.92179382	-5.5650091	2.62E-08	1.85E-06	Bacteria	Bacteroidetes	Flavobacteriia	Flavobacteriales	Flavobacteriaceae	Salegentibacter
267354	57.6632464	-5.499062084	0.97223012	-5.6561322	1.55E-08	1.25E-06	Bacteria	Proteobacteri	Alphaproteobacteria	Rhodospirillales	Acetobacteraceae	Acidocella
309065	8.53750193	-5.849157083	1.40754738	-4.1555668	3.24E-05	0.00087301	Bacteria	Proteobacteri	Gammaproteobacteri	Xanthomonadales	Xanthomonadaceae	Rhodanobacter
1101451	112.387158	-6.140300238	1.02389364	-5.9970099	2.01E-09	2.84E-07	Bacteria	Actinobacteri	Actinobacteria	Actinomycetales	Micrococcaceae	Micrococcus

404

Table S3. DESeq2 Results Enrichment Day 21 vs Day 0

OTU	baseMean	log2FoldCh	lfcSE	stat	pvalue	padj	Kingdom	Phylum	Class	Order	Family	Genus
539735	3170.82046	11.6962859	0.8562725	13.659537	1.77E-42	9.55E-40	Bacteria	Actinobacteri	Actinobacteria	Actinomycetal	Dietziaceae	Dietzia
562878	31141.408	10.4845027	0.78774971	13.3094339	2.04E-40	5.50E-38	Bacteria	Proteobacteri	Gammaproteob	Alteromonada	Idiomarinaceae	Idiomarina
279220	563.309596	10.4676401	1.17208121	8.93081465	4.23E-19	5.70E-17	Bacteria	Proteobacteri	Gammaproteob	Oceanospirilla	Oceanospirillacea	Amphritea
739614	75.9137413	9.02522786	1.44131877	6.26178472	3.81E-10	1.58E-08	Bacteria	Proteobacteri	Gammaproteob	Vibrionales	Vibrionaceae	Photobacterium
573035	99.5947164	8.0511924	0.98500749	8.17373723	2.99E-16	2.69E-14	Bacteria	Firmicutes	Bacilli	Bacillales	Alicyclobacillacea	Alicyclobacillus
1111294	62.3206755	7.67514692	0.90810243	8.4518515	2.87E-17	3.09E-15	Bacteria	Proteobacteri	Gammaproteob	Enterobacteria	Enterobacteriacea	Escherichia
1033779	5.11039292	7.63107061	2.07347565	3.68032806	0.00023293	0.00181959	Bacteria	Firmicutes	Clostridia	Clostridiales	Peptostreptococca	Tepidibacter
351294	84.1030873	7.52479132	1.17353226	6.41208732	1.44E-10	6.45E-09	Archaea	Euryarchaeot	Thermoplasmat	E2	Marine group II	
833419	32.0642952	7.06010671	1.31082217	5.3860141	7.20E-08	1.49E-06	Bacteria	Proteobacteri	Deltaproteobact	Sva0853	SAR324	
40791	17.5222287	7.0016707	1.55368484	4.50649355	6.59E-06	9.35E-05	Bacteria	Verrucomicro	Verrucomicrob	Verrucomicrol	Verrucomicrobiac	Verrucomicrobium
694558	1961.75205	6.97843771	0.60882708	11.4621013	2.04E-30	3.67E-28	Bacteria	Firmicutes	Bacilli	Bacillales	Bacillaceae	Bacillus
834104	12.5877522	6.94244659	1.64271034	4.2262147	2.38E-05	0.00026142	Bacteria	SAR406	AB16	ZA3648c	AEGEAN_185	
531300	89.6955906	6.70612765	0.98095907	6.83629714	8.13E-12	4.87E-10	Bacteria	Actinobacteri	Actinobacteria	Actinomycetal	Brevibacteriaceae	Brevibacterium
674655	29.9045471	6.68170671	1.09011309	6.12937023	8.82E-10	2.97E-08	Bacteria	Proteobacteri	Alphaproteobac	Sphingomona	Sphingomonadac	Sphingopyxis
838996	12.4737272	6.13411411	1.30082698	4.71554959	2.41E-06	3.71E-05	Bacteria	Proteobacteri	Deltaproteobact	Desulfobacter	Nitrospinaceae	Nitrospina
503387	3.13233422	5.88751941	1.56503812	3.7619016	0.00016863	0.00140153	Bacteria	Firmicutes	Clostridia	Clostridiales	[Tissierellaceae]	Anaerococcus
562576	18.0772408	5.85273846	1.14057149	5.1314087	2.88E-07	5.17E-06	Bacteria	Proteobacteri	Alphaproteobac	Kiloniellales		
839363	19.5910696	5.82977272	1.27103662	4.58662844	4.50E-06	6.74E-05	Bacteria	Actinobacteri	Acidimicrobiia	Acidimicrobia	ZA3409c	
832374	7.78422476	5.6178507	1.55381209	3.61552774	0.00029974	0.00215411	Bacteria	SAR406	AB16	Arctic96B-7	A714017	Arctic95A-2
1016465	5.06499754	5.49610091	1.77876831	3.08983518	0.00200268	0.00955259	Bacteria	Proteobacteri	Alphaproteobac	Rickettsiales	Pelagibacteraceae	
1005844	212.74181	5.39024847	0.83132088	6.48395655	8.93E-11	4.82E-09	Archaea	Crenarchaeot	Thaumarchaeot	Cenarchaeales	Cenarchaeaceae	Nitrosopumilus
646909	584.182097	5.35173376	0.94456188	5.66583714	1.46E-08	3.58E-07	Bacteria	Proteobacteri	Gammaproteob	Alteromonada	Alteromonadacea	ZD0117
990864	9.14215216	5.21534675	1.09438907	4.76553257	1.88E-06	3.08E-05	Bacteria	Proteobacteri	Gammaproteob	Pseudomonad	Moraxellaceae	Enhydrobacter
110119	18.5963608	5.092956	1.31547143	3.87158238	0.00010813	0.00098784	Archaea	Euryarchaeot	Thermoplasmat	E2	Marine group III	
1109254	43.9019109	4.97893728	0.6950821	7.16309241	7.89E-13	5.31E-11	Bacteria	Proteobacteri	Deltaproteobact	Desulfobacter	Desulfobulbaceae	
818854	125.490938	4.96035812	0.85228408	5.82007601	5.88E-09	1.67E-07	Bacteria	Proteobacteri	Alphaproteobac	Rhodobacteral	Rhodobacteraceae	Rhodobacter
837317	11.3479067	4.95670586	1.55136567	3.19505965	0.00139802	0.0071088	Bacteria	Proteobacteri	Gammaproteob	Thiohalorhabd	Thiohalorhabdaceae	
539866	56.8671782	4.94852614	0.89123624	5.55242921	2.82E-08	6.60E-07	Bacteria	Proteobacteri	Gammaproteob	Thiotrichales	Piscirickettsiaceae	
720511	15.3778315	4.89851637	1.24360213	3.93897392	8.18E-05	0.0007738	Archaea	Crenarchaeot	Thaumarchaeot	Nitrososphaer	Nitrososphaerace	Candidatus Nitrososphaera
621037	21.244713	4.84873889	0.81342912	5.96086222	2.51E-09	7.51E-08	Bacteria	Planctomycet	Phycisphaerae	MSBL9		
139771	4.43687176	4.84496901	1.44895828	3.34376019	0.00082651	0.00464052	Bacteria	Proteobacteri	Deltaproteobact	Desulfobacter	Desulfobacterace	Desulfococcus
358001	5.31878492	4.8169132	1.22234411	3.9407178	8.12E-05	0.0007738	Bacteria	Firmicutes	Clostridia	Clostridiales	Ruminococcaceae	Oscillospira
1024520	8.19701342	4.78687499	1.30408861	3.67066698	0.00024192	0.00186277	Bacteria	Proteobacteri	Betaproteobact	Burkholderial	Comamonadacea	Comamonas
834856	14.3356926	4.75341257	1.37656687	3.45309238	0.0005542	0.00351427	Bacteria	SAR406	AB16	Arctic96B-7	A714017	SargSea-WGS
238813	12.7735485	4.53321686	0.781463	5.800936	6.59E-09	1.78E-07	Bacteria	Firmicutes	Clostridia	Clostridiales	Peptococcaceae	Desulfosporosinus
554951	18.7330984	4.53293699	1.33431353	3.3972053	0.00068078	0.00393704	Bacteria	Chloroflexi	SAR202			
248432	76.3841116	4.41655827	1.23249101	3.58344056	0.0003391	0.00234325	Bacteria	Proteobacteri	Gammaproteob	Oceanospirilla	Oceanospirillacea	Oleibacter
814282	44.6724276	4.40815897	1.22532155	3.59755281	0.00032123	0.00224858	Bacteria	Proteobacteri	Alphaproteobac	Sphingomona	Erythrobacteraceae	
4311803	7.97174687	4.39998631	1.42026626	3.098001	0.00194983	0.00937623	Bacteria	Chloroflexi	Anaerolineae	Anaerolineales	Anaerolinaceae	Anaerolinea
1013670	1934.63414	4.37867733	0.70888846	6.17682129	6.54E-10	2.35E-08	Bacteria	Proteobacteri	Gammaproteob	Oceanospirilla	Halomonadaceae	Halomonas
4369890	7.68264208	4.34422994	1.30723071	3.32323125	0.00088981	0.00480138	Bacteria	Proteobacteri	Epsilonproteob	Campylobacte	Helicobacteraceae	Sulfurimonas
274442	12.6814303	4.32367447	1.22662472	3.52485517	0.00042371	0.00285478	Bacteria	Tenericutes	Mollicutes	Acholeplasma	Acholeplasmatacc	Acholeplasma
854050	88.6179344	4.21131723	0.70216989	5.99757589	2.00E-09	6.35E-08	Bacteria	Firmicutes	Bacilli	Bacillales	Planococcaceae	Solibacillus
561804	7.4231352	4.20051971	1.36141942	3.08539724	0.0020328	0.00961124	Bacteria	Proteobacteri	Alphaproteobac	Rhizobiales	Hyphomicrobiace	Parvibaculum
New.Referen	35.6521992	4.19936747	0.7823724	5.36747907	7.98E-08	1.54E-06	Bacteria	Chloroflexi	Anaerolineae	GCA004		
926160	4834.82609	4.18621509	0.77568793	5.39677739	6.78E-08	1.46E-06	Bacteria	Proteobacteri	Alphaproteobac	Rhizobiales	Methylobacteriac	Methylobacterium
1107076	7.25574706	4.13344695	1.33374614	3.0991257	0.00194093	0.00937623	Bacteria	Bacteroidetes	Flavobacteriia	Flavobacterial	NS9	
814277	5.9233094	4.12307089	1.20743954	3.41472243	0.00063847	0.00382373	Bacteria	Proteobacteri	Alphaproteobac	Rhodobacteral	Rhodobacteracea	Paracoccus
4316910	5.40123261	4.0710948	1.19918958	3.39487174	0.00068661	0.00393704	Bacteria	Chloroflexi	Anaerolineae	SHA-20		
New.Referen	13.0091762	4.05317222	1.25249145	3.23608774	0.0012118	0.00634137	Bacteria	Verrucomicro	Verruco-5	WCHB1-41	RFP12	
558264	90.6959621	4.00528881	0.69952188	5.72575197	1.03E-08	2.64E-07	Bacteria	Proteobacteri	Betaproteobact	Burkholderial	Alcaligenaceae	Achromobacter

706555	4.23140793	3.94845479	1.18717313	3.32593004	0.00088124	0.00480138	Archaea	Euryarchaeot	Methanomicrob	Methanosarcir	Methanosarcinace	Methanosarcina
557211	59.9172587	3.92362467	0.87652652	4.4763331	7.59E-06	0.00010232	Bacteria	Cyanobacteri	Synechococcop	Synechococca	Synechococcacea	Synechococcus
534401	25.6921651	3.81413598	1.05954741	3.59977849	0.00031849	0.00224858	Bacteria	Bacteroidetes	Cytophagia	Cytophagales	Cytophagaceae	Leadbetterella
851442	31.3903948	3.47828873	0.90673685	3.83605091	0.00012503	0.00110476	Bacteria	Firmicutes	Clostridia	Halanaerobiale	Halanaerobiaceae	Halanaerobium
New.Referen	7.14139817	3.43741582	1.07145697	3.20816974	0.00133583	0.00685724	Bacteria	Chlorobi	Ignavibacteria	Ignavibacterial	Ignavibacteriaceae	
1084865	87.0834721	3.3772759	0.65027584	5.19360506	2.06E-07	3.83E-06	Bacteria	Firmicutes	Bacilli	Bacillales	Staphylococcacea	Staphylococcus
1065817	415.34997	3.36789194	0.77163657	4.36460903	1.27E-05	0.00015963	Bacteria	Proteobacteri	Gammaproteob	Alteromonada	Colwelliaceae	Colwellia
1083508	1304.07826	3.29529805	0.67239452	4.90084012	9.54E-07	1.66E-05	Bacteria	Proteobacteri	Gammaproteob	Xanthomonad	Xanthomonadace	Stenotrophomonas
996116	5.48463986	3.27314332	1.02892414	3.18113183	0.00146701	0.00738988	Bacteria	Actinobacteri	Actinobacteria	Actinomycetal	Nocardioidaceae	
351345	13.5104121	3.2509422	1.04374157	3.11470031	0.00184132	0.00902246	Bacteria	Proteobacteri	Gammaproteob	Alteromonada	Idiomarinaceae	Pseudidiomarina
984831	57.4257934	3.24576417	0.741742	4.37586677	1.21E-05	0.00015522	Bacteria	Actinobacteri	Actinobacteria	Actinomycetal	Corynebacteriace	Corynebacterium
1101451	20.4988778	3.20165698	0.94073642	3.40335177	0.00066565	0.00389981	Bacteria	Actinobacteri	Actinobacteria	Actinomycetal	Micrococcaceae	Micrococcus
New.Referen	4.41416155	3.11070954	0.94832041	3.28023051	0.00103722	0.00553528	Bacteria	Bacteroidetes	Bacteroidia	Bacteroidales	Rikenellaceae	Blvii28
512148	9.88990213	3.03974077	0.77687854	3.91276191	9.12E-05	0.00084796	Bacteria	Actinobacteri	Thermoleophili	Solirubrobacterales		
579608	41.268171	3.00363	0.70518562	4.25934662	2.05E-05	0.00023494	Bacteria	Firmicutes	Bacilli	Lactobacillales	Streptococcaceae	Streptococcus
278860	8.16471215	2.97163858	0.86603641	3.43130907	0.00060068	0.00372143	Bacteria	Bacteroidetes	Bacteroidia	Bacteroidales	S24-7	
998905	14.8933669	2.93317409	0.88270898	3.32292312	0.00089079	0.00480138	Bacteria	Proteobacteri	Alphaproteobac	Caulobacterale	Caulobacteraceae	Mycoplana
New.Referen	302.414515	2.86571527	0.46369795	6.18013353	6.40E-10	2.35E-08	Bacteria	Planctomycet	Planctomycetia	Pirellulales	Pirellulaceae	
41476	361.373096	2.79618657	0.79597832	3.51289287	0.00044326	0.0029136	Bacteria	Proteobacteri	Gammaproteob	Oceanospirilla	Alcanivoracaceae	Alcanivorax
347439	126.521223	2.6632079	0.72120805	3.69270409	0.00022188	0.00175874	Bacteria	Proteobacteri	Gammaproteob	Alteromonada	Psychromonadac	Psychromonas
688528	11.3885162	2.64865952	0.84067346	3.15064011	0.00162913	0.00805598	Bacteria	Bacteroidetes	Bacteroidia	Bacteroidales	Porphyromonadaceae	
969805	10.5780611	2.40405867	0.74559515	3.22434859	0.0012626	0.00654365	Bacteria	Proteobacteri	Alphaproteobac	Rhizobiales	Rhizobiaceae	Agrobacterium
277566	95.50232	2.40281899	0.73678696	3.2612127	0.00110937	0.00586225	Bacteria	Proteobacteri	Epsilonproteob	Campylobacte	Campylobacterac	Arcobacter
308302	1113.83277	2.14331077	0.50373764	4.25481563	2.09E-05	0.00023494	Bacteria	Planctomycet	Planctomycetia	Planctomyceta	Planctomycetacea	Planctomyces
590601	1166.97446	-2.000949	0.5924094	-3.3776456	0.00073109	0.00414799	Bacteria	Proteobacteri	Betaproteobacte	Burkholderial	Oxalobacteraceae	
1060517	1924.53034	-2.0708131	0.55965126	-3.7001849	0.00021544	0.00173319	Bacteria	Proteobacteri	Betaproteobacte	Burkholderial	Oxalobacteraceae	Janthinobacterium
New.Referen	30.8162903	-2.0790178	0.57088872	-3.6417217	0.00027082	0.00199962	Bacteria	TM7	SC3			
New.Referen	90.0900865	-2.1603251	0.63366485	-3.4092551	0.00065141	0.00385832	Bacteria	OD1	ABY1			
627435	31.4463387	-2.2020075	0.45899914	-4.7974109	1.61E-06	2.71E-05	Bacteria	Proteobacteri	Deltaproteobact	Myxococcales	0319-6G20	
615907	26.2676162	-2.2159818	0.54602503	-4.0583887	4.94E-05	0.00049321	Bacteria	Acidobacteria	Acidobacteriia	Acidobacterial	Koribacteraceae	Candidatus Koribacter
New.Referen	41.110704	-2.2529751	0.51724436	-4.3557268	1.33E-05	0.00016247	Bacteria	Proteobacteri	Alphaproteobac	Rickettsiales	Rickettsiaceae	
210236	29.3832654	-2.3102213	0.60488635	-3.819265	0.00013385	0.00116363	Bacteria	OP3	koll11			
New.Referen	35.4422341	-2.4005018	0.44705682	-5.3695675	7.89E-08	1.54E-06	Bacteria	Proteobacteri	Deltaproteobact	FAC87		
944197	39.0861013	-2.429308	0.51415131	-4.7248893	2.30E-06	3.65E-05	Bacteria	Proteobacteri	Betaproteobacte	Burkholderial	Oxalobacteraceae	Herminiimonas
1136154	9.90350841	-2.5292838	0.71238718	-3.5504342	0.0003846	0.00262402	Bacteria	Acidobacteria	Acidobacteria-5			
1039699	222.737967	-2.6503228	0.64216474	-4.1271696	3.67E-05	0.00038067	Bacteria	Armatimonac	Chthonomonad	Chthonomona	Chthonomonadaceae	
829814	2010.61429	-2.6993434	0.73746308	-3.6603099	0.00025191	0.00191239	Bacteria	Proteobacteri	Alphaproteobac	Rhodobacteral	Rhodobacteraceae	Loktanella
104155	236.175298	-2.8325417	0.89714083	-3.1572988	0.00159238	0.00794716	Bacteria	Proteobacteri	Gammaproteob	Xanthomonad	Sinobacteraceae	Nevskia
New.Referen	8.87779837	-2.8936778	0.66974979	-4.3205356	1.56E-05	0.00018644	Bacteria	Elusimicrobia	Endomicrobia			
New.Referen	31.1050395	-2.95161	0.66520639	-4.4371343	9.12E-06	0.00011985	Bacteria	Chloroflexi	Anaerolineae	SBR1031	oc28	
509913	4341.2136	-2.9543465	0.68768708	-4.2960623	1.74E-05	0.00020372	Bacteria	Proteobacteri	Gammaproteob	Alteromonada	Alteromonadacea	Marinobacter
789831	213.072692	-2.9660397	0.85461132	-3.47063	0.00051924	0.00333178	Bacteria	Proteobacteri	Alphaproteobac	Rhodobacteral	Rhodobacteraceae	Phaeobacter
114081	840.288171	-3.0011051	0.85326901	-3.5171851	0.00043615	0.00290228	Bacteria	Proteobacteri	Gammaproteob	Alteromonada	Alteromonadacea	Glaciecola
New.Referen	74.4192171	-3.1782596	0.79836915	-3.9809399	6.86E-05	0.0006727	Bacteria	Armatimonac	Chthonomonad	SJA-22		
610107	72.6704771	-3.2774572	0.88126201	-3.7190497	0.00019997	0.00163312	Bacteria	Bacteroidetes	Cytophagia	Cytophagales	Cytophagaceae	Flectobacillus
New.Referen	7.35517576	-3.2782845	0.72688841	-4.5100245	6.48E-06	9.35E-05	Bacteria	GN02	GKS2-174			
1082846	99.3678055	-3.3239234	0.91762379	-3.622316	0.00029198	0.0021267	Bacteria	Actinobacteri	Actinobacteria	Actinomycetal	Streptomycetacea	Streptomyces
348517	546.247557	-3.3857539	0.81856402	-4.1362114	3.53E-05	0.00037316	Bacteria	Proteobacteri	Alphaproteobac	Kiloniellales	Kiloniellaceae	Thalassospira
335484	9.06260365	-3.4871619	0.9564129	-3.6460841	0.00026627	0.0019933	Bacteria	Proteobacteri	Betaproteobacte	Rhodocyclales	Rhodocyclaceae	Uliginosibacterium
846667	12.3380916	-3.6919203	1.07745404	-3.4265223	0.00061136	0.0037446	Bacteria	Acidobacteria	Acidobacteriia	Acidobacterial	Acidobacteriaceae	Acidicapsa
New.CleanU	113.70164	-4.0205532	0.98404489	-4.0857417	4.39E-05	0.00044682	Bacteria	Proteobacteri	Alphaproteobac	Rhodobacteral	Rhodobacteraceae	Anaerospora
791738	82.82309	-4.0280144	0.62289101	-6.4666441	1.00E-10	4.91E-09	Bacteria	Proteobacteri	Betaproteobacte	Burkholderial	Comamonadaceae	Polaromonas
566578	123.483142	-5.0314728	0.6541541	-7.6915712	1.45E-14	1.12E-12	Bacteria	Proteobacteri	Betaproteobacte	Burkholderial	Comamonadaceae	Limnobacter
527288	182.966433	-5.6064652	1.02314297	-5.4796498	4.26E-08	9.57E-07	Bacteria	Bacteroidetes	Flavobacteriia	Flavobacterial	Flavobacteriaceae	Muricauda

Table S4. DESeq2 Results Enrichment Day 35 vs Day 0

OTU	baseMean	log2FoldCh	lfcSE	stat	pvalue	padj	Kingdom	Phylum	Class	Order	Family	Genus
674655	29.9045471	9.74148605	1.09051426	8.93292861	4.15E-19	9.33E-17	Bacteria	Proteobacteri	Alphaproteol	Sphingomonada	Sphingomonadaceae	Sphingopyxis
531300	89.6955906	9.06062739	0.98983757	9.15365071	5.50E-20	3.72E-17	Bacteria	Actinobacteri	Actinobacteri	Actinomycetales	Brevibacteriaceae	Brevibacterium
1049387	12.4951745	8.67375437	1.13438439	7.64622157	2.07E-14	1.75E-12	Bacteria	Proteobacteri	Gammaprote	Xanthomonadal	Xanthomonadaceae	Pseudoxanthomonas
573035	99.5947164	7.84163201	0.99738836	7.86216519	3.78E-15	5.10E-13	Bacteria	Firmicutes	Bacilli	Bacillales	Alicyclobacillaceae	Alicyclobacillus
814282	44.6724276	7.80469826	1.23196516	6.33516152	2.37E-10	9.41E-09	Bacteria	Proteobacteri	Alphaproteol	Sphingomonada	Erythrobacteraceae	
926160	4834.82609	7.07338522	0.78684685	8.98953233	2.48E-19	8.38E-17	Bacteria	Proteobacteri	Alphaproteol	Rhizobiales	Methylobacteriaceae	Methylobacterium
534401	25.6921651	6.73597543	1.05776208	6.3681385	1.91E-10	8.07E-09	Bacteria	Bacteroidetes	Cytophagia	Cytophagales	Cytophagaceae	Leadbetterella
1023267	4.63166798	6.71644859	1.99884799	3.36015977	0.00077897	0.0054207	Bacteria	Proteobacteri	Deltaproteob	Myxococcales	Nannocystaceae	Nannocystis
40791	17.5222287	6.14214842	1.57293432	3.90489823	9.43E-05	0.00097891	Bacteria	Verrucomicro	Verrucomicro	Verrucomicrobi	Verrucomicrobiacea	Verrucomicrobium
838940	3.48643647	5.88173085	1.63507282	3.59722868	0.00032163	0.00282962	Bacteria	Actinobacteri	Actinobacteri	Actinomycetales	Microbacteriaceae	
975335	3.20615863	5.83043324	1.73001344	3.37016644	0.00075123	0.00533767	Bacteria	Bacteroidetes	Cytophagia	Cytophagales	Cytophagaceae	Dyadobacter
New.Referen	4.66084471	5.62088206	1.682049	3.34168747	0.00083271	0.00562078	Bacteria	Actinobacteri	Acidimicrobi	Acidimicrobiale	Microthrixaceae	
558264	90.6959621	5.53761905	0.70356731	7.87077362	3.52E-15	5.10E-13	Bacteria	Proteobacteri	Betaproteoba	Burkholderiales	Alcaligenaceae	Achromobacter
826668	7.17286052	5.47875656	1.34356938	4.0777623	4.55E-05	0.00052919	Archaea	Euryarchaeot	Methanomicr	Methanosarcina	Methanosaetaceae	Methanosaeta
223870	22.1848088	5.38306385	0.99336442	5.41902219	5.99E-08	1.35E-06	Bacteria	Bacteroidetes	Cytophagia	Cytophagales	Cytophagaceae	Runella
New.Referen	3.79743714	5.04916458	1.44603951	3.49171965	0.00047992	0.00385651	Bacteria	Cyanobacteri	Chloroplast	Chlorophyta	Trebouxiophyceae	
539735	3170.82046	4.81698783	0.8690321	5.5429343	2.97E-08	7.44E-07	Bacteria	Actinobacteri	Actinobacteri	Actinomycetales	Dietziaceae	Dietzia
358001	5.31878492	4.71219778	1.23133768	3.8268932	0.00012977	0.0013272	Bacteria	Firmicutes	Clostridia	Clostridiales	Ruminococcaceae	Oscillospira
New.Referen	1860.49752	4.70466647	1.03472137	4.54679549	5.45E-06	8.36E-05	Bacteria	Bacteroidetes	Flavobacteriia	Flavobacteriales	Flavobacteriaceae	Zhouia
607117	1.96462918	4.54620049	1.43801037	3.16145182	0.00156985	0.00981155	Bacteria	Bacteroidetes	Flavobacteriia	Flavobacteriales	Flavobacteriaceae	Myroides
269031	3.58074615	4.5331689	1.03020999	4.40023776	1.08E-05	0.00014312	Bacteria	Firmicutes	Clostridia	OPB54		
New.Referen	35.6521992	4.4703399	0.78972103	5.66065701	1.51E-08	4.07E-07	Bacteria	Chloroflexi	Anaerolineae	GCA004		
557211	59.9172587	4.35460765	0.88514563	4.91965105	8.67E-07	1.67E-05	Bacteria	Cyanobacteri	Synechococc	Synechococcale	Synechococcaceae	Synechococcus
2614	31.1700183	4.33974386	1.25180847	3.46677945	0.00052673	0.00413425	Bacteria	Bacteroidetes	Cytophagia	Cytophagales	Cyclobacteriaceae	
1099802	10.0343502	4.32981394	0.96432384	4.48999989	7.12E-06	0.00010229	Bacteria	Actinobacteri	Actinobacteri	Actinomycetales	Propionibacteriaceae	Propionibacterium
1084865	87.0834721	4.18035888	0.65558673	6.37651544	1.81E-10	8.07E-09	Bacteria	Firmicutes	Bacilli	Bacillales	Staphylococcaceae	Staphylococcus
1083508	1304.07826	4.16139465	0.68172396	6.10422235	1.03E-09	3.49E-08	Bacteria	Proteobacteri	Gammaprote	Xanthomonadal	Xanthomonadaceae	Stenotrophomonas
660685	43.325636	4.14293589	0.72012066	5.75311347	8.76E-09	2.57E-07	Bacteria	Actinobacteri	Actinobacteri	Actinomycetales		
771082	3.37342587	4.14171024	1.17569343	3.52278079	0.00042704	0.0035153	Bacteria	Bacteroidetes	Bacteroidia	Bacteroidales	BA008	
571199	26.6629852	4.13741014	1.03602643	3.99353726	6.51E-05	0.00070869	Bacteria	Proteobacteri	Deltaproteob	Myxococcales	OM27	
562576	18.0772408	3.83773613	1.15814753	3.31368503	0.00092075	0.00614665	Bacteria	Proteobacteri	Alphaproteol	Kiloniellales		
992510	243.579151	3.82127151	0.48893613	7.81548203	5.48E-15	6.16E-13	Bacteria	Proteobacteri	Alphaproteol	Sphingomonada	Sphingomonadaceae	Sphingomonas
709480	12.9001872	3.7731172	0.96262705	3.91960438	8.87E-05	0.00093545	Bacteria	Cyanobacteri	Chloroplast	Chlorophyta		
1117129	8.69207232	3.74288482	1.05879536	3.53504083	0.00040771	0.00344007	Bacteria	Chloroflexi	Anaerolineae	Anaerolineales	Anaerolinaceae	
New.Referen	37.1790375	3.61899973	0.67342631	5.37400999	7.70E-08	1.68E-06	Bacteria	Proteobacteri	Gammaprote	Xanthomonadal	Xanthomonadaceae	
811449	136.176384	3.58800317	0.65437426	5.48310556	4.18E-08	1.01E-06	Bacteria	Proteobacteri	Alphaproteol	Caulobacterales	Caulobacteraceae	Caulobacter
New.Referen	4.41416155	3.54315794	0.94176814	3.76224016	0.0001684	0.0016716	Bacteria	Bacteroidetes	Bacteroidia	Bacteroidales	Rikenellaceae	Blvii28
4328135	8.06298311	3.48328074	0.80074012	4.35007646	1.36E-05	0.00017666	Bacteria	Actinobacteri	OPB41			
1081815	16.2634989	3.4624525	0.91407948	3.78791184	0.00015192	0.00153052	Bacteria	Actinobacteri	Actinobacteri	Actinomycetales	Micrococcaceae	Arthrobacter
New.Referen	7.14139817	3.40213288	1.07829135	3.15511469	0.00160435	0.0099352	Bacteria	Chlorobi	Ignavibacteria	Ignavibacteriale	Ignavibacteriaceae	
787709	18.2634084	3.39070658	1.03139682	3.28748987	0.00101085	0.00656079	Bacteria	Actinobacteri	Actinobacteri	Actinomycetales	Actinomycetaceae	Actinomyces
562878	31141.408	3.2243534	0.79932763	4.03383204	5.49E-05	0.00061734	Bacteria	Proteobacteri	Gammaprote	Alteromonadale	Idiomarinaceae	Idiomarina
810672	57.4359786	3.14257084	0.89009925	3.53058474	0.00041464	0.00345535	Bacteria	Actinobacteri	Actinobacteri	Actinomycetales	Microbacteriaceae	Candidatus Rhodolur
514711	194.657852	3.09929947	0.44177334	7.01558731	2.29E-12	1.41E-10	Bacteria	Actinobacteri	Actinobacteri	Actinomycetales	Mycobacteriaceae	Mycobacterium
579608	41.268171	3.05014477	0.70804719	4.30782693	1.65E-05	0.00020234	Bacteria	Firmicutes	Bacilli	Lactobacillales	Streptococcaceae	Streptococcus
1002005	4.74500795	3.0414871	0.88001447	3.45617852	0.00054789	0.00418902	Bacteria	Actinobacteri	Actinobacteri	Actinomycetales	Micrococcaceae	Kocuria
1053775	41.1577579	3.02739325	0.6856853	4.41513514	1.01E-05	0.00013628	Bacteria	Proteobacteri	Alphaproteol	Rhizobiales	Hyphomicrobiaceae	Devosia
969805	10.5780611	2.98433154	0.73317306	4.07043265	4.69E-05	0.00053686	Bacteria	Proteobacteri	Alphaproteol	Rhizobiales	Rhizobiaceae	Agrobacterium
818854	125.490938	2.95997136	0.86479207	3.42275498	0.0006199	0.00464924	Bacteria	Proteobacteri	Alphaproteol	Rhodobacterales	Rhodobacteraceae	Rhodobacter

New.Referen	302.414515	2.87731587	0.46905035	6.13434328	8.55E-10	3.21E-08	Bacteria	Planctomycet	Planctomycet	Pirellulales	Pirellulaceae	
1108830	20.4619526	2.65885885	0.55824583	4.76288172	1.91E-06	3.48E-05	Bacteria	Proteobacteri	Alphaproteob	Rhizobiales	Beijerinckiaceae	Beijerinckia
984831	57.4257934	2.59323361	0.75038641	3.45586428	0.00054853	0.00418902	Bacteria	Actinobacteri	Actinobacteri	Actinomycetales	Corynebacteriaceae	Corynebacterium
4448102	26.3362792	2.43161944	0.65092648	3.73562838	0.00018725	0.00183176	Bacteria	Actinobacteri	Thermoleoph	Gaiellales		
697479	96.0298736	2.30234129	0.66657173	3.45400379	0.00055233	0.00418902	Bacteria	Proteobacteri	Betaproteoba	Burkholderiales	Comamonadaceae	Hydrogenophaga
1081222	10.1801815	2.18741724	0.6445123	3.39391075	0.00068902	0.00499098	Bacteria	Firmicutes	Bacilli	Bacillales	Paenibacillaceae	Paenibacillus
177302	445.247638	2.17757914	0.63711581	3.41787021	0.00063113	0.00468147	Bacteria	Chloroflexi	Ktedonobacte	Thermogemmat	Thermogemmatisporaceae	
New.Referen	5.53982114	2.09224651	0.64160141	3.26097555	0.0011103	0.00713762	Bacteria	Chlamydiae	Chlamydiia	Chlamydiales	Parachlamydiaceae	
518927	6.80886175	2.08446047	0.62333145	3.34406432	0.00082561	0.00562078	Archaea	Crenarchaeot	MCG	pGrfC26		
694558	1961.75205	2.07861336	0.6180734	3.36305262	0.00077086	0.00542009	Bacteria	Firmicutes	Bacilli	Bacillales	Bacillaceae	Bacillus
758197	276.595593	-2.0439917	0.42114946	-4.8533641	1.21E-06	2.28E-05	Bacteria	Proteobacteri	Deltaproteob	MIZ46		
357721	144.291421	-2.1003065	0.46432266	-4.5233772	6.09E-06	9.13E-05	Bacteria	Elusimicrobia	Elusimicrobia	FAC88		
770614	89.912115	-2.120875	0.5873472	-3.6109391	0.00030509	0.00282017	Bacteria	Proteobacteri	Deltaproteob	Spirobacillales		
279180	85.774391	-2.1677028	0.40823905	-5.3098859	1.10E-07	2.31E-06	Bacteria	Proteobacteri	Gammaprote	Legionellales		
New.Referen	90.0900865	-2.2422986	0.63717313	-3.5191355	0.00043296	0.00352102	Bacteria	OD1	ABY1			
New.Referen	41.110704	-2.2848482	0.51510674	-4.435679	9.18E-06	0.00012644	Bacteria	Proteobacteri	Alphaproteob	Rickettsiales	Rickettsiaceae	
New.Referen	8.87779837	-2.2883105	0.64543547	-3.5453745	0.00039206	0.00334984	Bacteria	Elusimicrobia	Endomicrobia			
656621	85.5884189	-2.4020202	0.52595293	-4.5669871	4.95E-06	7.77E-05	Archaea	Crenarchaeot	Thaumarchae	Cenarchaeales	SAGMA-X	
New.Referen	7.35517576	-2.4224827	0.69638425	-3.4786581	0.00050393	0.00400181	Bacteria	GN02	GKS2-174			
646549	313.436754	-2.4283206	0.5464616	-4.4437169	8.84E-06	0.00012434	Bacteria	Proteobacteri	Gammaprote	Pseudomonadal	Pseudomonadaceae	Pseudomonas
4310474	5.93962064	-2.4980259	0.67246239	-3.7147445	0.00020341	0.00196145	Bacteria	Nitrospirae	Nitrospira	Nitrospirales	Nitrospiraceae	JG37-AG-70
539880	17.2102506	-2.5491397	0.59146899	-4.3098451	1.63E-05	0.00020234	Bacteria	Proteobacteri	Gammaproteobacteria			
330280	25.6960735	-2.6223565	0.56455021	-4.6450368	3.40E-06	5.74E-05	Bacteria	Elusimicrobia	Elusimicrobia	IIb		
New.Referen	35.4422341	-2.6450142	0.44416841	-5.9549803	2.60E-09	7.98E-08	Bacteria	Proteobacteri	Deltaproteob	FAC87		
New.Referen	31.1050395	-2.6953939	0.65638695	-4.10641	4.02E-05	0.00047588	Bacteria	Chloroflexi	Anaerolineae	SBR1031	oc28	
627435	31.4463387	-2.7508782	0.45977866	-5.9830488	2.19E-09	7.04E-08	Bacteria	Proteobacteri	Deltaproteob	Myxococcales	0319-6G20	
944197	39.0861013	-2.7952932	0.51471897	-5.4307172	5.61E-08	1.31E-06	Bacteria	Proteobacteri	Betaproteoba	Burkholderiales	Oxalobacteraceae	Herminiimonas
1136154	9.90350841	-2.8059458	0.70963986	-3.954042	7.68E-05	0.00082331	Bacteria	Acidobacteria	Acidobacteria-5			
1039699	222.737967	-2.9598516	0.64564945	-4.5843013	4.56E-06	7.32E-05	Bacteria	Armatimonad	Chthonomon	Chthonomonada	Chthonomonadaceae	
830290	7086.73229	-2.9604732	0.64575557	-4.5845105	4.55E-06	7.32E-05	Bacteria	Proteobacteri	Gammaprote	Vibrionales	Pseudoalteromonada	Pseudoalteromonas
210236	29.3832654	-3.2092655	0.6115118	-5.2480844	1.54E-07	3.14E-06	Bacteria	OP3	koll11			
610107	72.6704771	-3.275721	0.88807337	-3.6885702	0.00022552	0.00211423	Bacteria	Bacteroidetes	Cytophagia	Cytophagales	Cytophagaceae	Flectobacillus
225453	154.172374	-3.2914862	0.57873366	-5.6873939	1.29E-08	3.63E-07	Bacteria	Proteobacteri	Gammaprote	Alteromonadale	Alteromonadaceae	Cellvibrio
New.CleanU	113.70164	-3.3144289	0.99015624	-3.3473797	0.00081579	0.00561899	Bacteria	Proteobacteri	Alphaproteob	Rhodobacterales	Rhodobacteraceae	Anaerospora
1060517	1924.53034	-3.4771389	0.56777605	-6.124138	9.12E-10	3.24E-08	Bacteria	Proteobacteri	Betaproteoba	Burkholderiales	Oxalobacteraceae	Janthinobacterium
791738	82.82309	-3.4860807	0.62011745	-5.6216458	1.89E-08	4.91E-07	Bacteria	Proteobacteri	Betaproteoba	Burkholderiales	Comamonadaceae	Polaromonas
335484	9.06260365	-3.528616	0.95387459	-3.6992452	0.00021624	0.00205582	Bacteria	Proteobacteri	Betaproteoba	Rhodocyclales	Rhodocyclaceae	Uliginosibacterium
809945	9.96491164	-3.6157455	1.09234895	-3.3100645	0.00093274	0.00614665	Bacteria	Proteobacteri	Betaproteoba	Methylophilales		
1082846	99.3678055	-3.7247844	0.92494363	-4.0270393	5.65E-05	0.00062502	Bacteria	Actinobacteri	Actinobacteri	Actinomycetales	Streptomycetaceae	Streptomyces
New.Referen	3.24759572	-3.9318774	0.83860898	-4.6885705	2.75E-06	4.76E-05	Bacteria	Proteobacteri	Deltaproteob	Bdellovibrional	Bdellovibrionaceae	
8882	234.988934	-4.0273336	0.95208259	-4.2300254	2.34E-05	0.00028165	Bacteria	Proteobacteri	Gammaprote	Alteromonadale	Alteromonadaceae	Alteromonas
141815	11.9266717	-4.1309961	1.14622412	-3.6040038	0.00031335	0.00282017	Bacteria	Armatimonad	Armatimonad	Armatimonadal	Armatimonadaceae	Armatimonas
2398623	2.89927389	-4.1559898	1.31213758	-3.167343	0.00153839	0.00970478	Bacteria	Firmicutes	Clostridia	Clostridiales	Peptostreptococcacea	Clostridium
New.Referen	7.14428032	-4.2622929	0.98803075	-4.3139274	1.60E-05	0.00020234	Bacteria	GN02	3BR-5F			
789831	213.072692	-4.5328387	0.86716834	-5.2271727	1.72E-07	3.42E-06	Bacteria	Proteobacteri	Alphaproteob	Rhodobacterales	Rhodobacteraceae	Phaeobacter
566578	123.483142	-4.8216029	0.65278512	-7.3862022	1.51E-13	1.13E-11	Bacteria	Proteobacteri	Betaproteoba	Burkholderiales	Comamonadaceae	Limnobacter
829814	2010.61429	-4.872419	0.74844087	-6.5100921	7.51E-11	3.90E-09	Bacteria	Proteobacteri	Alphaproteob	Rhodobacterales	Rhodobacteraceae	Loktanella
509913	4341.2136	-5.3992131	0.69792817	-7.7360585	1.03E-14	9.89E-13	Bacteria	Proteobacteri	Gammaprote	Alteromonadale	Alteromonadaceae	Marinobacter
114081	840.288171	-5.6203881	0.8667956	-6.4840985	8.93E-11	4.30E-09	Bacteria	Proteobacteri	Gammaprote	Alteromonadale	Alteromonadaceae	Glaciecola
348517	546.247557	-5.725704	0.83189269	-6.8827436	5.87E-12	3.30E-10	Bacteria	Proteobacteri	Alphaproteob	Kiloniellales	Kiloniellaceae	Thalassospira
527288	182.966433	-7.4300138	1.04445174	-7.1137933	1.13E-12	7.62E-11	Bacteria	Bacteroidetes	Flavobacteriia	Flavobacteriales	Flavobacteriaceae	Muricauda

Table S5. DESeq2 Results Enrichement Day 49 vs Day 0

OTU	baseMean	log2FoldCh	lfcSE	stat	pvalue	padj	Kingdom	Phylum	Class	Order	Family	Genus
674655	29.9045471	7.75167889	1.09421189	7.08425761	1.40E-12	9.41E-11	Bacteria	Proteobacteri	Alphaproteol	Sphingomona	Sphingomonadacea	Sphingopyxis
1049387	12.4951745	6.56872494	1.14238551	5.75000724	8.92E-09	2.41E-07	Bacteria	Proteobacteri	Gammaprote	Xanthomona	Xanthomonadaceae	Pseudoxanthomonas
1099802	10.0343502	6.400382	0.95869045	6.67617162	2.45E-11	1.15E-09	Bacteria	Actinobacteri	Actinobacteri	Actinomyceta	Propionibacteriacea	Propionibacterium
531300	89.6955906	6.27963262	0.99245522	6.32737127	2.49E-10	9.23E-09	Bacteria	Actinobacteri	Actinobacteri	Actinomyceta	Brevibacteriaceae	Brevibacterium
558264	90.6959621	6.14079429	0.70358917	8.7278124	2.60E-18	4.56E-16	Bacteria	Proteobacteri	Betaproteoba	Burkholderia	Alcaligenaceae	Achromobacter
575028	3.42428937	5.8114056	1.42847792	4.06825021	4.74E-05	0.00055499	Bacteria	Proteobacteri	Betaproteoba	Burkholderia	Alcaligenaceae	Alcaligenes
573035	99.5947164	5.74875195	0.99968267	5.75057681	8.89E-09	2.41E-07	Bacteria	Firmicutes	Bacilli	Bacillales	Alicyclobacillaceae	Alicyclobacillus
1083508	1304.07826	5.74105389	0.681677	8.4219563	3.70E-17	5.21E-15	Bacteria	Proteobacteri	Gammaprote	Xanthomona	Xanthomonadaceae	Stenotrophomonas
40791	17.5222287	5.63630435	1.57636925	3.57549752	0.00034956	0.00336633	Bacteria	Verrucomicro	Verrucomicro	Verrucomicro	Verrucomicrobiacea	Verrucomicrobium
585419	12.6025604	5.61028928	1.31675922	4.2606797	2.04E-05	0.0002924	Bacteria	Firmicutes	Clostridia	Clostridiales	Veillonellaceae	Veillonella
814282	44.6724276	5.5519407	1.23447779	4.49740023	6.88E-06	0.00011246	Bacteria	Proteobacteri	Alphaproteol	Sphingomona	Erythrobacteraceae	
787709	18.2634084	5.45923715	1.02733792	5.31396441	1.07E-07	2.60E-06	Bacteria	Actinobacteri	Actinobacteri	Actinomyceta	Actinomycetaceae	Actinomyces
534401	25.6921651	5.4479523	1.06080698	5.13566782	2.81E-07	6.34E-06	Bacteria	Bacteroidetes	Cytophagia	Cytophagales	Cytophagaceae	Leadbetterella
814277	5.9233094	4.97536246	1.21205264	4.10490626	4.04E-05	0.00049886	Bacteria	Proteobacteri	Alphaproteol	Rhodobactera	Rhodobacteraceae	Paracoccus
579608	41.268171	4.87921403	0.705611	6.91487807	4.68E-12	2.53E-10	Bacteria	Firmicutes	Bacilli	Lactobacillale	Streptococcaceae	Streptococcus
926160	4834.82609	4.86302353	0.78688814	6.18006967	6.41E-10	2.14E-08	Bacteria	Proteobacteri	Alphaproteol	Rhizobiales	Methylobacteriacea	Methylobacterium
990864	9.14215216	4.69982422	1.10700386	4.24553554	2.18E-05	0.00030661	Bacteria	Proteobacteri	Gammaprote	Pseudomona	Moraxellaceae	Enhydrobacter
969805	10.5780611	4.62332455	0.72587816	6.36928453	1.90E-10	7.42E-09	Bacteria	Proteobacteri	Alphaproteol	Rhizobiales	Rhizobiaceae	Agrobacterium
133075	5.79004777	4.55159283	1.18793098	3.83152968	0.00012735	0.00137733	Bacteria	Firmicutes	Bacilli	Lactobacillale	Lactobacillaceae	Lactobacillus
1024520	8.19701342	4.54740353	1.31892001	3.44782359	0.00056512	0.00461955	Bacteria	Proteobacteri	Betaproteoba	Burkholderia	Comamonadaceae	Comamonas
1084865	87.0834721	4.35302522	0.65596944	6.63601835	3.22E-11	1.42E-09	Bacteria	Firmicutes	Bacilli	Bacillales	Staphylococcaceae	Staphylococcus
351294	84.1030873	4.241091	1.19339827	3.5537935	0.00037972	0.00353503	Archaea	Euryarchaeot	Thermoplasn	E2	Marine group II	
New.Referen	302.414515	4.09409138	0.46882551	8.73265491	2.49E-18	4.56E-16	Bacteria	Planctomycet	Planctomycet	Pirellulales	Pirellulaceae	
223870	22.1848088	4.08374371	0.99694936	4.09623987	4.20E-05	0.00050897	Bacteria	Bacteroidetes	Cytophagia	Cytophagales	Cytophagaceae	Runella
269031	3.58074615	3.63807721	1.04877178	3.46889313	0.00052261	0.00437373	Bacteria	Firmicutes	Clostridia	OPB54		
663885	8.66740306	3.45863757	0.97683303	3.54066403	0.00039912	0.00359721	Bacteria	Bacteroidetes	Bacteroidia	Bacteroidales	Prevotellaceae	Prevotella
1117129	8.69207232	3.40481335	1.06310042	3.20272034	0.00136136	0.00886523	Bacteria	Chloroflexi	Anaerolineae	Anaerolineale	Anaerolinaceae	
New.Referen	11.649776	3.3800009	1.00261573	3.37118278	0.00074846	0.00571922	Bacteria	Planctomycet	Planctomycet	Pirellulales	Pirellulaceae	Pirellula
1101451	20.4988778	3.36445105	0.94717167	3.55210269	0.00038217	0.00353503	Bacteria	Actinobacteri	Actinobacteri	Actinomyceta	Micrococcaceae	Micrococcus
984831	57.4257934	3.36400396	0.75018394	4.48423882	7.32E-06	0.00011691	Bacteria	Actinobacteri	Actinobacteri	Actinomyceta	Corynebacteriaceae	Corynebacterium
539735	3170.82046	3.3186831	0.87146494	3.80816596	0.00014	0.00149123	Bacteria	Actinobacteri	Actinobacteri	Actinomyceta	Dietziaceae	Dietzia
557211	59.9172587	3.19673335	0.88831827	3.59863516	0.00031989	0.00316738	Bacteria	Cyanobacteri	Synechococc	Synechococc	Synechococcaceae	Synechococcus
709480	12.9001872	3.09539676	0.9666124	3.20231434	0.00136328	0.00886523	Bacteria	Cyanobacteri	Chloroplast	Chlorophyta		
177302	445.247638	3.02061708	0.63706111	4.74148718	2.12E-06	3.73E-05	Bacteria	Chloroflexi	Ktedonobact	Thermogemn	Thermogemmatisporaceae	
1081815	16.2634989	3.00421033	0.9177498	3.27345245	0.00106242	0.00750349	Bacteria	Actinobacteri	Actinobacteri	Actinomyceta	Micrococcaceae	Arthrobacter
308302	1113.83277	2.96464955	0.5104817	5.80755305	6.34E-09	1.86E-07	Bacteria	Planctomycet	Planctomycet	Planctomycet	Planctomycetaceae	Planctomyces
New.Referen	35.6521992	2.95890818	0.79460174	3.72376255	0.00019628	0.00202914	Bacteria	Chloroflexi	Anaerolineae	GCA004		
New.Referen	12.8060345	2.92206681	0.84602655	3.45387128	0.0005526	0.00457034	Bacteria	Chloroflexi	Ktedonobact	JG30-KF-AS9		
1002005	4.74500795	2.86536223	0.88710851	3.23000196	0.00123789	0.00836769	Bacteria	Actinobacteri	Actinobacteri	Actinomyceta	Micrococcaceae	Kocuria
1053775	41.1577579	2.85320511	0.68713955	4.15229355	3.29E-05	0.00042852	Bacteria	Proteobacteri	Alphaproteol	Rhizobiales	Hyphomicrobiaceae	Devosia
4328135	8.06298311	2.81278721	0.80931079	3.47553404	0.00050984	0.00431826	Bacteria	Actinobacteri	OPB41			
769222	91.2152448	2.68499697	0.58890563	4.55929922	5.13E-06	8.59E-05	Bacteria	Cyanobacteri	Chloroplast	Streptophyta		
547579	5.74030174	2.61825586	0.60134729	4.35398299	1.34E-05	0.00019579	Bacteria	Chlamydiae	Chlamydiia	Chlamydiales	Parachlamydiaceae	Candidatus Protochlamydia
660685	43.325636	2.51790364	0.72373512	3.47904028	0.00050321	0.00431826	Bacteria	Actinobacteri	Actinobacteri	Actinomycetales		
992510	243.579151	2.38868358	0.48972351	4.87761672	1.07E-06	2.04E-05	Bacteria	Proteobacteri	Alphaproteol	Sphingomona	Sphingomonadacea	Sphingomonas
New.Referen	115.441108	2.35168929	0.73455032	3.20153598	0.00136697	0.00886523	Bacteria	Planctomycet	Phycisphaera	Phycisphaerales		
514711	194.657852	2.29484576	0.44270637	5.18367453	2.18E-07	5.10E-06	Bacteria	Actinobacteri	Actinobacteri	Actinomyceta	Mycobacteriaceae	Mycobacterium

1013670	1934.63414	2.27121798	0.71930573	3.15751411	0.00159121	0.00989927	Bacteria	Proteobacteri	Gammaprote	Oceanospirill	Halomonadaceae	Halomonas
New.Referen	5.53982114	2.26242156	0.64853357	3.48851879	0.0004857	0.00426813	Bacteria	Chlamydiae	Chlamydiia	Chlamydiales	Parachlamydiaceae	
New.Referen	37.1790375	2.21673405	0.6774562	3.27214371	0.00106735	0.00750349	Bacteria	Proteobacteri	Gammaprote	Xanthomona	Xanthomonadaceae	
811263	66.1679899	2.16780991	0.52070368	4.16323137	3.14E-05	0.0004162	Bacteria	Actinobacteri	Thermoleoph	Gaiellales	Gaiellaceae	
314567	365.566861	2.15699062	0.51210255	4.21202866	2.53E-05	0.00034886	Bacteria	Planctomycet	Planctomycet	Gemmatales	Gemmataceae	Gemmata
811449	136.176384	2.0993587	0.65606144	3.1999422	0.00137455	0.00886523	Bacteria	Proteobacteri	Alphaproteol	Caulobactera	Caulobacteraceae	Caulobacter
1081222	10.1801815	2.06306356	0.65151811	3.16654829	0.0015426	0.00968255	Bacteria	Firmicutes	Bacilli	Bacillales	Paenibacillaceae	Paenibacillus
141636	26.4608815	-2.0596316	0.6246477	-3.2972692	0.0009763	0.00720236	Bacteria	Proteobacteri	Deltaproteob	Bdellovibrior	Bacteriovoracaceae	
627435	31.4463387	-2.1986548	0.46040225	-4.7755083	1.79E-06	3.23E-05	Bacteria	Proteobacteri	Deltaproteob	Myxococcale	0319-6G20	
4310474	5.93962064	-2.2099951	0.68215611	-3.2397205	0.00119647	0.0082417	Bacteria	Nitrospirae	Nitrospira	Nitrospirales	Nitrospiraceae	JG37-AG-70
New.Referen	41.110704	-2.2996002	0.5199285	-4.4229161	9.74E-06	0.00015213	Bacteria	Proteobacteri	Alphaproteol	Rickettsiales	Rickettsiaceae	
330280	25.6960735	-2.3157312	0.56891238	-4.0704532	4.69E-05	0.00055499	Bacteria	Elusimicrobia	Elusimicrobia	IIb		
87245	22.7263105	-2.3404792	0.67292308	-3.4780783	0.00050502	0.00431826	Bacteria	Proteobacteri	Deltaproteob	Desulfovibrio	Desulfovibrionacea	Desulfovibrio
New.Referen	31.8923655	-2.4205803	0.73457899	-3.2951941	0.00098354	0.00720236	Bacteria	Armatimonac	Armatimonac	Armatimonac	Armatimonadaceae	
New.Referen	5.18919688	-2.5238893	0.74803108	-3.3740433	0.00074073	0.00571922	Bacteria	TM7	TM7-1			
590601	1166.97446	-2.6423147	0.60093295	-4.3970208	1.10E-05	0.00016772	Bacteria	Proteobacteri	Betaproteoba	Burkholderia	Oxalobacteraceae	
539880	17.2102506	-2.6480065	0.60379348	-4.3856162	1.16E-05	0.00017299	Bacteria	Proteobacteri	Gammaproteobacteria			
New.Referen	31.1050395	-2.6757512	0.66457974	-4.0262305	5.67E-05	0.00065319	Bacteria	Chloroflexi	Anaerolineae	SBR1031	oc28	
4483076	2.64003403	-2.6872609	0.81714358	-3.2886031	0.00100686	0.00729713	Bacteria	Proteobacteria				
New.Referen	7.35517576	-2.692152	0.71744694	-3.7524058	0.00017515	0.00183772	Bacteria	GN02	GKS2-174			
New.Referen	8.87779837	-2.7592858	0.67053948	-4.1150236	3.87E-05	0.000486	Bacteria	Elusimicrobia	Endomicrobia			
712582	11.3547737	-2.9932465	0.92467059	-3.237095	0.00120753	0.0082417	Bacteria	Fibrobacteres	Fibrobacteria	258ds10		
335484	9.06260365	-3.0460827	0.95596153	-3.1864072	0.00144052	0.00914526	Bacteria	Proteobacteri	Betaproteoba	Rhodocyclal	Rhodocyclaceae	Uliginosibacterium
New.Referen	3.24759572	-3.0637566	0.83275766	-3.6790495	0.0002341	0.00235108	Bacteria	Proteobacteri	Deltaproteob	Bdellovibrior	Bdellovibrionaceae	
New.Referen	7.14428032	-3.0941969	0.97127357	-3.185711	0.00144399	0.00914526	Bacteria	GN02	3BR-5F			
944197	39.0861013	-3.097329	0.52271922	-5.9254164	3.12E-09	9.52E-08	Bacteria	Proteobacteri	Betaproteoba	Burkholderia	Oxalobacteraceae	Herminiimonas
934235	6.61483979	-3.1025296	0.94468687	-3.2841883	0.00102277	0.00733678	Bacteria	Proteobacteri	Alphaproteol	Rhizobiales	Methylocystaceae	Methylosinus
100640	51.1756254	-3.1687678	0.89865102	-3.5261384	0.00042167	0.0037523	Bacteria	Proteobacteri	Betaproteoba	IS-44		
4347035	9.45681114	-3.1897666	0.95183821	-3.3511647	0.00080472	0.00608302	Bacteria	Acidobacteria	EC1113			
220050	6.37478775	-3.2397625	0.91198668	-3.5524231	0.0003817	0.00353503	Bacteria	Elusimicrobia	Elusimicrobia	MVP-88		
161298	30.6750244	-3.5844541	1.06189987	-3.3755104	0.00073679	0.00571922	Bacteria	Proteobacteri	Gammaprote	Oceanospirill	Oceanospirillaceae	
1060517	1924.53034	-3.6410695	0.56800922	-6.4102297	1.45E-10	6.01E-09	Bacteria	Proteobacteri	Betaproteoba	Burkholderia	Oxalobacteraceae	Janthinobacterium
1136154	9.90350841	-3.8633943	0.75852812	-5.0932777	3.52E-07	7.50E-06	Bacteria	Acidobacteria	Acidobacteria-5			
830290	7086.73229	-3.9838445	0.6459362	-6.1675511	6.94E-10	2.22E-08	Bacteria	Proteobacteri	Gammaprote	Vibrionales	Pseudoalteromonad	Pseudoalteromonas
791738	82.82309	-4.4119874	0.63013016	-7.0017079	2.53E-12	1.48E-10	Bacteria	Proteobacteri	Betaproteoba	Burkholderia	Comamonadaceae	Polaromonas
610107	72.6704771	-4.427899	0.89670282	-4.9379782	7.89E-07	1.54E-05	Bacteria	Bacteroidetes	Cytophagia	Cytophagales	Cytophagaceae	Flectobacillus
566578	123.483142	-4.6780097	0.65568485	-7.1345399	9.71E-13	7.59E-11	Bacteria	Proteobacteri	Betaproteoba	Burkholderia	Comamonadaceae	Limnobacter
646909	584.182097	-5.4952699	1.01992396	-5.3879212	7.13E-08	1.79E-06	Bacteria	Proteobacteri	Gammaprote	Alteromonad	Alteromonadaceae	ZD0117
New.CleanU	113.70164	-5.7561601	1.00758253	-5.7128423	1.11E-08	2.89E-07	Bacteria	Proteobacteri	Alphaproteol	Rhodobactera	Rhodobacteraceae	Anaerospora
556648	106.888491	-5.7570993	1.48325657	-3.8813914	0.00010386	0.00115895	Bacteria	Acidobacteria	[Chloracidob	PK29		
829814	2010.61429	-5.8168574	0.74972699	-7.7586341	8.58E-15	7.54E-13	Bacteria	Proteobacteri	Alphaproteol	Rhodobactera	Rhodobacteraceae	Loktanella
141815	11.9266717	-5.9464605	1.19929646	-4.9582908	7.11E-07	1.43E-05	Bacteria	Armatimonac	Armatimonac	Armatimonac	Armatimonadaceae	Armatimonas
8882	234.988934	-6.0082507	0.96721353	-6.2119175	5.23E-10	1.84E-08	Bacteria	Proteobacteri	Gammaprote	Alteromonad	Alteromonadaceae	Alteromonas
789831	213.072692	-6.0561527	0.87948116	-6.8860517	5.74E-12	2.88E-10	Bacteria	Proteobacteri	Alphaproteol	Rhodobactera	Rhodobacteraceae	Phaeobacter
509913	4341.2136	-6.098498	0.69862538	-8.7292821	2.56E-18	4.56E-16	Bacteria	Proteobacteri	Gammaprote	Alteromonad	Alteromonadaceae	Marinobacter
609349	56.993721	-6.2842614	1.22483951	-5.1306815	2.89E-07	6.34E-06	Bacteria	Proteobacteri	Alphaproteol	Rhodobactera	Hyphomonadaceae	Hyphomonas
348517	546.247557	-6.7139086	0.83857546	-8.0063261	1.18E-15	1.19E-13	Bacteria	Proteobacteri	Alphaproteol	Kiloniellales	Kiloniellaceae	Thalassospira
1082846	99.3678055	-6.9998894	0.98910284	-7.0770087	1.47E-12	9.41E-11	Bacteria	Actinobacteri	Actinobacteri	Actinomyceta	Streptomycetaceae	Streptomyces
114081	840.288171	-7.046999	0.87325647	-8.069793	7.04E-16	8.25E-14	Bacteria	Proteobacteri	Gammaprote	Alteromonad	Alteromonadaceae	Glaciecola
527288	182.966433	-10.885754	1.13537286	-9.5878226	9.00E-22	6.32E-19	Bacteria	Bacteroidetes	Flavobacteria	Flavobacteria	Flavobacteriaceae	Muricauda

411

Table S6. DESeq2 Results Enrichment Day 56 vs Day 0

OTU	baseMean	log2FoldCh	lfcSE	stat	pvalue	padj	Kingdom	Phylum	Class	Order	Family	Genus
New.Referen	1860.49752	10.0941272	0.89980755	11.2180956	3.32E-29	4.31E-27	Bacteria	Bacteroidetes	Flavobacterii	Flavobacteria	Flavobacteriaceae	Zhouia
1501738	98.2772819	6.72501225	1.05423414	6.37904997	1.78E-10	4.44E-09	Bacteria	Proteobacteri	Betaproteoba	Rhodocyclale	Rhodocyclaceae	
531300	89.6955906	5.59267434	0.875097	6.39091936	1.65E-10	4.44E-09	Bacteria	Actinobacteri	Actinobacteri	Actinomyceta	Brevibacteriaceae	Brevibacterium
40791	17.5222287	5.23535164	1.37797998	3.79929443	0.00014511	0.00116087	Bacteria	Verrucomicro	Verrucomicro	Verrucomicro	Verrucomicrobiacea	Verrucomicrobium
556648	106.888491	5.06393153	1.21323609	4.17390447	2.99E-05	0.00030798	Bacteria	Acidobacteria	[Chloracidob	PK29		
104155	236.175298	5.04033699	0.74608382	6.75572486	1.42E-11	4.39E-10	Bacteria	Proteobacteri	Gammaprote	Xanthomona	Sinobacteraceae	Nevskia
607117	1.96462918	4.61255353	1.25591113	3.67267511	0.00024002	0.00172818	Bacteria	Bacteroidetes	Flavobacterii	Flavobacteria	Flavobacteriaceae	Myroides
674655	29.9045471	4.4629188	0.97942668	4.55666454	5.20E-06	6.01E-05	Bacteria	Proteobacteri	Alphaproteol	Sphingomona	Sphingomonadaceae	Sphingopyxis
833317	4.573678	4.32946279	1.06425795	4.06805775	4.74E-05	0.00043267	Bacteria	Firmicutes	Bacilli	Bacillales	Planococcaceae	Sporosarcina
575028	3.42428937	4.16554368	1.25465621	3.32006779	0.00089996	0.0049006	Bacteria	Proteobacteri	Betaproteoba	Burkholderia	Alcaligenaceae	Alcaligenes
New.Referen	115.441108	3.97432098	0.63626008	6.246378	4.20E-10	9.72E-09	Bacteria	Planctomycet	Phycisphaera	Phycisphaerales		
926160	4834.82609	3.90822604	0.68195523	5.73091289	9.99E-09	1.96E-07	Bacteria	Proteobacteri	Alphaproteol	Rhizobiales	Methylobacteriaceae	Methylobacterium
308302	1113.83277	3.77507184	0.44239262	8.53330658	1.42E-17	7.09E-16	Bacteria	Planctomycet	Planctomycet	Planctomycet	Planctomycetaceae	Planctomyces
1049387	12.4951745	3.75963426	1.01941761	3.68802169	0.000226	0.00165454	Bacteria	Proteobacteri	Gammaprote	Xanthomona	Xanthomonadaceae	Pseudoxanthomonas
547362	11.8169151	3.62385388	1.01233201	3.57970889	0.00034398	0.00225149	Bacteria	Proteobacteri	Betaproteoba	Hydrogenopl	Hydrogenophilaceae	Thiobacillus
990864	9.14215216	3.54898386	0.97175349	3.65214419	0.00026006	0.00183173	Bacteria	Proteobacteri	Gammaprote	Pseudomona	Moraxellaceae	Enhydrobacter
538315	3.89711881	3.53489397	1.11627194	3.16669606	0.00154181	0.0075689	Bacteria	Actinobacteri	Actinobacteri	Actinomyceta	Pseudonocardiaceae	Pseudonocardia
573035	99.5947164	3.51777083	0.87891163	4.00241699	6.27E-05	0.00055656	Bacteria	Firmicutes	Bacilli	Bacillales	Alicyclobacillaceae	Alicyclobacillus
New.Referen	32.9155185	3.49292281	0.89714039	3.89339601	9.89E-05	0.00080069	Bacteria	Planctomycet	OM190	CL500-15		
4614	13.5143429	3.48602871	0.92796875	3.75662295	0.00017222	0.00131294	Bacteria	Proteobacteri	Alphaproteol	Rhizobiales	Hyphomicrobiaceae	Pedomicrobium
580625	537.994215	3.43953562	0.37519936	9.16722155	4.85E-20	3.49E-18	Bacteria	Proteobacteri	Alphaproteol	Rhizobiales	Bradyrhizobiaceae	Bradyrhizobium
720511	15.3778315	3.41876706	1.09829283	3.11280104	0.00185321	0.00896179	Archaea	Crenarchaeot	Thaumarchae	Nitrososphae	Nitrososphaeraceae	Candidatus Nitrososphaera
New.Referen	30.9400002	3.38867791	0.69363912	4.8853616	1.03E-06	1.24E-05	Bacteria	Chloroflexi	TK17			
534401	25.6921651	3.32706828	0.93312569	3.56550924	0.00036315	0.00235321	Bacteria	Bacteroidetes	Cytophagia	Cytophagales	Cytophagaceae	Leadbetterella
787709	18.2634084	3.27791749	0.90047994	3.64018933	0.00027244	0.00187808	Bacteria	Actinobacteri	Actinobacteri	Actinomyceta	Actinomycetaceae	Actinomyces
1101451	20.4988778	3.27018582	0.82464154	3.96558461	7.32E-05	0.000637	Bacteria	Actinobacteri	Actinobacteri	Actinomyceta	Micrococcaceae	Micrococcus
130030	2.87666785	3.25800194	0.94659596	3.44180842	0.00057784	0.003404	Bacteria	Firmicutes	Clostridia	Clostridiales	Clostridiaceae	Caloramator
969805	10.5780611	3.1208659	0.64229129	4.85895724	1.18E-06	1.39E-05	Bacteria	Proteobacteri	Alphaproteol	Rhizobiales	Rhizobiaceae	Agrobacterium
539735	3170.82046	3.10138114	0.75727568	4.09544532	4.21E-05	0.00040153	Bacteria	Actinobacteri	Actinobacteri	Actinomyceta	Dietziaceae	Dietzia
4448102	26.3362792	3.03585315	0.56588534	5.3647849	8.10E-08	1.42E-06	Bacteria	Actinobacteri	Thermoleoph	Gaiellales		
831179	73.5094361	3.02091735	0.89440652	3.37756634	0.0007313	0.00405029	Bacteria	Proteobacteri	Betaproteoba	Burkholderia	Comamonadaceae	Rubrivivax
100640	51.1756254	2.9778441	0.72555894	4.10420704	4.06E-05	0.00039238	Bacteria	Proteobacteri	Betaproteoba	IS-44		
269031	3.58074615	2.96654982	0.91410461	3.2453067	0.00117324	0.00603382	Bacteria	Firmicutes	Clostridia	OPB54		
New.Referen	11.649776	2.96407062	0.87221077	3.39834214	0.00067796	0.00385364	Bacteria	Planctomycet	Planctomycet	Pirellulales	Pirellulaceae	Pirellula
663885	8.66740306	2.95776968	0.85143162	3.47387815	0.00051299	0.00313604	Bacteria	Bacteroidetes	Bacteroidia	Bacteroidales	Prevotellaceae	Prevotella
314567	365.566861	2.95338465	0.44363169	6.65728957	2.79E-11	8.22E-10	Bacteria	Planctomycet	Planctomycet	Gemmatales	Gemmataceae	Gemmata
1083508	1304.07826	2.9221232	0.59105128	4.94394195	7.66E-07	9.92E-06	Bacteria	Proteobacteri	Gammaprote	Xanthomona	Xanthomonadaceae	Stenotrophomonas
177302	445.247638	2.86483175	0.55216572	5.18835493	2.12E-07	3.20E-06	Bacteria	Chloroflexi	Ktedonobact	Thermogemn	Thermogemmatisporaceae	
811449	136.176384	2.7861932	0.56815633	4.90392004	9.39E-07	1.15E-05	Bacteria	Proteobacteri	Alphaproteol	Caulobacteral	Caulobacteraceae	Caulobacter
687206	175.842127	2.77254752	0.8466245	3.27482554	0.00105727	0.00557002	Bacteria	Acidobacteria	[Chloracidob	RB41	Ellin6075	
516569	1932.14342	2.75714916	0.60671617	4.54438056	5.51E-06	6.26E-05	Bacteria	Verrucomicro	Verrucomicro	Verrucomicro	Verrucomicrobiacea	Prosthecobacter
539866	56.8671782	2.72655773	0.78623152	3.46788148	0.00052458	0.00314747	Bacteria	Proteobacteri	Gammaprote	Thiotrichales	Piscirickettsiaceae	
New.Referen	78.2825459	2.72443675	0.52102684	5.22897577	1.70E-07	2.63E-06	Bacteria	Planctomycet	BD7-11			
567840	19.5787541	2.70276683	0.75015125	3.60296252	0.00031461	0.00210173	Bacteria	Proteobacteri	Alphaproteol	Rhizobiales	Bradyrhizobiaceae	Bosea
583489	196.411775	2.65151448	0.51445064	5.15406972	2.55E-07	3.67E-06	Bacteria	Proteobacteri	Betaproteobacteria			
560704	886.677697	2.62777648	0.35240733	7.45664532	8.88E-14	3.03E-12	Bacteria	Bacteroidetes	Cytophagia	Cytophagales	Cytophagaceae	
515709	525.248866	2.59736663	0.40702752	6.38130472	1.76E-10	4.44E-09	Bacteria	Proteobacteri	Alphaproteol	Rhizobiales	Hyphomicrobiaceae	Rhodoplanes
558264	90.6959621	2.56595363	0.61735193	4.15638718	3.23E-05	0.00032736	Bacteria	Proteobacteri	Betaproteoba	Burkholderia	Alcaligenaceae	Achromobacter
209782	31.1496876	2.56057364	0.72678958	3.52312927	0.00042648	0.00268312	Bacteria	Chlorobi	BSV26	A89		
1084865	87.0834721	2.55641529	0.57174376	4.47126049	7.78E-06	8.69E-05	Bacteria	Firmicutes	Bacilli	Bacillales	Staphylococcaceae	Staphylococcus

818854	125.490938	2.51085277	0.75066975	3.34481676	0.00082337	0.00452155	Bacteria	Proteobacteri	Alphaproteol	Rhodobactera	Rhodobacteraceae	Rhodobacter
579608	41.268171	2.49409391	0.61803486	4.03552306	5.45E-05	0.00049033	Bacteria	Firmicutes	Bacilli	Lactobacillale	Streptococcaceae	Streptococcus
New.Referen	12.8060345	2.43858033	0.73514442	3.31714456	0.00090943	0.0049109	Bacteria	Chloroflexi	Ktedonobact	JG30-KF-AS9		
New.Referen	7.22138205	2.40407112	0.58048984	4.14145254	3.45E-05	0.00034405	Bacteria	Chlamydiae	Chlamydiia			
238813	12.7735485	2.35529575	0.69360619	3.39572478	0.00068447	0.00385685	Bacteria	Firmicutes	Clostridia	Clostridiales	Peptococcaceae	Desulfosporosinus
1013670	1934.63414	2.3183812	0.62321583	3.72002939	0.0001992	0.00148369	Bacteria	Proteobacteri	Gammaprote	Oceanospirill	Halomonadaceae	Halomonas
984831	57.4257934	2.30351683	0.65196477	3.53319216	0.00041057	0.00260835	Bacteria	Actinobacteri	Actinobacteri	Actinomyceta	Corynebacteriaceae	Corynebacterium
New.Referen	424.220763	2.29942368	0.37135722	6.19194548	5.94E-10	1.33E-08	Bacteria	Planctomycet	Planctomycet	Gemmatales	Isosphaeraceae	
New.Referen	5.53982114	2.29148936	0.55643419	4.11816779	3.82E-05	0.00037495	Bacteria	Chlamydiae	Chlamydiia	Chlamydiales	Parachlamydiaceae	
537953	58.3079702	2.28584122	0.40332252	5.6675268	1.45E-08	2.61E-07	Bacteria	Planctomycet	Planctomycet	Pirellulales	Pirellulaceae	A17
4327003	110.46877	2.24745731	0.42441573	5.29541477	1.19E-07	1.92E-06	Bacteria	WPS-2				
41476	361.373096	2.22830914	0.69953548	3.18541262	0.00144548	0.007261	Bacteria	Proteobacteri	Gammaprote	Oceanospirill	Alcanivoracaceae	Alcanivorax
222768	316.943848	2.17991182	0.38184892	5.70883325	1.14E-08	2.11E-07	Bacteria	Proteobacteri	Alphaproteol	Ellin329		
New.Referen	302.414515	2.09118656	0.40725972	5.13477383	2.82E-07	3.98E-06	Bacteria	Planctomycet	Planctomycet	Pirellulales	Pirellulaceae	
1081222	10.1801815	2.05328817	0.56078748	3.66143724	0.0002508	0.00178595	Bacteria	Firmicutes	Bacilli	Bacillales	Paenibacillaceae	Paenibacillus
837424	132.899979	2.00809194	0.64998906	3.0894242	0.00200545	0.00962615	Bacteria	Proteobacteri	Alphaproteol	Rhodobactera	Hyphomonadaceae	
210236	29.3832654	-2.0087537	0.51592216	-3.8935209	9.88E-05	0.00080069	Bacteria	OP3	koll11			
831193	5.00661759	-2.0689506	0.58842357	-3.5160906	0.00043795	0.00270279	Bacteria	Proteobacteri	Betaproteoba	Rhodocyclale	Rhodocyclaceae	Propionivibrio
New.Referen	8.87779837	-2.150079	0.54241155	-3.9639256	7.37E-05	0.000637	Bacteria	Elusimicrobia	Endomicrobia			
New.Referen	41.110704	-2.2459453	0.44099175	-5.0929418	3.53E-07	4.76E-06	Bacteria	Proteobacteri	Alphaproteol	Rickettsiales	Rickettsiaceae	
4483076	2.64003403	-2.3263605	0.63844862	-3.6437708	0.00026867	0.00187204	Bacteria	Proteobacteria				
New.Referen	3.24759572	-2.3618112	0.65485007	-3.6066441	0.00031018	0.00209373	Bacteria	Proteobacteri	Deltaproteob	Bdellovibrior	Bdellovibrionaceae	
New.Referen	25.3052557	-2.3648238	0.60632904	-3.9002318	9.61E-05	0.00080069	Bacteria	Bacteroidetes	Cytophagia	Cytophagales	Cytophagaceae	Cytophaga
627435	31.4463387	-2.3913107	0.38781772	-6.1660686	7.00E-10	1.51E-08	Bacteria	Proteobacteri	Deltaproteob	Myxococcale	0319-6G20	
220050	6.37478775	-2.4369612	0.73990625	-3.293608	0.0009891	0.0052536	Bacteria	Elusimicrobia	Elusimicrobia	MVP-88		
163061	118.632726	-2.4833247	0.63025503	-3.9401902	8.14E-05	0.00069419	Bacteria	Proteobacteri	Gammaprote	Alteromonad	Shewanellaceae	Shewanella
719367	3580.90469	-2.5662784	0.32809607	-7.8217286	5.21E-15	1.88E-13	Bacteria	Proteobacteri	Betaproteoba	Burkholderia	Comamonadaceae	Rhodoferax
791738	82.82309	-2.7521814	0.53340284	-5.1596676	2.47E-07	3.64E-06	Bacteria	Proteobacteri	Betaproteoba	Burkholderia	Comamonadaceae	Polaromonas
590601	1166.97446	-2.9854216	0.52032004	-5.737664	9.60E-09	1.94E-07	Bacteria	Proteobacteri	Betaproteoba	Burkholderia	Oxalobacteraceae	
1143661	4.49647136	-3.3709952	0.94930273	-3.5510223	0.00038374	0.002462	Bacteria	Proteobacteri	Betaproteoba	Neisseriales	Neisseriaceae	Vogesella
225453	154.172374	-3.4206857	0.4993802	-6.8498623	7.39E-12	2.40E-10	Bacteria	Proteobacteri	Gammaprote	Alteromonad	Alteromonadaceae	Cellvibrio
274442	12.6814303	-3.6047633	1.1352013	-3.1754397	0.0014961	0.00740054	Bacteria	Tenericutes	Mollicutes	Acholeplasm	Acholeplasmataceae	Acholeplasma
944197	39.0861013	-3.609201	0.44224834	-8.1610278	3.32E-16	1.44E-14	Bacteria	Proteobacteri	Betaproteoba	Burkholderia	Oxalobacteraceae	Herminiimonas
1060517	1924.53034	-3.9686787	0.49177039	-8.0701863	7.02E-16	2.84E-14	Bacteria	Proteobacteri	Betaproteoba	Burkholderia	Oxalobacteraceae	Janthinobacterium
4369890	7.68264208	-4.2337538	1.2034692	-3.5179577	0.00043488	0.00270279	Bacteria	Proteobacteri	Epsilonprote	Campylobact	Helicobacteraceae	Sulfurimonas
335484	9.06260365	-4.2571249	0.81396501	-5.230108	1.69E-07	2.63E-06	Bacteria	Proteobacteri	Betaproteoba	Rhodocyclale	Rhodocyclaceae	Uliginosibacterium
566578	123.483142	-4.4011615	0.56049831	-7.8522297	4.09E-15	1.56E-13	Bacteria	Proteobacteri	Betaproteoba	Burkholderia	Comamonadaceae	Limnobacter
665721	6.44971458	-4.487045	1.10031853	-4.077951	4.54E-05	0.00042669	Bacteria	Proteobacteri	Gammaprote	Thiotrichales	Piscirickettsiaceae	Methylophaga
1027143	6.544507	-4.7681716	0.97063219	-4.9124392	9.00E-07	1.12E-05	Bacteria	Proteobacteri	Gammaprote	Alteromonad	Alteromonadaceae	HTCC2207
161298	30.6750244	-4.7970462	0.90436613	-5.3043188	1.13E-07	1.88E-06	Bacteria	Proteobacteri	Gammaprote	Oceanospirill	Oceanospirillaceae	
610107	72.6704771	-4.8850556	0.76920696	-6.3507688	2.14E-10	5.14E-09	Bacteria	Bacteroidetes	Cytophagia	Cytophagales	Cytophagaceae	Flectobacillus
646909	584.182097	-5.5801013	0.84501186	-6.6035775	4.01E-11	1.13E-09	Bacteria	Proteobacteri	Gammaprote	Alteromonad	Alteromonadaceae	ZD0117
830290	7086.73229	-6.2916493	0.55962819	-11.242553	2.52E-29	4.08E-27	Bacteria	Proteobacteri	Gammaprote	Vibrionales	Pseudoalteromonada	Pseudoalteromonas
789831	213.072692	-6.8469859	0.75285168	-9.0947341	9.48E-20	6.14E-18	Bacteria	Proteobacteri	Alphaproteol	Rhodobactera	Rhodobacteraceae	Phaeobacter
8882	234.988934	-6.8995809	0.82802032	-8.3326227	7.91E-17	3.66E-15	Bacteria	Proteobacteri	Gammaprote	Alteromonad	Alteromonadaceae	Alteromonas
114081	840.288171	-7.0413837	0.75062498	-9.3806946	6.55E-21	5.31E-19	Bacteria	Proteobacteri	Gammaprote	Alteromonad	Alteromonadaceae	Glaciecola
829814	2010.61429	-7.0736942	0.64865722	-10.905135	1.09E-27	1.18E-25	Bacteria	Proteobacteri	Alphaproteol	Rhodobactera	Rhodobacteraceae	Loktanella
509913	4341.2136	-7.137624	0.60468319	-11.803907	3.73E-32	8.05E-30	Bacteria	Proteobacteri	Gammaprote	Alteromonad	Alteromonadaceae	Marinobacter
1082846	99.3678055	-7.4073913	0.81741409	-9.0619814	1.28E-19	7.55E-18	Bacteria	Actinobacteri	Actinobacteri	Actinomyceta	Streptomycetaceae	Streptomyces
348517	546.247557	-8.568984	0.72390097	-11.837232	2.51E-32	8.05E-30	Bacteria	Proteobacteri	Alphaproteol	Kiloniellales	Kiloniellaceae	Thalassospira
New.CleanU	113.70164	-9.7197136	0.89472504	-10.863353	1.72E-27	1.60E-25	Bacteria	Proteobacteri	Alphaproteol	Rhodobactera	Rhodobacteraceae	Anaerospora
609349	56.993721	-9.7286019	1.07482163	-9.051364	1.41E-19	7.62E-18	Bacteria	Proteobacteri	Alphaproteol	Rhodobactera	Hyphomonadaceae	Hyphomonas
527288	182.966433	-11.668793	0.94178886	-12.390031	2.96E-35	1.92E-32	Bacteria	Bacteroidetes	Flavobacterii	Flavobacteria	Flavobacteriaceae	Muricauda

Table S7. DESeq2 Results Differentially Enriched HF- vs HF+ Day 21

OTU	baseMean	log2FoldChan	lfcSE	stat	pvalue	padj	Kingdom	Phylum	Class	Order	Family	Genus
653733	10.3773306	6.75734518	1.9247064	3.51084465	0.00044669	0.00593926	Bacteria	Proteobacteria	Gammaproteobacteria	Alteromonadales	HTCC2188	HTCC
3038080	4.27142184	5.596313413	1.43281677	3.90581233	9.39E-05	0.00153243	Bacteria	Proteobacteria	Gammaproteobacteria	Alteromonadales	211ds20	
816208	3.96707087	5.183992098	1.16487281	4.45026448	8.58E-06	0.00034211	Bacteria	Acidobacteria				
247055	4.53312463	5.173507542	1.55033671	3.33702188	0.00084681	0.00980664	Bacteria	Actinobacteria	Acidimicrobiia	Acidimicrobiales	OCS155	
New.Refe	14.2369507	4.955183633	0.92394374	5.3630794	8.18E-08	9.79E-06	Bacteria	Proteobacteria	Betaproteobacteria	SC-I-84		
810450	7.69766774	4.107815496	0.9689872	4.23928768	2.24E-05	0.00065644	Bacteria	Chloroflexi	S085			
876170	14.2371623	3.652280346	0.74646657	4.89275807	9.94E-07	7.14E-05	Bacteria	Actinobacteria	Actinobacteria	Actinomycetales	Microbacteriaceae	Salinibacterium
837424	25.0305247	3.370139822	0.88523089	3.80707438	0.00014062	0.0021949	Bacteria	Proteobacteria	Alphaproteobacteria	Rhodobacterales	Hyphomonadaceae	
854368	17.0207955	2.613306525	0.75169887	3.47653381	0.00050794	0.00651252	Bacteria	Actinobacteria	Acidimicrobiia	Acidimicrobiales	EB1017	
225453	40.8698635	-2.333044716	0.69821177	-3.3414572	0.0008334	0.00980664	Bacteria	Proteobacteria	Gammaproteobacteria	Alteromonadales	Alteromonadaceae	Cellvibrio
1106617	1060.92285	-2.651530364	0.66076475	-4.0128205	6.00E-05	0.00121311	Bacteria	Proteobacteria	Betaproteobacteria	Burkholderiales	Comamonadaceae	Limnohabitans
786420	282.674751	-2.691000694	0.68208928	-3.9452323	7.97E-05	0.00140381	Bacteria	Verrucomicrobia	[Spartobacteria]	[Chthoniobacterales]	[Chthoniobacteraceae]	Candidatus Xiphinematobacter
New.Refe	114.526916	-2.775487702	0.70477206	-3.9381353	8.21E-05	0.00140381	Bacteria	Armatimonadetes	[Fimbriimonadia]	[Fimbriimonadales]	[Fimbriimonadaceae]	Fimbriimonas
60591	90.7041581	-2.918981236	0.6906925	-4.2261661	2.38E-05	0.00065644	Bacteria	Elusimicrobia	Elusimicrobia	Elusimicrobiales		
357721	58.2087714	-3.072579087	0.56783754	-5.4110179	6.27E-08	9.79E-06	Bacteria	Elusimicrobia	Elusimicrobia	FAC88		
564411	358.879311	-3.10634178	0.69146891	-4.492381	7.04E-06	0.00031606	Bacteria	Proteobacteria	Betaproteobacteria	Burkholderiales	Oxalobacteraceae	Polynucleobacter
New.Refe	484.787933	-3.264956455	0.94932596	-3.4392365	0.00058336	0.00722156	Bacteria	OD1	ZB2			
524618	64.9400875	-3.389162437	0.92892438	-3.6484804	0.0002638	0.00378811	Bacteria	Proteobacteria	Gammaproteobacteria	Pseudomonadales	Moraxellaceae	Perlucidibaca
4300564	444.494054	-3.436304274	0.85976331	-3.9968027	6.42E-05	0.00121311	Bacteria	Proteobacteria	Alphaproteobacteria	Caulobacterales	Caulobacteraceae	Asticcacaulis
590601	582.422244	-3.487668406	0.79140872	-4.4069118	1.05E-05	0.00037643	Bacteria	Proteobacteria	Betaproteobacteria	Burkholderiales	Oxalobacteraceae	
149505	14.2843264	-3.538256172	0.87116878	-4.0615048	4.88E-05	0.00116693	Bacteria	Proteobacteria	Alphaproteobacteria	Rhodobacterales	Hyphomonadaceae	Oceanicaulis
516569	548.27801	-3.549218617	0.7046755	-5.0366709	4.74E-07	4.25E-05	Bacteria	Verrucomicrobia	Verrucomicrobiae	Verrucomicrobiales	Verrucomicrobiaceae	Prosthecobacter
New.Clea	701.245647	-3.633954686	0.79978868	-4.5436436	5.53E-06	0.00028356	Bacteria	Verrucomicrobia	Verrucomicrobiae	Verrucomicrobiales	Verrucomicrobiaceae	
177975	1971.13665	-3.647097694	0.78386172	-4.652731	3.28E-06	0.00019599	Bacteria	Proteobacteria	Alphaproteobacteria	Sphingomonadales	Sphingomonadaceae	Novosphingobium
New.Refe	131.251302	-3.976531485	0.96995	-4.0997283	4.14E-05	0.00106068	Bacteria	Verrucomicrobia	[Methylacidiphilae]	Methylacidiphilales	LD19	
587098	14.5910718	-4.033073976	1.00619194	-4.0082551	6.12E-05	0.00121311	Bacteria	Proteobacteria	Betaproteobacteria	Gallionellales	Gallionellaceae	Gallionella
1068470	6.69770633	-4.13715881	1.13943403	-3.6308893	0.00028245	0.00389993	Bacteria	Bacteroidetes	Sphingobacteriia	Sphingobacteriales	Sphingobacteriaceae	Pedobacter
New.Refe	1005.35625	-4.729620048	0.80595826	-5.8683188	4.40E-09	1.58E-06	Bacteria	Proteobacteria	Alphaproteobacteria	Caulobacterales	Caulobacteraceae	
37406	103.925911	-4.978842764	1.23418458	-4.0341152	5.48E-05	0.00121311	Bacteria	Proteobacteria	Alphaproteobacteria	Rhodospirillales	Rhodospirillaceae	Telmatospirillum
663885	6.60483075	-5.202594475	1.41008693	-3.6895558	0.00022465	0.00336033	Bacteria	Bacteroidetes	Bacteroidia	Bacteroidales	Prevotellaceae	Prevotella
267354	19.7520495	-5.415466874	1.24598206	-4.3463442	1.38E-05	0.00045177	Bacteria	Proteobacteria	Alphaproteobacteria	Rhodospirillales	Acetobacteraceae	Acidocella

Table S8. DESeq2 Results Differentially Enriched HF- vs HF+ Day 35

OTU	baseMean	log2FoldChan	lfcSE	stat	pvalue	padj	Kingdom	Phylum	Class	Order	Family	Genus
585878	4.32180138	5.679359967	1.43188436	3.9663538	7.30E-05	0.00279411	Bacteria	WS3	PRR-12	Sediment-1		
816208	4.92496442	5.006115095	1.23927495	4.0395516	5.36E-05	0.00279411	Bacteria	Acidobacteria				
1104923	6.05155375	4.653716257	1.29485246	3.59401276	0.00032562	0.00872672	Bacteria	Proteobacteria	Betaproteobacteria	A21b	EB1003	
195967	5.44203294	4.089996662	1.0923776	3.74412352	0.00018102	0.00624276	Bacteria	Proteobacteria	Deltaproteobacteria	NB1-j		
New.Refe	275.487006	3.584487526	0.8183136	4.38033478	1.18E-05	0.00090735	Bacteria	Chloroflexi	Ktedonobacteria	Thermogemmatisporales	Thermogemmatisporaceae	
876170	23.4863883	3.439020544	0.9688427	3.54961704	0.00038579	0.00984688	Bacteria	Actinobacteria	Actinobacteria	Actinomycetales	Microbacteriaceae	Salinibacterium
New.Refe	33.5916948	3.24599365	0.73647851	4.40745197	1.05E-05	0.00090735	Bacteria	Proteobacteria	Alphaproteobacteria	Rhodobacterales	Hyphomonadaceae	
1127233	17.0701878	2.871162355	0.72126905	3.98070923	6.87E-05	0.00279411	Bacteria	Gemmatimonadetes	Gemm-1			
New.Clea	1192.38626	-2.32682923	0.4755142	-4.8932908	9.92E-07	0.00013288	Bacteria	Verrucomicrobia	Verrucomicrobiae	Verrucomicrobiales	Verrucomicrobiaceae	
564411	773.499046	-2.337398508	0.58575585	-3.9903972	6.60E-05	0.00279411	Bacteria	Proteobacteria	Betaproteobacteria	Burkholderiales	Oxalobacteraceae	Polynucleobacter
786420	952.955473	-2.437519896	0.48180142	-5.0591795	4.21E-07	7.52E-05	Bacteria	Verrucomicrobia	[Spartobacteria]	[Chthoniobacterales]	[Chthoniobacteraceae]	Candidatus Xiphinematobacter
813668	694.435576	-2.68611476	0.67429737	-3.9835759	6.79E-05	0.00279411	Bacteria	Actinobacteria	Actinobacteria	Actinomycetales	ACK-M1	
1038865	386.91218	-2.705808277	0.73363116	-3.6882407	0.00022581	0.00672412	Bacteria	Bacteroidetes	Sphingobacteriia	Sphingobacteriales	Sphingobacteriaceae	
New.Refe	87.9800301	-3.278011424	0.90157176	-3.6358852	0.00027703	0.0078151	Archaea	[Parvarchaeota]	[Parvarchaea]	WCHD3-30		
590601	1162.36445	-3.313689681	0.82953406	-3.9946397	6.48E-05	0.00279411	Bacteria	Proteobacteria	Betaproteobacteria	Burkholderiales	Oxalobacteraceae	
37406	124.619605	-3.583144845	0.96867685	-3.6990095	0.00021644	0.00672412	Bacteria	Proteobacteria	Alphaproteobacteria	Rhodospirillales	Rhodospirillaceae	Telmatospirillum
2139822	1173.6296	-3.679500846	0.78023888	-4.7158645	2.41E-06	0.00025802	Bacteria	Proteobacteria	Alphaproteobacteria	Caulobacterales	Caulobacteraceae	
177975	4243.1058	-3.826327006	0.73433115	-5.2106287	1.88E-07	5.04E-05	Bacteria	Proteobacteria	Alphaproteobacteria	Sphingomonadales	Sphingomonadaceae	Novosphingobium
267354	22.1403677	-4.851706839	1.29834653	-3.7368351	0.00018635	0.00624276	Bacteria	Proteobacteria	Alphaproteobacteria	Rhodospirillales	Acetobacteraceae	Acidocella
6374	5.67714612	-5.505402643	1.55719241	-3.5354672	0.00040705	0.00991734	Bacteria	Proteobacteria	Betaproteobacteria	Neisseriales	Neisseriaceae	Chromobacterium
New.Refe	10.3365049	-6.680792291	1.54448559	-4.3255776	1.52E-05	0.00101929	Archaea	[Parvarchaeota]	[Micrarchaea]	[Micrarchaeles]		
1028297	23.6589711	-7.02994217	1.26059237	-5.5766974	2.45E-08	1.31E-05	Bacteria	Verrucomicrobia	[Spartobacteria]	[Chthoniobacterales]	[Chthoniobacteraceae]	Ellin506

Table S9. DESeq2 Results Differentially Enriched HF- vs HF+ Day 49

OTU	baseMean	log2FoldChan	lfcSE	stat	pvalue	padj	Kingdom	Phylum	Class	Order	Family	Genus
540593	8.66886466	21.8416501	3.13583722	6.96517343	3.28E-12	2.19E-10	Bacteria	Acidobacteria	Solibacteres	Solibacterales	[Bryobacteraceae]	Bryobacter
585419	22.6228846	7.962989855	1.8581945	4.2853371	1.82E-05	0.00031733	Bacteria	Firmicutes	Clostridia	Clostridiales	Veillonellaceae	Veillonella
876170	41.318641	6.42914377	1.36830554	4.69861707	2.62E-06	5.82E-05	Bacteria	Actinobacteria	Actinobacteria	Actinomycetales	Microbacteriaceae	Salinibacterium
1104923	6.98600207	6.261028541	1.53569174	4.07700867	4.56E-05	0.00067583	Bacteria	Proteobacteria	Betaproteobacteria	A21b	EB1003	
788632	211.770902	6.25384401	0.79627769	7.85384811	4.03E-15	8.07E-13	Bacteria	Proteobacteria	Betaproteobacteria	Methylophilales	Methylophilaceae	Methylotenera
New.Refe	6.81901152	6.203623997	1.78362207	3.47810453	0.00050497	0.00420811	Bacteria	Chloroflexi	Thermomicrobia	Ellin6537		
New.Refe	350.886857	5.644029288	0.74420243	7.5839974	3.35E-14	3.49E-12	Bacteria	Chloroflexi	Ktedonobacteria	Thermogemmatisporales	Thermogemmatisporaceae	
787709	30.849388	5.355585644	1.33579808	4.00927783	6.09E-05	0.00084615	Bacteria	Actinobacteria	Actinobacteria	Actinomycetales	Actinomycetaceae	Actinomyces
New.Refe	11.7487918	5.084365068	1.52990734	3.32331568	0.00088954	0.00635387	Bacteria	Chloroflexi	Ktedonobacteria	JG30-KF-AS9		
New.Refe	11.9338525	4.742402984	1.46515198	3.23679936	0.00120878	0.00804648	Bacteria	Bacteroidetes	Flavobacteriia	Flavobacteriales	Flavobacteriaceae	Zhouia
153978	102.179026	4.715614629	0.88950507	5.3013915	1.15E-07	4.18E-06	Bacteria	Cyanobacteria	Chloroplast	Streptophyta		
1013670	739.648138	4.301585545	1.0390824	4.13979252	3.48E-05	0.00057937	Bacteria	Proteobacteria	Gammaproteobacteria	Oceanospirillales	Halomonadaceae	Halomonas
562878	189.167611	3.972423903	0.96567452	4.11362607	3.89E-05	0.00061617	Bacteria	Proteobacteria	Gammaproteobacteria	Alteromonadales	Idiomarinaceae	Idiomarina
330902	9.62958386	3.907013252	1.11737873	3.49658817	0.00047125	0.00401063	Bacteria	Proteobacteria	Betaproteobacteria	Burkholderiales		
New.Refe	77.4204562	3.8004668	1.18435256	3.2088982	0.00133245	0.00845998	Bacteria	Proteobacteria	Alphaproteobacteria	Rhodobacterales	Hyphomonadaceae	
1099802	21.4171492	3.783957011	1.1568431	3.27093363	0.00107193	0.00752232	Bacteria	Actinobacteria	Actinobacteria	Actinomycetales	Propionibacteriaceae	Propionibacterium
579608	55.946845	3.649119721	1.01254386	3.60391274	0.00031346	0.00338878	Bacteria	Firmicutes	Bacilli	Lactobacillales	Streptococcaceae	Streptococcus
New.Clear	14.0740961	3.621634834	1.11800633	3.23936882	0.00119795	0.00804648	Bacteria	Armatimonadetes	Chthonomonadetes	SJA-22		
542933	50.2650199	3.613659603	1.07889075	3.34942123	0.00080981	0.00611174	Bacteria	Actinobacteria	Acidimicrobiia	Acidimicrobiales	EB1017	
558264	137.563019	3.444943918	0.85960846	4.00757331	6.13E-05	0.00084615	Bacteria	Proteobacteria	Betaproteobacteria	Burkholderiales	Alcaligenaceae	Achromobacter
539735	27.6231672	3.393073555	1.01621174	3.33894347	0.00084098	0.00622946	Bacteria	Actinobacteria	Actinobacteria	Actinomycetales	Dietziaceae	Dietzia
615907	11.1152615	3.150411474	0.98667355	3.19296232	0.00140821	0.00880133	Bacteria	Acidobacteria	Acidobacteriia	Acidobacteriales	Koribacteraceae	Candidatus Koribacter
811720	45.4679466	2.93039117	0.77190724	3.79629965	0.00014687	0.00193889	Bacteria	Acidobacteria	Acidobacteriia	Acidobacteriales	Koribacteraceae	
832166	138.998286	2.712533178	0.71558846	3.79063295	0.00015026	0.00193889	Bacteria	Proteobacteria	Betaproteobacteria	Methylophilales	Methylophilaceae	
549231	280.210645	2.700615586	0.36206658	7.4588921	8.73E-14	6.98E-12	Bacteria	Planctomycetes	Planctomycetia	Gemmatales	Gemmataceae	
1105280	102.159356	2.580290068	0.75574707	3.41422437	0.00063964	0.00500835	Bacteria	Proteobacteria	Betaproteobacteria	Burkholderiales	Burkholderiaceae	Burkholderia
811263	57.6631046	2.465292584	0.76265633	3.23250784	0.00122709	0.00804648	Bacteria	Actinobacteria	Thermoleophilia	Gaiellales	Gaiellaceae	
206278	72.6203697	2.320312568	0.72093319	3.21848486	0.0012887	0.00831418	Bacteria	Chloroflexi	Ellin6529			
385507	320.706628	2.150831182	0.43098041	4.99055439	6.02E-07	1.72E-05	Bacteria	Planctomycetes	Planctomycetia	Planctomycetales	Planctomycetaceae	Planctomyces
560704	433.690772	2.143069739	0.41099407	5.21435685	1.84E-07	6.15E-06	Bacteria	Bacteroidetes	Cytophagia	Cytophagales	Cytophagaceae	
156986	466.852939	2.070550781	0.48027379	4.31118839	1.62E-05	0.00029524	Bacteria	Proteobacteria	Gammaproteobacteria	Xanthomonadales	Sinobacteraceae	
1038865	193.072105	-2.467099439	0.51171907	-4.8211989	1.43E-06	3.81E-05	Bacteria	Bacteroidetes	Sphingobacteriia	Sphingobacteriales	Sphingobacteriaceae	
New.Refe	121.490751	-2.474035049	0.74213994	-3.3336503	0.00085714	0.00623377	Bacteria	Chlamydiae	Chlamydiia	Chlamydiales	Rhabdochlamydiaceae	Candidatus Rhabdochlamydia
590601	325.277069	-2.560726899	0.69226717	-3.6990442	0.00021641	0.00254603	Bacteria	Proteobacteria	Betaproteobacteria	Burkholderiales	Oxalobacteraceae	
564411	326.894126	-2.701838437	0.75640737	-3.5719356	0.00035435	0.00363438	Bacteria	Proteobacteria	Betaproteobacteria	Burkholderiales	Oxalobacteraceae	Polynucleobacter
177975	1595.02008	-2.703142373	0.57457594	-4.7045868	2.54E-06	5.82E-05	Bacteria	Proteobacteria	Alphaproteobacteria	Sphingomonadales	Sphingomonadaceae	Novosphingobium
516569	349.245526	-3.102407085	0.87579773	-3.5423785	0.00039654	0.0036833	Bacteria	Verrucomicrobia	Verrucomicrobiae	Verrucomicrobiales	Verrucomicrobiaceae	Prosthecobacter
248395	67.0177616	-3.203402889	0.90728487	-3.5307575	0.00041437	0.0036833	Bacteria	Proteobacteria	Alphaproteobacteria	Caulobacterales	Caulobacteraceae	Caulobacter
New.Refe	70.884826	-3.507829075	1.11250149	-3.1531006	0.00161546	0.00988397	Bacteria	Verrucomicrobia	[Methylacidiphilae]	Methylacidiphilales	LD19	
613703	30.6302701	-3.699048515	1.13548934	-3.2576691	0.00112331	0.00774699	Bacteria	OD1				
813668	242.181554	-3.742905542	1.04622233	-3.5775432	0.00034684	0.00363438	Bacteria	Actinobacteria	Actinobacteria	Actinomycetales	ACK-M1	
New.Refe	83.3383074	-3.781863031	1.0692378	-3.5369709	0.00040474	0.0036833	Archaea	[Parvarchaeota]	[Parvarchaea]	WCHD3-30		
New.Clear	431.280635	-3.810022426	0.59542067	-6.398875	1.57E-10	8.94E-09	Bacteria	Verrucomicrobia	Verrucomicrobiae	Verrucomicrobiales	Verrucomicrobiaceae	
779795	39.7704396	-3.841072972	1.08190727	-3.5502793	0.00038482	0.0036833	Bacteria	OD1	ABY1			
New.Refe	3.86594195	-3.863206934	1.10173659	-3.5064706	0.00045409	0.00394862	Bacteria	OD1	Mb-NB09			
660685	28.3563956	-3.924711287	1.03855022	-3.7790289	0.00015744	0.00196801	Bacteria	Actinobacteria	Actinobacteria	Actinomycetales		
New.Refe	404.05093	-3.980357502	0.77396528	-5.1428114	2.71E-07	8.33E-06	Bacteria	Proteobacteria	Alphaproteobacteria	Caulobacterales	Caulobacteraceae	
New.Refe	744.835628	-4.201427297	0.71080679	-5.9107867	3.40E-09	1.57E-07	Bacteria	OD1	ZB2			
786420	350.525151	-4.233976529	0.88730406	-4.7717313	1.83E-06	4.57E-05	Bacteria	Verrucomicrobia	[Spartobacteria]	[Chthoniobacterales]	[Chthoniobacteraceae]	Candidatus Xiphinematobacter
205209	162.88733	-4.257748253	0.94766481	-4.4928842	7.03E-06	0.00013384	Bacteria	OD1	SM2F11			
267354	15.1313841	-4.265237607	1.25102696	-3.409389	0.00065109	0.00500835	Bacteria	Proteobacteria	Alphaproteobacteria	Rhodospirillales	Acetobacteraceae	Acidocella
37406	59.3543623	-4.634927613	1.34671328	-3.4416588	0.00057816	0.00471967	Bacteria	Proteobacteria	Alphaproteobacteria	Rhodospirillales	Rhodospirillaceae	Telmatospirillum
593577	2.67390332	-4.956407024	1.36613807	-3.6280425	0.00028558	0.00317309	Bacteria	Cyanobacteria	Chloroplast	Chlorophyta	Chlamydomonadaceae	
228223	9.40681062	-5.105112425	1.40244524	-3.640151	0.00027248	0.00311404	Bacteria	Proteobacteria	Alphaproteobacteria	Rhodospirillales	Rhodospirillaceae	Magnetospirillum
1049387	9.05128917	-5.106685545	1.24335436	-4.1071843	4.01E-05	0.00061617	Bacteria	Proteobacteria	Gammaproteobacteria	Xanthomonadales	Xanthomonadaceae	Pseudoxanthomonas
810672	41.1814474	-5.11854791	1.10696284	-4.6239564	3.76E-06	7.93E-05	Bacteria	Actinobacteria	Actinobacteria	Actinomycetales	Microbacteriaceae	Candidatus Rhodoluna
New.Refe	2.98750408	-5.140208269	1.45426713	-3.5345695	0.00040844	0.0036833	Bacteria	Cyanobacteria	Chloroplast	Cryptophyta		
New.Refe	5.4962523	-5.184662937	1.13256715	-4.5777974	4.70E-06	9.40E-05	Bacteria	Armatimonadetes	Armatimonadia	Armatimonadales	Armatimonadaceae	
New.Refe	3.30367291	-5.278233931	1.67545367	-3.1503312	0.00163085	0.00988397	Bacteria	AC1	HDBW-WB69			
926160	2610.7854	-5.506221386	0.68007446	-8.0964979	5.66E-16	2.26E-13	Bacteria	Proteobacteria	Alphaproteobacteria	Rhizobiales	Methylobacteriaceae	Methylobacterium
1028297	8.56207945	-6.374212647	1.17843183	-5.4090635	6.34E-08	2.53E-06	Bacteria	Verrucomicrobia	[Spartobacteria]	[Chthoniobacterales]	[Chthoniobacteraceae]	Ellin506
2614	25.103749	-7.250187778	1.22784952	-5.9047853	3.53E-09	1.57E-07	Bacteria	Bacteroidetes	Cytophagia	Cytophagales	Cyclobacteriaceae	
814282	27.1844143	-7.791717065	1.02810378	-7.5787262	3.49E-14	3.49E-12	Bacteria	Proteobacteria	Alphaproteobacteria	Sphingomonadales	Erythrobacteraceae	

Table S10. DESeq2 Results Differentially Enriched HF- vs HF+ Day 56

OTU	baseMean	log2FoldChang	lfcSE	stat	pvalue	padj	Kingdom	Phylum	Class	Order	Family	Genus
816208	23.39925	6.452145182	1.0439528	6.18049511	6.39E-10	3.15E-08	Bacteria	Acidobacteria				
New.Refer	17.0969634	6.000838555	1.31382939	4.56744126	4.94E-06	6.35E-05	Bacteria	WS3	PRR-12	Sediment-1		
New.Refer	16.935475	5.984882957	1.35659479	4.41169536	1.03E-05	0.00011546	Bacteria	Planctomycetes	028H05-P-BN-P5			
New.Refer	16.2960988	5.931868011	1.4355294	4.1321815	3.59E-05	0.00032925	Bacteria	Chloroflexi	Thermomicrobia	Ellin6537		
818854	41.8950508	5.871814145	1.45690131	4.03034448	5.57E-05	0.00048764	Bacteria	Proteobacteria	Alphaproteobacteria	Rhodobacterales	Rhodobacteraceae	Rhodobacter
New.Refer	13.6710996	5.676776004	1.42337957	3.98823766	6.66E-05	0.0005464	Bacteria	Chloroflexi	TK17	mle1-48		
560122	13.3976374	5.647604054	1.6588256	3.40457975	0.00066266	0.00401674	Bacteria	Bacteroidetes	Bacteroidia	Bacteroidales	SB-1	
806393	13.1000111	5.616969124	1.1720196	4.79255562	1.65E-06	2.60E-05	Bacteria	Proteobacteria	Deltaproteobacteria	NB1-j		
876170	120.319841	5.580829069	0.78453795	7.11352343	1.13E-12	2.23E-10	Bacteria	Actinobacteria	Actinobacteria	Actinomycetales	Microbacteriaceae	Salinibacterium
325796	11.9061405	5.480115616	1.20045019	4.56505041	4.99E-06	6.35E-05	Bacteria	Chloroflexi	TK10	B07_WMSP1	FFCH4570	
538512	18.9253179	5.312456841	0.96028717	5.53215434	3.16E-08	1.04E-06	Bacteria	Proteobacteria	Betaproteobacteria	A21b	EB1003	
1107128	16.8232599	5.115848862	1.27543934	4.01104836	6.04E-05	0.00051777	Bacteria	Verrucomicrobia	[Spartobacteria]	[Chthoniobacterales]	[Chthoniobacteraceae]	DA101
New.Refer	91.7550821	4.799096995	0.80003226	5.99862931	1.99E-09	8.71E-08	Bacteria	Proteobacteria	Betaproteobacteria	SC-I-84		
New.Refer	13.488508	4.792570795	1.22951197	3.89794561	9.70E-05	0.00074947	Bacteria	Actinobacteria	Thermoleophilia	Solirubrobacterales		
558862	7.22404439	4.755433466	1.54144182	3.08505544	0.00203514	0.00977861	Bacteria	Chlorobi	SJA-28			
2595164	8.99602773	4.627511294	1.49219099	3.10115214	0.00192769	0.00937668	Bacteria	Actinobacteria	Actinobacteria	Actinomycetales	Pseudonocardiaceae	Pseudonocardia
New.Refer	108.412252	4.577742506	0.79909553	5.72865489	1.01E-08	3.63E-07	Bacteria	Proteobacteria	Alphaproteobacteria	Rhodobacterales	Hyphomonadaceae	
3038080	34.6610115	4.227835266	0.93355429	4.52875139	5.93E-06	7.08E-05	Bacteria	Proteobacteria	Gammaproteobacteria	Alteromonadales	211ds20	
New.Refer	36.7828549	4.174246971	1.26805228	3.29185716	0.00099528	0.00560201	Bacteria	Chloroflexi	Ktedonobacteria	JG30-KF-AS9		
1130903	51.1720628	4.085575416	1.08276172	3.77329134	0.00016111	0.00113351	Bacteria	Proteobacteria	Gammaproteobacteria	Oceanospirillales	Oceanospirillaceae	Marinobacterium
820978	714.194063	3.867238074	0.9414329	4.10782123	3.99E-05	0.00035765	Bacteria	Proteobacteria	Gammaproteobacteria	Vibrionales	Vibrionaceae	Vibrio
1075732	22.255623	3.648878016	1.14249534	3.19377934	0.00140423	0.00737691	Bacteria	Actinobacteria	Actinobacteria	Actinomycetales		
839306	181.909817	3.636695726	1.11465525	3.2626193	0.00110388	0.00612574	Bacteria	Proteobacteria	Alphaproteobacteria	Sphingomonadales	Sphingomonadaceae	Zymomonas
532139	74.9053369	3.514304136	0.70421961	4.99035257	6.03E-07	1.48E-05	Bacteria	Proteobacteria	Betaproteobacteria	MND1		
810450	51.1318307	3.50503995	0.73019572	4.80013764	1.59E-06	2.60E-05	Bacteria	Chloroflexi	S085			
1127233	42.8170749	3.470219106	0.86975235	3.98989332	6.61E-05	0.0005464	Bacteria	Gemmatimonadetes	Gemm-1			
330902	18.5393458	3.434041342	0.75179751	4.5677743	4.93E-06	6.35E-05	Bacteria	Proteobacteria	Betaproteobacteria	Burkholderiales		
537953	136.676617	3.347264344	0.45996609	7.27719803	3.41E-13	1.34E-10	Bacteria	Planctomycetes	Planctomycetia	Pirellulales	Pirellulaceae	A17
854368	140.691717	3.266556608	0.65832225	4.9619417	6.98E-07	1.62E-05	Bacteria	Actinobacteria	Acidimicrobiia	Acidimicrobiales	EB1017	
811263	170.864298	3.079097736	0.58475531	5.26561745	1.40E-07	3.67E-06	Bacteria	Actinobacteria	Thermoleophilia	Gaiellales	Gaiellaceae	
979107	580.771548	2.728184013	0.87110224	3.13187578	0.00173693	0.00877374	Bacteria	Proteobacteria	Gammaproteobacteria	Oceanospirillales	Halomonadaceae	Halomonas
555684	57.0510194	2.62029617	0.68190439	3.84261517	0.00012173	0.00092234	Bacteria	Acidobacteria	Acidobacteria-6	CCU21		
206278	230.911514	2.606811717	0.74866918	3.48192738	0.00049782	0.00316356	Bacteria	Chloroflexi	Ellin6529			
New.Refer	72.5219915	2.583010626	0.62189181	4.15347263	3.27E-05	0.0003072	Bacteria	TM7	SC3			
New.Refer	70.825366	2.579537859	0.79977854	3.22531516	0.00125834	0.00688592	Bacteria	Planctomycetes	vadinHA49	DH61		
549231	1141.01191	2.516157625	0.36902175	6.81845341	9.20E-12	9.06E-10	Bacteria	Planctomycetes	Planctomycetia	Gemmatales	Gemmataceae	
541741	330.682432	2.384264928	0.55397206	4.30394434	1.68E-05	0.0001695	Bacteria	Acidobacteria	Acidobacteria-6	iii1-15		
565691	231.756275	2.362939393	0.73558867	3.21231075	0.00131672	0.00710667	Bacteria	Bacteroidetes	Bacteroidia	Bacteroidales		
811720	150.474838	2.350718611	0.48150018	4.88207212	1.05E-06	2.18E-05	Bacteria	Acidobacteria	Acidobacteriia	Acidobacteriales	Koribacteraceae	
832166	344.757202	2.35048693	0.53650206	4.38113309	1.18E-05	0.00012241	Bacteria	Proteobacteria	Betaproteobacteria	Methylophilales	Methylophilaceae	
615907	31.5744446	2.296839924	0.67977079	3.37884471	0.00072791	0.00434541	Bacteria	Acidobacteria	Acidobacteriia	Acidobacteriales	Koribacteraceae	Candidatus Koribacter
4448102	28.7961612	2.168320748	0.64998111	3.33597505	0.00085001	0.00499855	Bacteria	Actinobacteria	Thermoleophilia	Gaiellales		
562311	355.798483	2.163402513	0.45606241	4.74365449	2.10E-06	3.18E-05	Bacteria	Proteobacteria	Alphaproteobacteria	Rhizobiales	Hyphomicrobiaceae	Rhodoplanes
777466	230.60241	2.12937615	0.68358279	3.11502303	0.00183931	0.00905858	Bacteria	Proteobacteria	Gammaproteobacteria	Alteromonadales	Alteromonadaceae	Marinobacter
60591	186.524847	-2.083600256	0.65630964	-3.1747214	0.0014998	0.00767432	Bacteria	Elusimicrobia	Elusimicrobia	Elusimicrobiales		
801258	65.9385411	-2.258776565	0.60877502	-3.7103634	0.00020696	0.00143058	Bacteria	Verrucomicrobia	[Methylacidiphilae]	Methylacidiphilales		
1104864	321.803108	-2.400886464	0.36634044	-6.553703	5.61E-11	3.69E-09	Bacteria	Armatimonadetes	[Fimbriimonadia]	[Fimbriimonadales]	[Fimbriimonadaceae]	Fimbriimonas
New.Refer	426.870663	-2.407842598	0.75536283	-3.1876636	0.00143427	0.00743557	Bacteria	Cyanobacteria	Chloroplast	Stramenopiles		
352419	72.5931475	-2.713181144	0.81854649	-3.3146329	0.00091764	0.00531689	Bacteria	Proteobacteria	Betaproteobacteria	SBla14		
4300564	1411.47713	-2.829866798	0.74679915	-3.7893278	0.00015106	0.00108211	Bacteria	Proteobacteria	Alphaproteobacteria	Caulobacterales	Caulobacteraceae	Asticcacaulis
357721	249.609412	-2.873014921	0.65351181	-4.3962709	1.10E-05	0.00012053	Bacteria	Elusimicrobia	Elusimicrobia	FAC88		

177975	5314.11583	-2.950079566	0.62389903	-4.7284567	2.26E-06	3.30E-05	Bacteria	Proteobacteria	Alphaproteobacteria	Sphingomonadales	Sphingomonadaceae	Novosphingobium
238813	35.8630023	-2.968710615	0.90081329	-3.2955893	0.00098215	0.00560201	Bacteria	Firmicutes	Clostridia	Clostridiales	Peptococcaceae	Desulfosporosinus
New.Refe	655.876475	-2.996496686	0.65794976	-4.5542941	5.26E-06	6.47E-05	Bacteria	Chlamydiae	Chlamydiia	Chlamydiales	Rhabdochlamydiaceae	Candidatus Rhabdochlamydia
3025055	250.592285	-3.047481804	0.80223776	-3.7987265	0.00014544	0.00106118	Bacteria	Bacteroidetes	[Saprospirae]	[Saprospirales]		
1106617	2532.58255	-3.077207682	0.44393845	-6.9316088	4.16E-12	5.46E-10	Bacteria	Proteobacteria	Betaproteobacteria	Burkholderiales	Comamonadaceae	Limnohabitans
590601	1356.67918	-3.349995918	0.7852723	-4.2660309	1.99E-05	0.000196	Bacteria	Proteobacteria	Betaproteobacteria	Burkholderiales	Oxalobacteraceae	
813668	843.304047	-3.439509055	0.89683981	-3.8351431	0.00012549	0.0009329	Bacteria	Actinobacteria	Actinobacteria	Actinomycetales	ACK-M1	
786420	1532.73758	-3.491084746	0.75897366	-4.5997443	4.23E-06	5.95E-05	Bacteria	Verrucomicrobia	[Spartobacteria]	[Chthoniobacterales]	[Chthoniobacteraceae]	Candidatus Xiphinematobacter
205209	863.928027	-3.928644375	0.89537552	-4.3877058	1.15E-05	0.00012198	Bacteria	OD1	SM2F11			
107651	43.0541809	-4.000078974	1.25172304	-3.1956582	0.00139512	0.00737691	Bacteria	Armatimonadetes	Armatimonadia	Armatimonadales	Armatimonadaceae	
564411	1261.98091	-4.021787024	0.91063662	-4.4164565	1.00E-05	0.00011546	Bacteria	Proteobacteria	Betaproteobacteria	Burkholderiales	Oxalobacteraceae	Polynucleobacter
613703	106.009043	-4.024949774	1.01674864	-3.9586478	7.54E-05	0.00060151	Bacteria	OD1				
New.Clear	1191.07567	-4.158172434	0.84417023	-4.9257511	8.40E-07	1.84E-05	Bacteria	Verrucomicrobia	Verrucomicrobiae	Verrucomicrobiales	Verrucomicrobiaceae	
New.Refe	3473.7299	-4.178339159	0.71468685	-5.8463916	5.02E-09	1.98E-07	Bacteria	OD1	ZB2			
1038865	754.315212	-4.184596961	0.76687743	-5.4566699	4.85E-08	1.47E-06	Bacteria	Bacteroidetes	Sphingobacteriia	Sphingobacteriales	Sphingobacteriaceae	
37406	305.517435	-4.227659212	0.87149846	-4.8510231	1.23E-06	2.27E-05	Bacteria	Proteobacteria	Alphaproteobacteria	Rhodospirillales	Rhodospirillaceae	Telmatospirillum
693574	18.6590276	-4.236049427	1.35653238	-3.1227043	0.00179198	0.0089372	Bacteria	SR1				
516569	900.915531	-4.437487496	0.67489431	-6.5750851	4.86E-11	3.69E-09	Bacteria	Verrucomicrobia	Verrucomicrobiae	Verrucomicrobiales	Verrucomicrobiaceae	Prosthecobacter
New.Refe	1771.86043	-4.737066913	0.72919718	-6.4962771	8.23E-11	4.63E-09	Bacteria	Proteobacteria	Alphaproteobacteria	Caulobacterales	Caulobacteraceae	
228223	29.652518	-5.442001123	1.55359795	-3.5028375	0.00046033	0.00297328	Bacteria	Proteobacteria	Alphaproteobacteria	Rhodospirillales	Rhodospirillaceae	Magnetospirillum
New.Refe	56.8208388	-5.451461451	1.53800329	-3.5445057	0.00039335	0.002583	Archaea	[Parvarchaeota]	[Micrarchaea]	[Micrarchaeles]		
267354	72.9304276	-5.521211651	1.04390389	-5.2890038	1.23E-07	3.46E-06	Bacteria	Proteobacteria	Alphaproteobacteria	Rhodospirillales	Acetobacteraceae	Acidocella
1028297	38.8940516	-7.470362701	1.54189754	-4.8449151	1.27E-06	2.27E-05	Bacteria	Verrucomicrobia	[Spartobacteria]	[Chthoniobacterales]	[Chthoniobacteraceae]	Ellin506

418

Table S11. DESeq2 Results Enrichment Day 56 no-DBNPA vs Day 0

OTU	baseMean	log2FoldCh:	lfcSE	stat	pvalue	padj	Kingdom	Phylum	Class	Order	Family	Genus
New.Referen	11295.5813	11.3951602	0.86971052	13.1022449	3.20E-39	3.64E-37	Bacteria	Bacteroidetes	Flavobacteriia	Flavobacteriales	Flavobacteriaceae	Zhouia
1501738	385.390752	7.82408856	1.21029003	6.46463938	1.02E-10	1.22E-09	Bacteria	Proteobacteria	Betaproteobacteria	Rhodocyclales	Rhodocyclaceae	
104155	1140.78554	6.10270384	0.58038494	10.5149246	7.37E-26	3.15E-24	Bacteria	Proteobacteria	Gammaproteobacteria	Xanthomonadales	Sinobacteraceae	Nevskia
New.Referen	7.50899149	5.96649629	1.53092511	3.89731429	9.73E-05	0.00040756	Bacteria	Bacteroidetes	Flavobacteriia	Flavobacteriales	Flavobacteriaceae	Gilvibacter
556648	159.08711	5.54161358	1.02406637	5.41138126	6.25E-08	4.69E-07	Bacteria	Acidobacteria	[Chloracidobacteria]	PK29		
674655	5.34211517	5.48912815	1.30654783	4.20124547	2.65E-05	0.00012418	Bacteria	Proteobacteria	Alphaproteobacteria	Sphingomonadales	Sphingomonadaceae	Sphingopyxis
4017244	70.5932311	5.09980114	1.11884855	4.55807995	5.16E-06	2.84E-05	Bacteria	Spirochaetes	[Leptospirae]	[Leptospirales]	Leptospiraceae	Turneriella
607117	3.78782845	5.09445941	0.63394394	8.03613556	9.27E-16	2.35E-14	Bacteria	Bacteroidetes	Flavobacteriia	Flavobacteriales	Flavobacteriaceae	Myroides
1049387	3.55317074	4.89382997	1.32076472	3.70530036	0.00021114	0.00081016	Bacteria	Proteobacteria	Gammaproteobacteria	Xanthomonadales	Xanthomonadaceae	Pseudoxanthomonas
665487	42.7763369	4.88247769	1.26402772	3.86263497	0.00011217	0.00046715	Bacteria	Acidobacteria	Solibacteres	Solibacterales	[Bryobacteraceae]	
New.Referen	252.530022	4.69861045	0.63496994	7.39973687	1.36E-13	2.17E-12	Bacteria	Planctomycetes	Phycisphaerae	Phycisphaerales		
New.Referen	3.07635748	4.6451774	1.3958439	3.32786309	0.00087515	0.00276725	Bacteria	Proteobacteria	Gammaproteobacteria	Xanthomonadales	Sinobacteraceae	Alkanibacter
580625	2641.93074	4.61070323	0.4170048	11.056715	2.03E-28	1.16E-26	Bacteria	Proteobacteria	Alphaproteobacteria	Rhizobiales	Bradyrhizobiaceae	Bradyrhizobium
563536	3.84377416	4.24784144	1.29963266	3.26849391	0.00108122	0.00331152	Bacteria	Proteobacteria	Gammaproteobacteria	Oceanospirillales	Halomonadaceae	Haererehalobacter
New.Referen	50.1016914	4.22169121	1.00963161	4.18141744	2.90E-05	0.0001346	Bacteria	Planctomycetes	OM190	CL500-15		
4614	37.348757	4.16130556	0.90968099	4.57446687	4.77E-06	2.69E-05	Bacteria	Proteobacteria	Alphaproteobacteria	Rhizobiales	Hyphomicrobiaceae	Pedomicrobium
308302	2228.03379	4.15712536	0.37107825	11.2028269	3.95E-29	2.45E-27	Bacteria	Planctomycetes	Planctomycetia	Planctomycetales	Planctomycetaceae	Planctomyces
New.Referen	86.9130937	4.15641167	0.77173028	5.38583466	7.21E-08	5.35E-07	Bacteria	Chloroflexi	TK17			
100640	217.006593	4.07043337	0.52573474	7.74237089	9.76E-15	1.90E-13	Bacteria	Proteobacteria	Betaproteobacteria	IS-44		
831179	181.099549	3.93416443	0.82210865	4.7854556	1.71E-06	1.05E-05	Bacteria	Proteobacteria	Betaproteobacteria	Burkholderiales	Comamonadaceae	Rubrivivax
New.Referen	2.62434901	3.90254765	1.0324478	3.77989827	0.00015689	0.00063034	Bacteria	Proteobacteria	Alphaproteobacteria	Rhizobiales	Bradyrhizobiaceae	Afipia
4310715	9.26130685	3.88429333	1.27314977	3.05093196	0.00228132	0.00641211	Bacteria	Bacteroidetes	Cytophagia	Cytophagales	Cytophagaceae	Emticicia
314567	1130.32308	3.86999587	0.27440078	14.1034433	3.62E-45	6.18E-43	Bacteria	Planctomycetes	Planctomycetia	Gemmatales	Gemmataceae	Gemmata
561804	7.55308695	3.84853026	1.23303715	3.12117948	0.00180128	0.00521303	Bacteria	Proteobacteria	Alphaproteobacteria	Rhizobiales	Hyphomicrobiaceae	Parvibaculum
814282	11.0466324	3.83451434	1.07165529	3.57812291	0.00034607	0.00121838	Bacteria	Proteobacteria	Alphaproteobacteria	Sphingomonadales	Erythrobacteraceae	
583489	838.669541	3.82669155	0.36599696	10.4555284	1.38E-25	5.55E-24	Bacteria	Proteobacteria	Betaproteobacteria			
4325509	1.63199284	3.79242635	0.63031232	6.01674156	1.78E-09	1.71E-08	Bacteria	Firmicutes	Clostridia	Clostridiales	Lachnospiraceae	Robinsoniella
552017	7.16192985	3.76788749	1.00896319	3.73441523	0.00018815	0.00073433	Bacteria	NKB19	TSBW08			
3121406	1.47704221	3.70458849	0.68944308	5.37330579	7.73E-08	5.68E-07	Bacteria	Synergistetes	Synergistia	Synergistales	Synergistaceae	Cloacibacillus
573035	9.68512849	3.67583379	1.10398791	3.32959605	0.00086972	0.00276725	Bacteria	Firmicutes	Bacilli	Bacillales	Alicyclobacillaceae	Alicyclobacillus
811449	238.152134	3.59972106	0.5173224	6.9583708	3.44E-12	4.90E-11	Bacteria	Proteobacteria	Alphaproteobacteria	Caulobacterales	Caulobacteraceae	Caulobacter
516569	6907.8575	3.52336378	0.67448424	5.22378963	1.75E-07	1.25E-06	Bacteria	Verrucomicrobia	Verrucomicrobiae	Verrucomicrobiales	Verrucomicrobiaceae	Prosthecobacter
538315	4.73985309	3.51936776	1.12987455	3.11483055	0.00184051	0.00530407	Bacteria	Actinobacteria	Actinobacteria	Actinomycetales	Pseudonocardiaceae	Pseudonocardia
113500	18.1129665	3.49584978	1.05238692	3.32182936	0.00089429	0.00281476	Bacteria	Acidobacteria	Acidobacteria-6	iii1-15	mb2424	
687206	320.853472	3.49290912	0.92281608	3.78505449	0.00015367	0.00062476	Bacteria	Acidobacteria	[Chloracidobacteria]	RB41	Ellin6075	
515709	1694.99862	3.41022854	0.19547161	17.446158	3.68E-68	1.26E-65	Bacteria	Proteobacteria	Alphaproteobacteria	Rhizobiales	Hyphomicrobiaceae	Rhodoplanes
New.Referen	33.023785	3.28454417	0.79531897	4.12984515	3.63E-05	0.00016311	Bacteria	Chloroflexi	Anaerolineae	SBR1031	A4b	
1013670	2839.74001	3.21338439	0.76165183	4.2189676	2.45E-05	0.00011641	Bacteria	Proteobacteria	Gammaproteobacteria	Oceanospirillales	Halomonadaceae	Halomonas
4327003	389.502578	3.17345883	0.62955882	5.0407662	4.64E-07	3.07E-06	Bacteria	WPS-2				
New.Referen	1359.97808	3.1729188	0.47201274	6.72210409	1.79E-11	2.31E-10	Bacteria	Planctomycetes	Planctomycetia	Gemmatales	Isosphaeraceae	
New.Referen	428.332475	3.14026732	0.67996008	4.61831127	3.87E-06	2.22E-05	Bacteria	Bacteroidetes	[Saprospirae]	[Saprospirales]	Saprospiraceae	
560704	1845.79555	3.11568966	0.36331162	8.57580511	9.84E-18	3.36E-16	Bacteria	Bacteroidetes	Cytophagia	Cytophagales	Cytophagaceae	
209782	63.1850044	3.10279337	0.77347363	4.01150507	6.03E-05	0.00026081	Bacteria	Chlorobi	BSV26	A89		
1126396	35.0237396	3.09244063	0.56704352	5.45362134	4.94E-08	3.83E-07	Bacteria	Chloroflexi	Anaerolineae	WCHB1-50		
539866	37.6462872	3.08491576	0.69086335	4.46530525	8.00E-06	4.30E-05	Bacteria	Proteobacteria	Gammaproteobacteria	Thiotrichales	Piscirickettsiaceae	
New.Referen	166.503593	3.07458167	0.58913067	5.21884503	1.80E-07	1.27E-06	Bacteria	Planctomycetes	BD7-11			
4456187	40.2758672	3.05515497	0.70921738	4.30778356	1.65E-05	8.16E-05	Bacteria	BHI80-139				
547463	0.86416692	2.91619177	0.80859475	3.60649358	0.00031036	0.00111567	Archaea	Euryarchaeota	Methanobacteria	Methanobacteriales	Methanobacteriaceae	Methanobrevibacter
New.CleanU	4.27024162	2.853148	0.9629083	2.96305267	0.00304604	0.00828864	Bacteria	Proteobacteria	Alphaproteobacteria	Rhizobiales	Methylocystaceae	Rhodoblastus
567840	35.7893788	2.83946412	0.52040748	5.45623236	4.86E-08	3.82E-07	Bacteria	Proteobacteria	Alphaproteobacteria	Rhizobiales	Bradyrhizobiaceae	Bosea
New.Referen	424.62031	2.83674946	0.46685398	6.07630992	1.23E-09	1.22E-08	Bacteria	Verrucomicrobia	[Pedosphaerae]	[Pedosphaerales]		
3900307	1667.03202	2.83330836	0.55132398	5.1390987	2.76E-07	1.92E-06	Bacteria	Armatimonadetes	[Fimbriimonadia]	[Fimbriimonadales]	[Fimbriimonadaceae]	Fimbriimonas
New.Referen	249.987374	2.81362685	0.64430954	4.36688685	1.26E-05	6.38E-05	Bacteria	Armatimonadetes	Chthonomonadetes	SJA-22		
818854	74.7794651	2.78098999	0.88809044	3.13142656	0.00173959	0.00507753	Bacteria	Proteobacteria	Alphaproteobacteria	Rhodobacterales	Rhodobacteraceae	Rhodobacter
4418260	6.77185762	2.74879531	0.83201943	3.30376334	0.00095396	0.00294822	Bacteria	Proteobacteria	Betaproteobacteria	Burkholderiales	Comamonadaceae	Aquabacterium
222768	902.044768	2.74511682	0.45008843	6.09906104	1.07E-09	1.07E-08	Bacteria	Proteobacteria	Alphaproteobacteria	Ellin329		
New.Referen	15.9370752	2.70585136	0.55599273	4.86670275	1.13E-06	7.18E-06	Bacteria	Chlamydiae	Chlamydiia			
29360	4751.18652	2.69246038	0.58928094	4.56906074	4.90E-06	2.72E-05	Bacteria	Proteobacteria	Alphaproteobacteria	Rhodospirillales	Acetobacteraceae	
812105	121.723081	2.67403524	0.38103408	7.01783744	2.25E-12	3.27E-11	Bacteria	WS3	PRR-12	Sediment-1	PRR-10	
979536	16.8256636	2.6557596	0.79569538	3.33765869	0.00084487	0.00274785	Bacteria	Acidobacteria	Solibacteres	Solibacterales	Solibacteraceae	
537953	100.026694	2.64851112	0.35763565	7.40561273	1.31E-13	2.12E-12	Bacteria	Planctomycetes	Planctomycetia	Pirellulales	Pirellulaceae	A17
837424	272.1645	2.61279964	0.62668317	4.16925134	3.06E-05	0.00013964	Bacteria	Proteobacteria	Alphaproteobacteria	Rhodobacterales	Hyphomonadaceae	
New.Referen	614.588669	2.59363075	0.30489678	8.50658615	1.79E-17	5.83E-16	Bacteria	Acidobacteria	Solibacteres	Solibacterales		
518864	26.4028794	2.58193903	0.80824644	3.19449477	0.00140076	0.00417781	Bacteria	Planctomycetes	OM190	agg27		
New.Referen	3.16654313	2.53753397	0.59629921	4.25547096	2.09E-05	0.00010105	Bacteria	Bacteroidetes	Bacteroidia	Bacteroidales	Rikenellaceae	Blvii28
4448102	29.1510782	2.50710325	0.62451222	4.01449833	5.96E-05	0.00025916	Bacteria	Actinobacteria	Thermoleophilia	Gaiellales		
New.Referen	288.600568	2.47946374	0.48387002	5.12423513	2.99E-07	2.04E-06	Bacteria	Gemmatimonadet	Gemmatimonadetes	KD8-87		
876170	75.1821169	2.47383534	0.83927757	2.94757709	0.00320275	0.00857834	Bacteria	Actinobacteria	Actinobacteria	Actinomycetales	Microbacteriaceae	Salinibacterium

419

1039699	903.062037	2.38867765	0.63346445	3.77081563	0.00016271	0.00064613	Bacteria	Armatimonadetes	Chthonomonadetes	Chthonomonadales	Chthonomonadaceae	
810167	183.305809	2.29942684	0.38436034	5.98247688	2.20E-09	2.08E-08	Bacteria	Proteobacteria	Betaproteobacteria	Burkholderiales	Comamonadaceae	Methylibium
New.Referen	839.407341	2.26419225	0.56864582	3.98172674	6.84E-05	0.00029205	Bacteria	Bacteroidetes	[Saprospirae]	[Saprospirales]		
4347035	34.8073845	2.20420658	0.69610583	3.16648199	0.00154295	0.00458189	Bacteria	Acidobacteria	EC1113			
1053775	42.5733254	2.15714663	0.58436783	3.69141923	0.00022301	0.00084385	Bacteria	Proteobacteria	Alphaproteobacteria	Rhizobiales	Hyphomicrobiaceae	Devosia
806094	313.786499	2.14611134	0.58556895	3.66500192	0.00024734	0.00091314	Bacteria	Proteobacteria	Betaproteobacteria	Ellin6067		
210532	70.8726594	2.13027035	0.48734862	4.37114271	1.24E-05	6.35E-05	Bacteria	Chloroflexi	Anaerolineae	H39		
78839	986.129126	2.08348008	0.35958323	5.79415252	6.87E-09	6.09E-08	Bacteria	Acidobacteria	DA052	Ellin6513		
549231	757.750672	2.02429065	0.27079039	7.47548921	7.69E-14	1.35E-12	Bacteria	Planctomycetes	Planctomycetia	Gemmatales	Gemmataceae	
553643	12.0270626	-2.0035887	0.5229714	-3.831163	0.00012754	0.00052161	Bacteria	Acidobacteria	Holophagae	Holophagales	Holophagaceae	Geothrix
587098	61.3005212	-2.0046667	0.59680187	-3.3590154	0.00078221	0.0025685	Bacteria	Proteobacteria	Betaproteobacteria	Gallionellales	Gallionellaceae	Gallionella
565691	54.3561356	-2.0173248	0.36372195	-5.5463377	2.92E-08	2.37E-07	Bacteria	Bacteroidetes	Bacteroidia	Bacteroidales		
909170	31.0755585	-2.022491	0.64704618	-3.1257289	0.00177365	0.0051549	Bacteria	Verrucomicrobia	Verrucomicrobiae	Verrucomicrobiales	Verrucomicrobiaceae	Luteolibacter
New.Referen	35.606425	-2.0353487	0.44532279	-4.570502	4.87E-06	2.72E-05	Archaea	[Parvarchaeota]	[Micrarchaea]	[Micrarchaeles]		
137070	16.5455828	-2.0623817	0.44987902	-4.584303	4.56E-06	2.59E-05	Bacteria	OP3	koll11	GIF10	kpj58rc	
831193	9.01563202	-2.1397061	0.62006053	-3.4508019	0.00055892	0.0018713	Bacteria	Proteobacteria	Betaproteobacteria	Rhodocyclales	Rhodocyclaceae	Propionivibrio
564411	1473.92512	-2.1642551	0.53126275	-4.0737941	4.63E-05	0.00020648	Bacteria	Proteobacteria	Betaproteobacteria	Burkholderiales	Oxalobacteraceae	Polynucleobacter
New.Referen	8.40551437	-2.1876042	0.59836088	-3.6559947	0.00025619	0.00094073	Bacteria	Proteobacteria	Deltaproteobacteria	Bdellovibrionales	Bdellovibrionaceae	
New.Referen	78.2040766	-2.2135317	0.3665005	-6.0396416	1.54E-09	1.51E-08	Archaea	[Parvarchaeota]	[Parvarchaea]	YLA114		
537033	10.1914541	-2.2281512	0.56131771	-3.9695011	7.20E-05	0.00030554	Bacteria	Cyanobacteria	4C0d-2	YS2		
352419	65.6291936	-2.2519732	0.51676472	-4.3578307	1.31E-05	6.60E-05	Bacteria	Proteobacteria	Betaproteobacteria	SBla14		
New.Referen	12.8144474	-2.2612435	0.361027	-6.2633639	3.77E-10	3.90E-09	Bacteria	Spirochaetes	Spirochaetes	Spirochaetales	Spirochaetaceae	Spirochaeta
New.Referen	35.9023536	-2.3483746	0.43290914	-5.4246362	5.81E-08	4.41E-07	Bacteria	Proteobacteria	Alphaproteobacteria	BD7-3		
New.Referen	2.50972252	-2.4051066	0.81027739	-2.9682509	0.002995	0.00819468	Bacteria	GN02	GN07			
985056	35.7301104	-2.4674998	0.42850217	-5.7584301	8.49E-09	7.34E-08	Bacteria	Proteobacteria	Betaproteobacteria	Procabacteriales	Procabacteriaceae	
210236	53.777011	-2.4756718	0.59390607	-4.1684567	3.07E-05	0.00013964	Bacteria	OP3	koll11			
330043	17.4841249	-2.4945671	0.58387711	-4.272418	1.93E-05	9.50E-05	Bacteria	Firmicutes	Clostridia	Clostridiales	Clostridiaceae	Clostridium
791738	201.271449	-2.5003792	0.66297686	-3.7714426	0.00016231	0.00064613	Bacteria	Proteobacteria	Betaproteobacteria	Burkholderiales	Comamonadaceae	Polaromonas
524618	130.78562	-2.5014446	0.56527853	-4.4251541	9.64E-06	5.14E-05	Bacteria	Proteobacteria	Gammaproteobacteria	Pseudomonadales	Moraxellaceae	Perlucidibaca
4483076	6.02918071	-2.5031564	0.59987986	-4.1727628	3.01E-05	0.00013887	Bacteria	Proteobacteria				
560122	6.11938289	-2.5303662	0.76011503	-3.3289253	0.00087182	0.00276725	Bacteria	Bacteroidetes	Bacteroidia	Bacteroidales	SB-1	
845780	443.012078	-2.5370191	0.63272053	-4.0096993	6.08E-05	0.00026116	Bacteria	Bacteroidetes	Sphingobacteriia	Sphingobacteriales	Sphingobacteriaceae	
3046945	4.26587593	-2.5640957	0.72143701	-3.5541504	0.0003792	0.0013147	Bacteria	WS5				
699470	1.99087036	-2.6050199	0.69720116	-3.7363964	0.00018668	0.00073276	Bacteria	Proteobacteria	Deltaproteobacteria	Syntrophobacterales	Syntrophaceae	
529635	3.96741136	-2.6408487	0.53928523	-4.8969424	9.73E-07	6.21E-06	Bacteria	Proteobacteria	Gammaproteobacteria	Methylococcales	Crenotrichaceae	Crenothrix
559562	5.76896335	-2.7138158	0.90626611	-2.9945021	0.00274893	0.00766335	Bacteria	Proteobacteria	Gammaproteobacteria	Oceanospirillales	Oceanospirillaceae	Spongiispira
New.Referen	10.843261	-2.7450651	0.74377655	-3.690712	0.00022363	0.00084385	Bacteria	Proteobacteria	Alphaproteobacteria	Sphingomonadales	Sphingomonadaceae	Sphingobium
New.Referen	1.97465464	-2.7487842	0.80122957	-3.4307074	0.00060201	0.00200572	Bacteria	Cyanobacteria	Chloroplast	Euglenozoa		
988314	49.5052428	-2.7559448	0.54009499	-5.102704	3.35E-07	2.26E-06	Bacteria	Proteobacteria	Gammaproteobacteria	Pseudomonadales	Moraxellaceae	Acinetobacter
693574	4.52184925	-2.7660734	0.64058537	-4.3180403	1.57E-05	7.85E-05	Bacteria	SR1				
694558	82.9343849	-2.7726919	0.47672805	-5.8160872	6.02E-09	5.41E-08	Bacteria	Firmicutes	Bacilli	Bacillales	Bacillaceae	Bacillus
37406	103.093667	-2.8095336	0.65857256	-4.2660957	1.99E-05	9.70E-05	Bacteria	Proteobacteria	Alphaproteobacteria	Rhodospirillales	Rhodospirillaceae	Telmatospirillum
239345	21.9158659	-2.8133108	0.84885923	-3.3142254	0.00091897	0.00286663	Bacteria	Proteobacteria	Gammaproteobacteria	Oceanospirillales	Oceanospirillaceae	Oleispira
758197	462.757567	-2.8453159	0.44700756	-6.3652524	1.95E-10	2.22E-09	Bacteria	Proteobacteria	Deltaproteobacteria	MIZ46		
267354	49.4019109	-2.9402593	1.00090371	-2.9376045	0.00330759	0.00882454	Bacteria	Proteobacteria	Alphaproteobacteria	Rhodospirillales	Acetobacteraceae	Acidocella
616119	13.2865613	-3.0828814	0.88327232	-3.4902955	0.00048249	0.00163949	Bacteria	Firmicutes	Clostridia	Clostridiales	[Acidaminobacteraceae]	Fusibacter
New.Referen	27.2141113	-3.0919565	0.5839737	-5.2946846	1.19E-07	8.57E-07	Bacteria	Cyanobacteria	ML635J-21			
627435	68.6098041	-3.0938708	0.43661108	-7.0861024	1.38E-12	2.09E-11	Bacteria	Proteobacteria	Deltaproteobacteria	Myxococcales	0319-6G20	
New.Referen	47.7651342	-3.098987	0.93507299	-3.3141658	0.00091917	0.00286663	Bacteria	Bacteroidetes	Cytophagia	Cytophagales	Cytophagaceae	Cytophaga
2061560	1.40208779	-3.182302	0.827531	-3.8455381	0.00012029	0.00049492	Bacteria	GN02	BD1-5			
786420	503.381851	-3.1935322	0.4740383	-6.7368654	1.62E-11	2.13E-10	Bacteria	Verrucomicrobia	[Spartobacteria]	[Chthoniobacterales]	[Chthoniobacteraceae]	Candidatus Xiphinematobacter
646549	577.353381	-3.2424375	0.59585386	-5.4416657	5.28E-08	4.05E-07	Bacteria	Proteobacteria	Gammaproteobacteria	Pseudomonadales	Pseudomonadaceae	Pseudomonas
854050	18.122979	-3.2921371	0.69849156	-4.7132096	2.44E-06	1.46E-05	Bacteria	Firmicutes	Bacilli	Bacillales	Planococcaceae	Solibacillus
351345	4.64975731	-3.3189373	0.90933245	-3.6498613	0.00026238	0.00095833	Bacteria	Proteobacteria	Gammaproteobacteria	Alteromonadales	Idiomarinaceae	Pseudidiomarina
New.Referen	18.3088818	-3.3356998	0.4372962	-7.6280101	2.38E-14	4.29E-13	Bacteria	Elusimicrobia	Endomicrobia			
235630	7.29842456	-3.3438166	0.88898742	-3.7613767	0.00016898	0.00066713	Bacteria	Proteobacteria	Gammaproteobacteria	Methylococcales	Methylococcaceae	Methylomicrobium
1028297	54.8176708	-3.3472548	0.90379758	-3.7035448	0.00021261	0.00081123	Bacteria	Verrucomicrobia	[Spartobacteria]	[Chthoniobacterales]	[Chthoniobacteraceae]	Ellin506
New.Referen	19.8417707	-3.4400583	0.67058473	-5.1299384	2.90E-07	2.00E-06	Bacteria	AC1	HDBW-WB69			
612227	2.38382006	-3.4594952	0.95323662	-3.6292094	0.00028429	0.00103282	Bacteria	Proteobacteria	Deltaproteobacteria	Syntrophobacterales	Syntrophaceae	Syntrophus
791566	1.16780586	-3.4795485	0.79157478	-4.3957294	1.10E-05	5.80E-05	Bacteria	Proteobacteria	Epsilonproteobacteria	Campylobacterales	Helicobacteraceae	Sulfuricurvum
141636	35.4768295	-3.490608	0.63738482	-5.476453	4.34E-08	3.49E-07	Bacteria	Proteobacteria	Deltaproteobacteria	Bdellovibrionales	Bacteriovoracaceae	
228223	12.8144497	-3.5065234	0.73357293	-4.7800611	1.75E-06	1.07E-05	Bacteria	Proteobacteria	Alphaproteobacteria	Rhodospirillales	Rhodospirillaceae	Magnetospirillum
New.Referen	2.05838679	-3.5422686	0.9507134	-3.7259058	0.00019462	0.00075097	Bacteria	Proteobacteria	Alphaproteobacteria	Rhizobiales	Rhizobiaceae	Rhizobium
4476153	3.85144391	-3.5675003	0.96916634	-3.6809989	0.00023232	0.00087185	Bacteria	Proteobacteria	Gammaproteobacteria	Alteromonadales	Alteromonadaceae	HB2-32-21
279220	3.3870043	-3.5676195	1.05135573	-3.3933515	0.00069043	0.00228914	Bacteria	Proteobacteria	Gammaproteobacteria	Oceanospirillales	Oceanospirillaceae	Amphritea
4300564	902.954165	-3.5764304	0.56989314	-6.2756158	3.48E-10	3.67E-09	Bacteria	Proteobacteria	Alphaproteobacteria	Caulobacterales	Caulobacteraceae	Asticcacaulis
177975	2296.99092	-3.5772304	0.55793922	-6.4115055	1.44E-10	1.67E-09	Bacteria	Proteobacteria	Alphaproteobacteria	Sphingomonadales	Sphingomonadaceae	Novosphingobium
842848	45.5970111	-3.5945977	0.51178623	-7.0236311	2.16E-12	3.21E-11	Bacteria	Armatimonadetes	Armatimonadia	FW68		
578551	7.35786462	-3.6257994	0.77681788	-4.6675025	3.05E-06	1.76E-05	Bacteria	Proteobacteria	Alphaproteobacteria	Sphingomonadales		
New.Referen	5.07431293	-3.6258739	1.12366989	-3.2268141	0.00125177	0.003783	Bacteria	ZB3	BS119			
149505	19.6863785	-3.6267619	0.79863457	-4.5412032	5.59E-06	3.06E-05	Bacteria	Proteobacteria	Alphaproteobacteria	Rhodobacterales	Hyphomonadaceae	Oceanicaulis

87245	32.6395101	-3.6316415	0.5254251	-6.9118157	4.78E-12	6.67E-11	Bacteria	Proteobacteria	Deltaproteobacteria	Desulfovibrionales	Desulfovibrionaceae	Desulfovibrio
851442	6.48341434	-3.6455314	0.87572765	-4.1628598	3.14E-05	0.00014216	Bacteria	Firmicutes	Clostridia	Halanaerobiales	Halanaerobiaceae	Halanaerobium
719367	7871.75641	-3.6821119	0.49410023	-7.4521559	9.18E-14	1.57E-12	Bacteria	Proteobacteria	Betaproteobacteria	Burkholderiales	Comamonadaceae	Rhodoferax
New.Referen	2.05590298	-3.7000316	0.7913836	-4.6753959	2.93E-06	1.73E-05	Bacteria	Firmicutes	Clostridia	Clostridiales	Lachnospiraceae	Clostridium
820978	254.859066	-3.748411	0.5727143	-6.5449928	5.95E-11	7.26E-10	Bacteria	Proteobacteria	Gammaproteobacteria	Vibrionales	Vibrionaceae	Vibrio
New.Referen	6.8312425	-3.7658675	0.71120906	-5.2950219	1.19E-07	8.57E-07	Bacteria	Bacteroidetes	Bacteroidia	Bacteroidales	Bacteroidaceae	Bacteroides
2058521	1.21051187	-3.7691848	1.20226224	-3.1350771	0.00171809	0.00503629	Bacteria	Bacteroidetes	Bacteroidia	Bacteroidales	Porphyromonadaceae	Parabacteroides
248432	6.3404665	-3.7920127	1.0159929	-3.7323221	0.00018972	0.00073625	Bacteria	Proteobacteria	Gammaproteobacteria	Oceanospirillales	Oceanospirillaceae	Oleibacter
4428396	2.00528047	-3.8432216	1.07518597	-3.5744715	0.00035094	0.00122918	Bacteria	Proteobacteria	Gammaproteobacteria	Oceanospirillales	Halomonadaceae	
614357	131.746568	-3.8435635	0.57825792	-6.6467979	3.00E-11	3.72E-10	Bacteria	Proteobacteria	Alphaproteobacteria	Rhizobiales	Methylocystaceae	Methylopila
New.CleanU	0.9016668	-3.8475095	1.14696761	-3.3545058	0.00079507	0.00259824	Bacteria	Proteobacteria	Betaproteobacteria	Rhodocyclales	Rhodocyclaceae	C39
New.Referen	1.61303351	-3.9772254	0.90032346	-4.4175516	9.98E-06	5.29E-05	Bacteria	Proteobacteria	Betaproteobacteria	Neisseriales	Neisseriaceae	Chitinibacter
New.Referen	76.4810341	-3.9815097	0.37366251	-10.655363	1.65E-26	7.49E-25	Bacteria	Proteobacteria	Alphaproteobacteria	Rickettsiales	Rickettsiaceae	
137097	1.87574536	-3.9950628	0.85486599	-4.6733205	2.96E-06	1.73E-05	Bacteria	Proteobacteria	Betaproteobacteria	Neisseriales	Neisseriaceae	Deefgea
4369890	3.40549218	-4.1601956	1.29684149	-3.2079446	0.00133687	0.0040224	Bacteria	Proteobacteria	Epsilonproteobacteria	Campylobacterales	Helicobacteraceae	Sulfurimonas
220050	12.5248204	-4.1861845	0.60670042	-6.8999202	5.20E-12	7.11E-11	Bacteria	Elusimicrobia	Elusimicrobia	MVP-88		
103229	2.96353196	-4.2371805	0.8338601	-5.0814045	3.75E-07	2.51E-06	Bacteria	Bacteroidetes	Flavobacteriia	Flavobacteriales	Cryomorphaceae	Fluviicola
562701	2.93879321	-4.3327788	0.76006474	-5.7005391	1.19E-08	1.02E-07	Bacteria	Tenericutes	Mollicutes			
554951	2.38942994	-4.3344125	1.19497605	-3.6271962	0.00028652	0.0010354	Bacteria	Chloroflexi	SAR202			
277566	18.1711945	-4.3346392	0.64552943	-6.7148593	1.88E-11	2.38E-10	Bacteria	Proteobacteria	Epsilonproteobacteria	Campylobacterales	Campylobacteraceae	Arcobacter
2398623	4.09194281	-4.3483977	1.33319091	-3.2616467	0.00110767	0.00337741	Bacteria	Firmicutes	Clostridia	Clostridiales	Peptostreptococcaceae	Clostridium
New.Referen	6.89016982	-4.362421	0.90693624	-4.8100636	1.51E-06	9.45E-06	Bacteria	Cyanobacteria	4C0d-2	SM1D11		
1852120	2.83045184	-4.4015092	1.33774087	-3.2902555	0.00100096	0.00307954	Bacteria	Proteobacteria	Gammaproteobacteria	Pseudomonadales	Moraxellaceae	Alkanindiges
1143661	10.5841658	-4.4166452	0.88430462	-4.9944839	5.90E-07	3.87E-06	Bacteria	Proteobacteria	Betaproteobacteria	Neisseriales	Neisseriaceae	Vogesella
81639	3.86520877	-4.4839185	1.05814866	-4.2375128	2.26E-05	0.00010871	Bacteria	Proteobacteria	Gammaproteobacteria	Methylococcales		
212239	1.64798298	-4.5341476	1.1770327	-3.8521849	0.00011707	0.00048459	Bacteria	Acidobacteria	Acidobacteriia	Acidobacteriales	Acidobacteriaceae	Terriglobus
624158	5.09695385	-4.5431068	1.03784992	-4.3774217	1.20E-05	6.21E-05	Bacteria	Firmicutes	Bacilli	Bacillales	Bacillaceae	Natronobacillus
335484	26.6992461	-4.5897191	0.82088821	-5.5911621	2.26E-08	1.86E-07	Bacteria	Proteobacteria	Betaproteobacteria	Rhodocyclales	Rhodocyclaceae	Uliginosibacterium
225453	437.610242	-4.6857142	0.67990098	-6.8917597	5.51E-12	7.38E-11	Bacteria	Proteobacteria	Gammaproteobacteria	Alteromonadales	Alteromonadaceae	Cellvibrio
1027143	1.86086644	-4.6932737	1.15940064	-4.0480172	5.17E-05	0.00022761	Bacteria	Proteobacteria	Gammaproteobacteria	Alteromonadales	Alteromonadaceae	HTCC2207
809486	16.4517868	-4.7636598	0.75157499	-6.3382362	2.32E-10	2.56E-09	Bacteria	Bacteroidetes	Flavobacteriia	Flavobacteriales	Flavobacteriaceae	Polaribacter
1065817	102.765822	-4.8004471	0.54031135	-8.8845941	6.42E-19	2.31E-17	Bacteria	Proteobacteria	Gammaproteobacteria	Alteromonadales	Colwelliaceae	Colwellia
310419	35.8962125	-4.8081892	0.61759483	-7.7853455	6.95E-15	1.40E-13	Bacteria	Bacteroidetes	Bacteroidia	Bacteroidales	Marinilabiaceae	
191237	7.39147351	-4.8376037	1.01498616	-4.7661771	1.88E-06	1.13E-05	Bacteria	Bacteroidetes	Flavobacteriia	Flavobacteriales	[Weeksellaceae]	Chryseobacterium
768553	36.4211677	-4.8521214	0.75620073	-6.4164464	1.39E-10	1.64E-09	Bacteria	Proteobacteria	Gammaproteobacteria	Enterobacteriales	Enterobacteriaceae	Proteus
590601	3178.13743	-4.8719355	0.61534042	-7.9174639	2.42E-15	5.71E-14	Bacteria	Proteobacteria	Betaproteobacteria	Burkholderiales	Oxalobacteraceae	
535928	8.25715052	-4.904929	1.02393656	-4.7902665	1.67E-06	1.03E-05	Bacteria	Bacteroidetes	Flavobacteriia	Flavobacteriales	Flavobacteriaceae	
575486	8.42858273	-5.0337226	0.85752631	-5.8700503	4.36E-09	3.97E-08	Bacteria	Bacteroidetes	Bacteroidia	Bacteroidales	Porphyromonadaceae	Paludibacter
347439	32.7380881	-5.0849134	0.68640721	-7.4080128	1.28E-13	2.12E-12	Bacteria	Proteobacteria	Gammaproteobacteria	Alteromonadales	Psychromonadaceae	Psychromonas
309065	3.66251752	-5.098343	1.16273641	-4.3847797	1.16E-05	6.05E-05	Bacteria	Proteobacteria	Gammaproteobacteria	Xanthomonadales	Xanthomonadaceae	Rhodanobacter
562878	132.63054	-5.0992061	0.61564801	-8.2826648	1.20E-16	3.74E-15	Bacteria	Proteobacteria	Gammaproteobacteria	Alteromonadales	Idiomarinaceae	Idiomarina
1068470	11.7788216	-5.1034082	0.81326191	-6.2752333	3.49E-10	3.67E-09	Bacteria	Bacteroidetes	Sphingobacteriia	Sphingobacteriales	Sphingobacteriaceae	Pedobacter
1130903	70.4201271	-5.1719404	0.62689295	-8.2501174	1.58E-16	4.70E-15	Bacteria	Proteobacteria	Gammaproteobacteria	Oceanospirillales	Oceanospirillaceae	Marinobacterium
163061	46.0193336	-5.2829392	0.67193202	-7.862312	3.77E-15	8.05E-14	Bacteria	Proteobacteria	Gammaproteobacteria	Alteromonadales	Shewanellaceae	Shewanella
944197	130.418538	-5.3607748	0.4661125	-11.501032	1.30E-30	8.90E-29	Bacteria	Proteobacteria	Betaproteobacteria	Burkholderiales	Oxalobacteraceae	Herminiimonas
806640	16.20453	-5.4896281	0.75991843	-7.2239702	5.05E-13	7.84E-12	Bacteria	Proteobacteria	Gammaproteobacteria	Alteromonadales	Colwelliaceae	
937848	29.2938205	-5.5068075	0.87239109	-6.3123151	2.75E-10	2.98E-09	Bacteria	Verrucomicrobia	[Spartobacteria]	[Chthoniobacterales]	[Chthoniobacteraceae]	heteroC45_4W
528421	15.4122816	-5.510784	0.86913585	-6.3405323	2.29E-10	2.56E-09	Bacteria	Proteobacteria	Gammaproteobacteria	Enterobacteriales	Enterobacteriaceae	Citrobacter
665721	5.66951997	-5.7247978	0.92854512	-6.1653415	7.03E-10	7.17E-09	Bacteria	Proteobacteria	Gammaproteobacteria	Thiotrichales	Piscirickettsiaceae	Methylophaga
610107	257.506514	-5.8476087	1.010113	-5.7890639	7.08E-09	6.20E-08	Bacteria	Bacteroidetes	Cytophagia	Cytophagales	Cytophagaceae	Flectobacillus
839235	16.7392575	-5.8723884	0.76632656	-7.6630365	1.82E-14	3.35E-13	Bacteria	Proteobacteria	Gammaproteobacteria	Aeromonadales	Aeromonadaceae	
New.Referen	4.94522045	-6.042554	1.49094855	-4.0528253	5.06E-05	0.00022443	Bacteria	Cyanobacteria	Chloroplast	Cryptophyta		
6374	6.79510448	-6.1822487	0.76500246	-8.0813449	6.41E-16	1.68E-14	Bacteria	Proteobacteria	Betaproteobacteria	Neisseriales	Neisseriaceae	Chromobacterium
New.Referen	217.087757	-6.1996271	0.76202961	-8.1356774	4.10E-16	1.12E-14	Bacteria	Verrucomicrobia	[Methylacidiphilae]	Methylacidiphilales	LD19	
112983	6.24271909	-6.2589494	1.11246716	-5.626188	1.84E-08	1.53E-07	Bacteria	Proteobacteria	Gammaproteobacteria	Alteromonadales	Moritellaceae	Moritella
794061	13.3693631	-6.9514028	1.48194985	-4.6907139	2.72E-06	1.62E-05	Bacteria	Proteobacteria	Alphaproteobacteria	Rhodobacterales	Rhodobacteraceae	Octadecabacter
1082846	27.1164977	-7.2043687	0.80715062	-8.9256807	4.43E-19	1.68E-17	Bacteria	Actinobacteria	Actinobacteria	Actinomycetales	Streptomycetaceae	Streptomyces
646909	81.7568897	-7.4475962	0.94598786	-7.8728242	3.47E-15	7.64E-14	Bacteria	Proteobacteria	Gammaproteobacteria	Alteromonadales	Alteromonadaceae	ZD0117
1845162	19.171892	-7.9258353	1.01725433	-7.7914	6.63E-15	1.37E-13	Bacteria	Proteobacteria	Alphaproteobacteria	Rhodospirillales	Rhodospirillaceae	Novispirillum
161298	35.9118014	-8.099803	0.74881538	-10.816822	2.87E-27	1.51E-25	Bacteria	Proteobacteria	Gammaproteobacteria	Oceanospirillales	Oceanospirillaceae	
8882	571.930196	-8.7731543	1.0708598	-8.1926264	2.56E-16	7.27E-15	Bacteria	Proteobacteria	Gammaproteobacteria	Alteromonadales	Alteromonadaceae	Alteromonas
New.CleanU	426.204287	-9.1481918	1.19247059	-7.6716289	1.70E-14	3.22E-13	Bacteria	Proteobacteria	Alphaproteobacteria	Rhodobacterales	Rhodobacteraceae	Anaerospora
830290	15068.3551	-9.6696124	0.64339477	-15.02905	4.74E-51	1.08E-48	Bacteria	Proteobacteria	Gammaproteobacteria	Vibrionales	Pseudoalteromonadaceae	Pseudoalteromonas
509913	13863.4859	-10.264436	0.79428498	-12.922863	3.34E-38	3.26E-36	Bacteria	Proteobacteria	Gammaproteobacteria	Alteromonadales	Alteromonadaceae	Marinobacter
348517	906.543918	-10.513264	0.97739764	-10.756384	5.53E-27	2.70E-25	Bacteria	Proteobacteria	Alphaproteobacteria	Kiloniellales	Kiloniellaceae	Thalassospira
789831	736.037071	-10.794776	0.92200104	-11.707986	1.16E-31	8.80E-30	Bacteria	Proteobacteria	Alphaproteobacteria	Rhodobacterales	Rhodobacteraceae	Phaeobacter
829814	6341.15026	-10.838813	0.82004309	-13.21737	6.97E-40	9.52E-38	Bacteria	Proteobacteria	Alphaproteobacteria	Rhodobacterales	Rhodobacteraceae	Loktanella
609349	92.1397926	-10.84912	1.35994306	-7.9776281	1.49E-15	3.64E-14	Bacteria	Proteobacteria	Alphaproteobacteria	Rhodobacterales	Hyphomonadaceae	Hyphomonas
114081	2827.43201	-11.273938	0.95174235	-11.845578	2.27E-32	1.94E-30	Bacteria	Proteobacteria	Gammaproteobacteria	Alteromonadales	Alteromonadaceae	Glaciecola
527288	842.039082	-29.023881	1.21960172	-23.797836	3.52E-125	2.40E-122	Bacteria	Bacteroidetes	Flavobacteriia	Flavobacteriales	Flavobacteriaceae	Muricauda

Table S12. DESeq2 Results Enrichment Day 56 vs Day 56 no-DBNPA

OTU	baseMean	log2FoldCh	lfcSE	stat	pvalue	padj	Kingdom	Phylum	Class	Order	Family	Genus
191237	46.5912313	7.46889834	0.98181165	7.60726185	2.80E-14	9.32E-13	Bacteria	Bacteroidetes	Flavobacteriia	Flavobacteriales	[Weeksellaceae]	Chryseobacterium
833317	24.7821743	6.80066939	1.13374118	5.99843201	1.99E-09	2.58E-08	Bacteria	Firmicutes	Bacilli	Bacillales	Planococcaceae	Sporosarcina
81639	11.1578854	6.20832993	1.15637441	5.36878874	7.93E-08	6.90E-07	Bacteria	Proteobacteri	Gammaproteobacteria	Methylococcales		
575486	18.9780906	6.1874785	0.96427099	6.41674234	1.39E-10	2.15E-09	Bacteria	Bacteroidetes	Bacteroidia	Bacteroidales	Porphyromonadaceae	Paludibacter
824606	7.24030128	6.14245821	1.14854675	5.3480263	8.89E-08	7.62E-07	Bacteria	Bacteroidetes	Sphingobacteriia	Sphingobacteriales	Sphingobacteriaceae	Sphingobacterium
937848	42.2324352	5.96799091	1.06566692	5.60024039	2.14E-08	2.25E-07	Bacteria	Verrucomicr	[Spartobacteria]	[Chthoniobacterales]	[Chthoniobacteraceae]	heteroC45_4W
279204	9.38176588	5.9368998	1.30511042	4.54896361	5.39E-06	3.10E-05	Bacteria	Bacteroidetes	Flavobacteriia	Flavobacteriales	Flavobacteriaceae	Salegentibacter
624158	11.285236	5.78630375	1.03978137	5.56492349	2.62E-08	2.56E-07	Bacteria	Firmicutes	Bacilli	Bacillales	Bacillaceae	Natronobacillus
839235	15.7545826	5.76084685	0.80762451	7.13307581	9.82E-13	2.19E-11	Bacteria	Proteobacteri	Gammaproteobacteria	Aeromonadales	Aeromonadaceae	
6374	5.47551486	5.72808176	1.55228628	3.69009367	0.00022417	0.0008983	Bacteria	Proteobacteri	Betaproteobacteria	Neisseriales	Neisseriaceae	Chromobacterium
562701	8.58487473	5.72630321	0.99903791	5.73181775	9.94E-09	1.15E-07	Bacteria	Tenericutes	Mollicutes			
806640	20.2822388	5.70858075	0.83712463	6.81927222	9.15E-12	1.76E-10	Bacteria	Proteobacteri	Gammaproteobacteria	Alteromonadales	Colwelliaceae	
1065817	207.923717	5.69437621	0.64858477	8.77969457	1.64E-18	7.61E-17	Bacteria	Proteobacteri	Gammaproteobacteria	Alteromonadales	Colwelliaceae	Colwellia
New.Referen	148.740467	5.65192003	0.8623096	6.55439766	5.59E-11	9.43E-10	Bacteria	Verrucomicr	[Methylacidiphilae]	Methylacidiphilales	LD19	
310419	66.5066704	5.55904485	0.84421294	6.58488469	4.55E-11	7.92E-10	Bacteria	Bacteroidetes	Bacteroidia	Bacteroidales	Marinilabiaceae	
New.Referen	8.10135532	5.55072357	1.10590794	5.0191552	5.19E-07	3.66E-06	Bacteria	Firmicutes	Clostridia	Clostridiales	Lachnospiraceae	Clostridium
248432	19.6355966	5.46740238	1.55682591	3.51189067	0.00044493	0.0016745	Bacteria	Proteobacteri	Gammaproteobacteria	Oceanospirillales	Oceanospirillaceae	Oleibacter
694558	443.070691	5.35909833	0.56832325	9.42966576	4.11E-21	4.58E-19	Bacteria	Firmicutes	Bacilli	Bacillales	Bacillaceae	Bacillus
562878	173.652187	5.3495929	0.63822923	8.38193027	5.21E-17	2.23E-15	Bacteria	Proteobacteri	Gammaproteobacteria	Alteromonadales	Idiomarinaceae	Idiomarina
114081	50.2183704	5.33314804	0.9372906	5.68996219	1.27E-08	1.41E-07	Bacteria	Proteobacteri	Gammaproteobacteria	Alteromonadales	Alteromonadaceae	Glaciecola
41476	729.48656	5.21879365	0.93362033	5.58984575	2.27E-08	2.34E-07	Bacteria	Proteobacteri	Gammaproteobacteria	Oceanospirillales	Alcanivoracaceae	Alcanivorax
163061	47.1449414	5.21587807	0.88854408	5.87013991	4.35E-09	5.27E-08	Bacteria	Proteobacteri	Gammaproteobacteria	Alteromonadales	Shewanellaceae	Shewanella
New.Referen	16.7060202	5.07734678	0.85521356	5.93693435	2.90E-09	3.68E-08	Bacteria	Bacteroidetes	Bacteroidia	Bacteroidales	Bacteroidaceae	Bacteroides
347439	30.8053581	5.05195876	0.98906305	5.10782278	3.26E-07	2.45E-06	Bacteria	Proteobacteri	Gammaproteobacteria	Alteromonadales	Psychromonadaceae	Psychromonas
988314	227.851014	5.03219884	0.49636621	10.1380769	3.74E-24	6.95E-22	Bacteria	Proteobacteri	Gammaproteobacteria	Pseudomonadales	Moraxellaceae	Acinetobacter
New.Referen	12.7490348	5.02506935	1.15361305	4.35594011	1.32E-05	7.31E-05	Bacteria	Cyanobacteri	4C0d-2	SM1D11		
1111294	15.337319	5.01419438	0.88159374	5.68764742	1.29E-08	1.41E-07	Bacteria	Proteobacteri	Gammaproteobacteria	Enterobacteriales	Enterobacteriaceae	Escherichia
1099802	3.03175471	5.00696165	1.54908655	3.23220266	0.0012284	0.00402481	Bacteria	Actinobacteri	Actinobacteria	Actinomycetales	Propionibacteriaceae	Propionibacterium
768553	43.193723	4.98540222	1.09867647	4.53764357	5.69E-06	3.23E-05	Bacteria	Proteobacteri	Gammaproteobacteria	Enterobacteriales	Enterobacteriaceae	Proteus
780303	3.04836509	4.90599491	1.29657836	3.78380131	0.00015445	0.0006774	Bacteria	Firmicutes	Clostridia	Clostridiales	[Tissierellaceae]	Tissierella_Soehngenia
277566	28.9644597	4.86019492	0.986385	4.92727986	8.34E-07	5.46E-06	Bacteria	Proteobacteri	Epsilonproteobacteria	Campylobacterales	Campylobacteraceae	Arcobacter
575028	9.73150512	4.86015166	1.21039093	4.01535696	5.94E-05	0.00028018	Bacteria	Proteobacteri	Betaproteobacteria	Burkholderiales	Alcaligenaceae	Alcaligenes
789831	13.9727851	4.80015078	1.2727248	3.77155436	0.00016223	0.00070597	Bacteria	Proteobacteri	Alphaproteobacteria	Rhodobacterales	Rhodobacteraceae	Phaeobacter
820978	559.714054	4.78459802	0.75191122	6.36324856	1.98E-10	2.84E-09	Bacteria	Proteobacteri	Gammaproteobacteria	Vibrionales	Vibrionaceae	Vibrio
130030	8.48979201	4.77817759	0.85781033	5.57020292	2.54E-08	2.53E-07	Bacteria	Firmicutes	Clostridia	Clostridiales	Clostridiaceae	Caloramator
539735	44.8369718	4.77453231	0.74702695	6.39137894	1.64E-10	2.47E-09	Bacteria	Actinobacteri	Actinobacteria	Actinomycetales	Dietziaceae	Dietzia
1101451	28.5287669	4.74544962	0.73763404	6.43333869	1.25E-10	1.99E-09	Bacteria	Actinobacteri	Actinobacteria	Actinomycetales	Micrococcaceae	Micrococcus
926160	4703.90517	4.61070356	0.80354833	5.73792939	9.58E-09	1.14E-07	Bacteria	Proteobacteri	Alphaproteobacteria	Rhizobiales	Methylobacteriaceae	Methylobacterium
309065	2.82145714	4.57617489	1.49924096	3.05232783	0.00227074	0.00683677	Bacteria	Proteobacteri	Gammaproteobacteria	Xanthomonadales	Xanthomonadaceae	Rhodanobacter
1068470	8.6712228	4.54683341	1.11475156	4.0787863	4.53E-05	0.00022515	Bacteria	Bacteroidetes	Sphingobacteriia	Sphingobacteriales	Sphingobacteriaceae	Pedobacter
537033	46.1880032	4.52139271	0.57748527	7.8294511	4.90E-15	1.82E-13	Bacteria	Cyanobacteri	4C0d-2	YS2		
1130903	50.5106491	4.51370706	0.93234549	4.84123868	1.29E-06	8.26E-06	Bacteria	Proteobacteri	Gammaproteobacteria	Oceanospirillales	Oceanospirillaceae	Marinobacterium
235630	30.834071	4.50900605	1.47563523	3.0556373	0.00224583	0.00679851	Bacteria	Proteobacteri	Gammaproteobacteria	Methylococcales	Methylococcaceae	Methylomicrobium
1081222	23.8358678	4.46821101	0.48629671	9.18824031	3.99E-20	2.47E-18	Bacteria	Firmicutes	Bacilli	Bacillales	Paenibacillaceae	Paenibacillus
238813	21.8291225	4.43924006	0.73151321	6.06857127	1.29E-09	1.71E-08	Bacteria	Firmicutes	Clostridia	Clostridiales	Peptococcaceae	Desulfosporosinus
330043	60.0139222	4.37484476	0.45806214	9.55076702	1.29E-21	1.79E-19	Bacteria	Firmicutes	Clostridia	Clostridiales	Clostridiaceae	Clostridium
531300	32.1226758	4.35429513	0.84845509	5.1320278	2.87E-07	2.22E-06	Bacteria	Actinobacteri	Actinobacteria	Actinomycetales	Brevibacteriaceae	Brevibacterium
528421	6.88121395	4.35032875	1.16703626	3.7276723	0.00019326	0.00079736	Bacteria	Proteobacteri	Gammaproteobacteria	Enterobacteriales	Enterobacteriaceae	Citrobacter
278860	7.50465687	4.28933244	0.82557128	5.1955931	2.04E-07	1.65E-06	Bacteria	Bacteroidetes	Bacteroidia	Bacteroidales	S24-7	
830290	412.998165	4.22782244	0.57883033	7.30407894	2.79E-13	7.07E-12	Bacteria	Proteobacteri	Gammaproteobacteria	Vibrionales	Pseudoalteromonadaceae	Pseudoalteromonas
351345	9.46851699	4.18406929	1.24432389	3.36252429	0.00077233	0.0027056	Bacteria	Proteobacteri	Gammaproteobacteria	Alteromonadales	Idiomarinaceae	Pseudidiomarina
786420	960.882881	4.13987348	0.56528923	7.32346071	2.42E-13	6.41E-12	Bacteria	Verrucomicr	[Spartobacteria]	[Chthoniobacterales]	[Chthoniobacteraceae]	Candidatus Xiphinematobacter
693574	11.3421854	4.11774701	1.09238334	3.76950735	0.00016357	0.00070627	Bacteria	SR1				
509913	214.98868	4.06961938	0.61743693	6.59114998	4.36E-11	7.84E-10	Bacteria	Proteobacteri	Gammaproteobacteria	Alteromonadales	Alteromonadaceae	Marinobacter
367995	18.7067807	4.04153959	1.33541472	3.02643031	0.0024746	0.00737086	Bacteria	Proteobacteri	Alphaproteobacteria	Sphingomonadales	Sphingomonadaceae	Kaistobacter
New.Referen	5.94324159	4.02535215	1.0999315	3.65963895	0.00025257	0.00098379	Bacteria	Firmicutes	Clostridia	Clostridiales	Veillonellaceae	
2061560	2.76993882	4.02525623	1.36602633	2.94669008	0.00321195	0.00912784	Bacteria	GN02	BD1-5			
724604	99.3265412	3.98014532	0.58013043	6.86077669	6.85E-12	1.36E-10	Bacteria	OP3	koll11	GIF10		
177975	3248.74099	3.9693343	0.55453011	7.15801408	8.19E-13	1.98E-11	Bacteria	Proteobacteri	Alphaproteobacteria	Sphingomonadales	Sphingomonadaceae	Novosphingobium

137097	2.10396667	3.96714398	1.35620758	2.92517462	0.00344263	0.00958772	Bacteria	Proteobacteri	Betaproteobacteria	Neisseriales	Neisseriaceae	Deefgea
149505	25.3765926	3.95041041	0.79451985	4.97207262	6.62E-07	4.50E-06	Bacteria	Proteobacteri	Alphaproteobacteria	Rhodobacterales	Hyphomonadaceae	Oceanicaulis
787709	17.6310131	3.94888179	1.01729701	3.88173932	0.00010371	0.00047351	Bacteria	Actinobacteri	Actinobacteria	Actinomycetales	Actinomycetaceae	Actinomyces
37406	189.830678	3.94319771	0.7927891	4.9738294	6.56E-07	4.50E-06	Bacteria	Proteobacteri	Alphaproteobacteria	Rhodospirillales	Rhodospirillaceae	Telmatospirillum
565691	187.018418	3.91531171	0.54740631	7.15247821	8.52E-13	1.98E-11	Bacteria	Bacteroidetes	Bacteroidia	Bacteroidales		
579608	49.8163253	3.88807339	0.76173791	5.10421409	3.32E-07	2.47E-06	Bacteria	Firmicutes	Bacilli	Lactobacillales	Streptococcaceae	Streptococcus
269031	5.2415	3.86411382	1.03028371	3.75053374	0.00017646	0.0007446	Bacteria	Firmicutes	Clostridia	OPB54		
578551	9.53383867	3.7935337	0.79033687	4.79989463	1.59E-06	1.00E-05	Bacteria	Proteobacteri	Alphaproteobacteria	Sphingomonadales		
984831	36.887136	3.68046445	0.73250004	5.02452461	5.05E-07	3.65E-06	Bacteria	Actinobacteri	Actinobacteria	Actinomycetales	Corynebacteriaceae	Corynebacterium
996116	9.66829836	3.56944067	1.20074269	2.97269406	0.00295199	0.00849675	Bacteria	Actinobacteri	Actinobacteria	Actinomycetales	Nocardioidaceae	
688528	18.1169697	3.5572629	0.87556562	4.06281702	4.85E-05	0.00023483	Bacteria	Bacteroidetes	Bacteroidia	Bacteroidales	Porphyromonadaceae	
854050	18.6157845	3.53176471	0.64337297	5.48945154	4.03E-08	3.74E-07	Bacteria	Firmicutes	Bacilli	Bacillales	Planococcaceae	Solibacillus
1083508	1426.50992	3.48982524	0.69522885	5.01967842	5.18E-07	3.66E-06	Bacteria	Proteobacteri	Gammaproteobacteria	Xanthomonadales	Xanthomonadaceae	Stenotrophomonas
4300564	911.628123	3.47573454	0.67109643	5.17918792	2.23E-07	1.75E-06	Bacteria	Proteobacteri	Alphaproteobacteria	Caulobacterales	Caulobacteraceae	Asticcacaulis
990864	4.34709823	3.46600775	1.18913289	2.91473543	0.0035599	0.00981617	Bacteria	Proteobacteri	Gammaproteobacteria	Pseudomonadales	Moraxellaceae	Enhydrobacter
New.Referen	36.6818479	3.44389974	0.85557221	4.02525902	5.69E-05	0.00027094	Bacteria	Cyanobacteri	ML635J-21			
1105280	177.080276	3.42488498	0.45946576	7.45405928	9.05E-14	2.52E-12	Bacteria	Proteobacteri	Betaproteobacteria	Burkholderiales	Burkholderiaceae	Burkholderia
909170	69.9441111	3.34171408	1.02381231	3.26399094	0.00109855	0.00366402	Bacteria	Verrucomicro	Verrucomicrobiae	Verrucomicrobiales	Verrucomicrobiaceae	Luteolibacter
621037	14.6284028	3.3156871	0.71559849	4.6334462	3.60E-06	2.17E-05	Bacteria	Planctomycet	Phycisphaerae	MSBL9		
New.Referen	32.3314378	3.30468053	0.60113015	5.49744603	3.85E-08	3.64E-07	Bacteria	Planctomycet	Phycisphaerae	AKAU3564		
829814	39.9776008	3.29078099	0.61782038	5.32643642	1.00E-07	8.45E-07	Bacteria	Proteobacteri	Alphaproteobacteria	Rhodobacterales	Rhodobacteraceae	Loktanella
842848	38.7215306	3.28363566	0.60980089	5.38476693	7.25E-08	6.41E-07	Bacteria	Armatimonac	Armatimonadia	FW68		
798326	258.140834	3.21089666	0.61460406	5.2243336	1.75E-07	1.45E-06	Bacteria	OP3	BD4-9			
1021333	6.61119923	3.20935257	0.78800562	4.07275339	4.65E-05	0.00022901	Bacteria	Armatimonac	SJA-176	RB046		
349901	34.7805985	3.19586327	0.52005704	6.14521685	7.99E-10	1.08E-08	Bacteria	Proteobacteri	Gammaproteobacteria	Legionellales	Coxiellaceae	Rickettsiella
267354	47.318998	3.17536568	1.08108425	2.93720464	0.00331186	0.00931668	Bacteria	Proteobacteri	Alphaproteobacteria	Rhodospirillales	Acetobacteraceae	Acidocella
614357	88.4679599	3.107975	0.61533336	5.0508801	4.40E-07	3.22E-06	Bacteria	Proteobacteri	Alphaproteobacteria	Rhizobiales	Methylocystaceae	Methylopila
816208	14.9673217	3.07832854	0.92373271	3.33248839	0.00086073	0.00292334	Bacteria	Acidobacteria				
845780	501.775143	2.97719201	0.73841171	4.03188621	5.53E-05	0.00026568	Bacteria	Bacteroidetes	Sphingobacteriia	Sphingobacteriales	Sphingobacteriaceae	
1671560	4.72100279	2.93596913	0.93047819	3.15533362	0.00160315	0.00501659	Bacteria	Chloroflexi	Dehalococcoidetes	Dehalococcoidales		
590601	887.090248	2.81037076	0.65670976	4.27947159	1.87E-05	9.84E-05	Bacteria	Proteobacteri	Betaproteobacteria	Burkholderiales	Oxalobacteraceae	
141636	24.5498188	2.79880963	0.80398375	3.48117684	0.00049922	0.0018662	Bacteria	Proteobacteri	Deltaproteobacteria	Bdellovibrionales	Bacteriovoracaceae	
New.Referen	9.63501131	2.73595369	0.93864302	2.91479684	0.0035592	0.00981617	Bacteria	Chloroflexi	Thermomicrobia	Ellin6537		
137070	26.8666829	2.71235933	0.58755528	4.61634745	3.91E-06	2.30E-05	Bacteria	OP3	koll11	GIF10	kpj58rc	
New.Referen	38.929165	2.68910639	0.55194977	4.8720129	1.10E-06	7.15E-06	Bacteria	Proteobacteri	Alphaproteobacteria	Rickettsiales	Rickettsiaceae	
139580	4.13188398	2.64887383	0.7920924	3.34414751	0.00082536	0.00283781	Archaea	Euryarchaeot	Methanomicrobia	Methanocellales	Methanocellaceae	Methanocella
646549	420.481249	2.63325225	0.6122544	4.30091189	1.70E-05	9.20E-05	Bacteria	Proteobacteri	Gammaproteobacteria	Pseudomonadales	Pseudomonadaceae	Pseudomonas
944197	22.3975427	2.61362797	0.52939732	4.93698759	7.93E-07	5.26E-06	Bacteria	Proteobacteri	Betaproteobacteria	Burkholderiales	Oxalobacteraceae	Herminiimonas
1024520	4.29999375	2.56716055	0.87384864	2.9377634	0.00330589	0.00931668	Bacteria	Proteobacteri	Betaproteobacteria	Burkholderiales	Comamonadaceae	Comamonas
New.Referen	16.0676119	2.45130205	0.57093824	4.29346271	1.76E-05	9.42E-05	Bacteria	Spirochaetes	Spirochaetes	Spirochaetales	Spirochaetaceae	Spirochaeta
1084865	52.1817934	2.37248521	0.64002588	3.70685825	0.00020985	0.00084699	Bacteria	Firmicutes	Bacilli	Bacillales	Staphylococcaceae	Staphylococcus
New.Referen	100.197505	2.34389992	0.62868117	3.72828076	0.00019279	0.00079736	Archaea	[Parvarchaeo	[Parvarchaea]	YLA114		
New.Referen	296.378924	2.31028318	0.6364231	3.63010582	0.00028331	0.00108828	Bacteria	Cyanobacteri	Chloroplast	Stramenopiles		
969805	21.6013046	2.30821679	0.71388949	3.23329707	0.0012237	0.00402481	Bacteria	Proteobacteri	Alphaproteobacteria	Rhizobiales	Rhizobiaceae	Agrobacterium
New.Referen	10.7791121	2.29463201	0.74810217	3.06727092	0.00216023	0.00661125	Bacteria	Elusimicrobia	Endomicrobia			
613703	74.6751836	2.23135972	0.74565173	2.99249588	0.00276706	0.00811187	Bacteria	OD1				
New.Referen	270.197685	2.21505513	0.73565344	3.01100359	0.00260386	0.00771462	Archaea	[Parvarchaeo	[Parvarchaea]	WCHD3-30		
87245	13.5744738	2.14854458	0.63202008	3.39948784	0.00067512	0.00241053	Bacteria	Proteobacteri	Deltaproteobacteria	Desulfovibrionales	Desulfovibrionaceae	Desulfovibrio
758197	300.778693	2.03311133	0.50910976	3.99346368	6.51E-05	0.00030478	Bacteria	Proteobacteri	Deltaproteobacteria	MIZ46		
New.Referen	41.4611318	2.00874088	0.48509785	4.14089833	3.46E-05	0.00017518	Bacteria	AD3	JG37-AG-4			
553643	14.1492914	2.00255435	0.58984804	3.39503436	0.0006862	0.00243448	Bacteria	Acidobacteria	Holophagae	Holophagales	Holophagaceae	Geothrix
812105	161.768559	-2.0125343	0.42413437	-4.7450393	2.08E-06	1.29E-05	Bacteria	WS3	PRR-12	Sediment-1	PRR-10	
4327003	387.174554	-2.0823971	0.6373667	-3.2671885	0.00108621	0.0036447	Bacteria	WPS-2				
791738	78.2616998	-2.0901791	0.68784971	-3.0387148	0.0023759	0.00711492	Bacteria	Proteobacteri	Betaproteobacteria	Burkholderiales	Comamonadaceae	Polaromonas
709657	1326.44012	-2.1207208	0.3009531	-7.0466822	1.83E-12	3.93E-11	Bacteria	Proteobacteri	Alphaproteobacteria	Rhodospirillales	Rhodospirillaceae	
566568	51.747105	-2.1270107	0.54962867	-3.8699049	0.00010888	0.00049305	Bacteria	Proteobacteri	Betaproteobacteria	Burkholderiales	Alcaligenaceae	
78839	1050.80877	-2.1394572	0.38311881	-5.5843179	2.35E-08	2.38E-07	Bacteria	Acidobacteria	DA052	Ellin6513		
222768	1066.75263	-2.1509072	0.4717001	-4.5599041	5.12E-06	2.97E-05	Bacteria	Proteobacteri	Alphaproteobacteria	Ellin329		
538428	153.694772	-2.1512497	0.6751748	-3.1862114	0.00144149	0.00466809	Bacteria	Acidobacteria	Acidobacteria-6	iii1-15	RB40	
895220	178.442321	-2.1707454	0.73041676	-2.9719271	0.00295937	0.00849675	Bacteria	Proteobacteri	Betaproteobacteria	Burkholderiales	Comamonadaceae	Leptothrix
837424	376.144954	-2.2890249	0.76770206	-2.9816578	0.00286692	0.00831706	Bacteria	Proteobacteri	Alphaproteobacteria	Rhodobacterales	Hyphomonadaceae	
806094	371.390917	-2.3614241	0.65773137	-3.5902561	0.00033035	0.00126032	Bacteria	Proteobacteri	Betaproteobacteria	Ellin6067		

261386	4.03675537	-2.3647155	0.746138	-3.1692737	0.0015282	0.00483642	Bacteria	NC10	24-Dec	JH-WHS47		
New.Referen	18.9922429	-2.4318142	0.66616661	-3.6504595	0.00026177	0.00101255	Bacteria	Chlorobi	BSV26	PK329		
532139	270.280667	-2.4345688	0.57363933	-4.2440759	2.19E-05	0.0001132	Bacteria	Proteobacteri	Betaproteobacteria	MND1		
New.Referen	951.527026	-2.4417882	0.66595497	-3.6665965	0.0002458	0.00096667	Bacteria	Bacteroidetes	[Saprospirae]	[Saprospirales]		
808692	19.9564007	-2.5394015	0.85596116	-2.9667251	0.0030099	0.00859751	Bacteria	Acidobacteri	TM1			
1053775	51.6637205	-2.5924278	0.67581497	-3.8360023	0.00012505	0.00055724	Bacteria	Proteobacteri	Alphaproteobacteria	Rhizobiales	Hyphomicrobiaceae	Devosia
New.Referen	13.8091642	-2.684209	0.57954696	-4.6315643	3.63E-06	2.17E-05	Bacteria	Proteobacteri	Alphaproteobacteria	Rhizobiales	Bradyrhizobiaceae	
29360	5323.57993	-2.7657071	0.6421431	-4.3069949	1.65E-05	9.04E-05	Bacteria	Proteobacteri	Alphaproteobacteria	Rhodospirillales	Acetobacteraceae	
222087	919.97781	-2.7696109	0.37054565	-7.4744121	7.75E-14	2.27E-12	Bacteria	Acidobacteri	Solibacteres	Solibacterales	Solibacteraceae	Candidatus Solibacter
New.Referen	499.498918	-2.8173503	0.51659138	-5.4537307	4.93E-08	4.50E-07	Bacteria	Verrucomicr	[Pedosphaerae]	[Pedosphaerales]		
New.Referen	41.2000757	-2.8408867	0.76553633	-3.7109757	0.00020646	0.00083941	Bacteria	Proteobacteri	Gammaproteobacteria	Xanthomonadales	Sinobacteraceae	Steroidobacter
New.Referen	1448.13737	-2.84105	0.4526822	-6.2760365	3.47E-10	4.84E-09	Bacteria	Planctomycet	Planctomycetia	Gemmatales	Isosphaeraceae	
687206	469.990828	-2.8570248	0.98201419	-2.9093518	0.00362179	0.00993762	Bacteria	Acidobacteri	[Chloracidobacteria]	RB41	Ellin6075	
New.Referen	86.9723168	-2.9281502	0.66209712	-4.4225388	9.75E-06	5.43E-05	Bacteria	Chloroflexi	Anaerolineae	SBR1031	oc28	
New.Referen	751.259166	-2.9478999	0.37348604	-7.8929321	2.95E-15	1.17E-13	Bacteria	Acidobacteri	Solibacteres	Solibacterales		
New.Referen	119.920417	-2.9526044	0.86490512	-3.4137899	0.00064066	0.00230224	Bacteria	Chloroflexi	TK17			
330902	100.45681	-3.0877847	0.65370404	-4.7235209	2.32E-06	1.42E-05	Bacteria	Proteobacteri	Betaproteobacteria	Burkholderiales		
3701020	628.697358	-3.0961202	0.84259585	-3.6745021	0.00023831	0.00094815	Bacteria	Verrucomicr	[Pedosphaerae]	[Pedosphaerales]	R4-41B	
314567	1410.05238	-3.0988272	0.33842536	-9.1566046	5.36E-20	2.98E-18	Bacteria	Planctomycet	Planctomycetia	Gemmatales	Gemmataceae	Gemmata
557467	9.06360293	-3.1073365	0.91619103	-3.3915814	0.00069491	0.00244976	Bacteria	Gemmatimon	Gemmatimonadetes	Gemmatimonadales	Ellin5301	
3900307	1925.7762	-3.1194057	0.60094137	-5.1908653	2.09E-07	1.67E-06	Bacteria	Armatimonac	[Fimbriimonadia]	[Fimbriimonadales]	[Fimbriimonadaceae]	Fimbriimonas
4418260	7.90382212	-3.1651914	1.0245865	-3.0892378	0.00200671	0.00617534	Bacteria	Proteobacteri	Betaproteobacteria	Burkholderiales	Comamonadaceae	Aquabacterium
515709	2077.57003	-3.2094402	0.34364497	-9.3394069	9.69E-21	7.57E-19	Bacteria	Proteobacteri	Alphaproteobacteria	Rhizobiales	Hyphomicrobiaceae	Rhodoplanes
1049387	4.0492769	-3.3143678	1.07283945	-3.0893418	0.00200601	0.00617534	Bacteria	Proteobacteri	Gammaproteobacteria	Xanthomonadales	Xanthomonadaceae	Pseudoxanthomonas
New.Referen	323.543352	-3.3308233	0.73889122	-4.5078669	6.55E-06	3.68E-05	Bacteria	Armatimonac	Chthonomonadetes	SJA-22		
New.Referen	46.4321694	-3.3744634	0.97759323	-3.4518073	0.00055685	0.00205406	Bacteria	Chloroflexi	Anaerolineae	SBR1031	A4b	
1039699	798.861441	-3.5004482	0.59317339	-5.9012226	3.61E-09	4.47E-08	Bacteria	Armatimonac	Chthonomonadetes	Chthonomonadales	Chthonomonadaceae	
810167	201.122212	-3.5117392	0.46175356	-7.605224	2.84E-14	9.32E-13	Bacteria	Proteobacteri	Betaproteobacteria	Burkholderiales	Comamonadaceae	Methylibium
New.Referen	363.806609	-3.5337455	0.6525087	-5.4156297	6.11E-08	5.49E-07	Bacteria	Planctomycet	Phycisphaerae	Phycisphaerales		
New.Referen	329.448147	-3.5999462	0.54983002	-6.5473802	5.86E-11	9.59E-10	Bacteria	Gemmatimon	Gemmatimonadetes	KD8-87		
525955	21.246469	-3.6071944	1.07348781	-3.3602565	0.0007787	0.00271085	Bacteria	Bacteroidetes	Flavobacteriia	Flavobacteriales	Cryomorphaceae	
979536	15.8808	-3.7206881	1.11089218	-3.3492792	0.00081022	0.00280306	Bacteria	Acidobacteri	Solibacteres	Solibacterales	Solibacteraceae	
516569	8005.54032	-3.7727364	0.73738807	-5.1163513	3.12E-07	2.38E-06	Bacteria	Verrucomicr	Verrucomicrobiae	Verrucomicrobiales	Verrucomicrobiaceae	Prosthecobacter
210532	86.466691	-3.7837769	0.88300353	-4.2851209	1.83E-05	9.69E-05	Bacteria	Chloroflexi	Anaerolineae	H39		
811449	293.212112	-3.8650965	0.67951992	-5.6879811	1.29E-08	1.41E-07	Bacteria	Proteobacteri	Alphaproteobacteria	Caulobacterales	Caulobacteraceae	Caulobacter
4614	52.1115914	-3.8961536	0.91888652	-4.2400813	2.23E-05	0.00011418	Bacteria	Proteobacteri	Alphaproteobacteria	Rhizobiales	Hyphomicrobiaceae	Pedomicrobium
328951	40.5726735	-3.9410391	0.75696897	-5.2063417	1.93E-07	1.58E-06	Bacteria	Nitrospirae	Nitrospira	Nitrospirales	Nitrospiraceae	Nitrospira
New.Referen	1.83291101	-4.0945273	1.11691341	-3.6659308	0.00024644	0.00096667	Bacteria	Proteobacteri	Alphaproteobacteria	Rhizobiales	Bradyrhizobiaceae	Afipia
2572567	1.91314352	-4.1403171	1.09175415	-3.792353	0.00014923	0.00065968	Bacteria	Proteobacteri	Alphaproteobacteria	Rickettsiales	mitochondria	Pleurozia
580625	3092.33013	-4.141627	0.45663073	-9.0699699	1.19E-19	6.03E-18	Bacteria	Proteobacteri	Alphaproteobacteria	Rhizobiales	Bradyrhizobiaceae	Bradyrhizobium
4347035	36.7450181	-4.142397	1.05179308	-3.9384144	8.20E-05	0.00037757	Bacteria	Acidobacteri	EC1113			
New.Referen	511.083157	-4.1793426	0.87503047	-4.7762253	1.79E-06	1.12E-05	Bacteria	Bacteroidetes	[Saprospirae]	[Saprospirales]	Saprospiraceae	
567840	79.3682596	-4.3851942	0.68923486	-6.3624092	1.99E-10	2.84E-09	Bacteria	Proteobacteri	Alphaproteobacteria	Rhizobiales	Bradyrhizobiaceae	Bosea
814282	11.5358747	-4.5006942	1.20433376	-3.7370822	0.00018617	0.00077967	Bacteria	Proteobacteri	Alphaproteobacteria	Sphingomonadales	Erythrobacteraceae	
141815	38.9899259	-4.6394294	0.84246415	-5.5069754	3.65E-08	3.51E-07	Bacteria	Armatimonac	Armatimonadia	Armatimonadales	Armatimonadaceae	Armatimonas
New.Referen	3.03688536	-4.7619385	1.48076919	-3.2158546	0.00130057	0.00423635	Bacteria	Proteobacteri	Gammaproteobacteria	Xanthomonadales	Sinobacteraceae	Alkanibacter
846667	35.1656619	-4.9420603	1.07075515	-4.6154905	3.92E-06	2.30E-05	Bacteria	Acidobacteri	Acidobacteriia	Acidobacteriales	Acidobacteriaceae	Acidicapsa
552017	9.99403307	-5.1658596	1.27029438	-4.0666634	4.77E-05	0.00023302	Bacteria	NKB19	TSBW08			
4017244	84.769986	-5.28683	1.27973908	-4.1311781	3.61E-05	0.00018111	Bacteria	Spirochaetes	[Leptospirae]	[Leptospirales]	Leptospiraceae	Turneriella
809945	37.6296136	-5.5831247	0.99453477	-5.6138054	1.98E-08	2.12E-07	Bacteria	Proteobacteri	Betaproteobacteria	Methylophilales		
583489	933.872618	-5.7050201	0.38552547	-14.798037	1.51E-49	4.20E-47	Bacteria	Proteobacteri	Betaproteobacteria			
143418	143.375367	-6.1408307	1.22931283	-4.9953361	5.87E-07	4.09E-06	Bacteria	Verrucomicr	[Pedosphaerae]	[Pedosphaerales]	Ellin517	
New.Referen	7.962738	-6.1655609	1.65788225	-3.7189377	0.00020006	0.00081937	Bacteria	Bacteroidetes	Flavobacteriia	Flavobacteriales	Flavobacteriaceae	Gilvibacter
831179	210.47917	-6.1711692	0.91945098	-6.7117979	1.92E-11	3.57E-10	Bacteria	Proteobacteri	Betaproteobacteria	Burkholderiales	Comamonadaceae	Rubrivivax
100640	266.11428	-6.3505997	0.67738142	-9.3752198	6.90E-21	6.41E-19	Bacteria	Proteobacteri	Betaproteobacteria	IS-44		
556648	225.664617	-7.7899417	1.02567475	-7.5949434	3.08E-14	9.53E-13	Bacteria	Acidobacteri	[Chloracidobacteria]	PK29		
1501738	522.507808	-8.0454677	1.15099066	-6.9900374	2.75E-12	5.67E-11	Bacteria	Proteobacteri	Betaproteobacteria	Rhodocyclales	Rhodocyclaceae	
104155	1377.05318	-9.2483463	0.62074688	-14.89874	3.36E-50	1.87E-47	Bacteria	Proteobacteri	Gammaproteobacteria	Xanthomonadales	Sinobacteraceae	Nevskia
New.Referen	11695.7129	-9.3164542	0.99885046	-9.3271761	1.09E-20	7.57E-19	Bacteria	Bacteroidetes	Flavobacteriia	Flavobacteriales	Flavobacteriaceae	Zhouia

Table S13. Putative DBNPA brominated degradation products detected by nano-HPLC-HRMS. The elemental formula was predicted using the formula predictor in Xcalibur Software (Thermo Fisher Scientific) and the putative structure was confirmed using ChemDraw (PerkinElmer).

m/z	Predicted Elemental Formula	Putative Structure
85.0396	$C_3H_5O_2N_2$	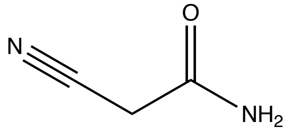<br>cyanoacetamide<br>CAM
119.9444	C_2H_3NBr	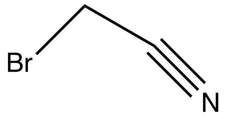<br>*2-bromoacetonitrile*
134.8526	C_2O_2Br	
137.9544	C_2H_5ONBr	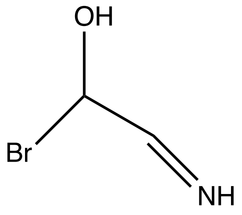<br>*1-bromo-2-iminoethan-1-ol*<br>*(119 + H_2O)*
146.9077	C_3O_2Br	
163.9335	$C_3H_3O_2NBr$	*119 + CO_2*
164.9476	$C_3H_3BrN_2O$	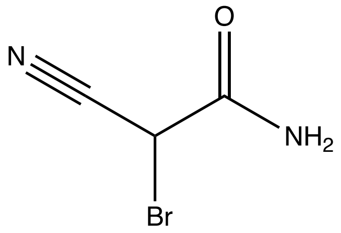<br>monobromnitrilopropionamide

		MBNPA
172.8413	$CHBr_2$	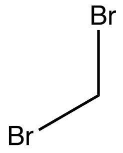<br><br>*Dibromomethane*<br>*DBAN - CN*
182.9586	$C_3H_6O_2N_2Br$	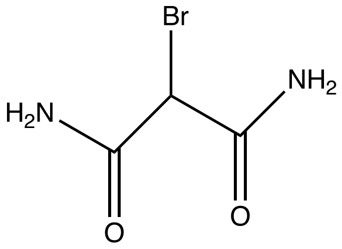<br><br>*2-bromomalonamide*<br>*MBNPA + H_2O*
199.8528	C_2HBr_2N	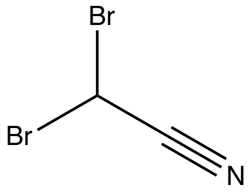<br><br>dibromoacetonitrile<br>DBAN
217.8628	$C_2H_3Br_2NO$	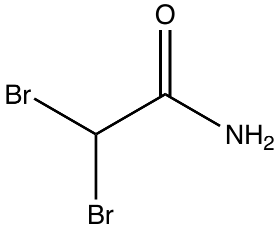<br><br>dibromoacetamide<br>DBAM

242.8587	$C_3H_2Br_2N_2O$	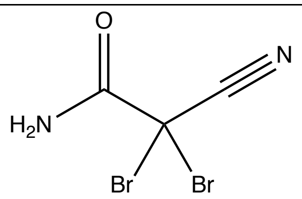<br>DBNPA
244.9106	$C_4H_9N_2Br_2$	
260.8682	$C_4H_4O_2NBr_2$	*DBNPA + H_2O*
305.9254	$C_3H_{10}ON_6Br_2$	
306.9616	$C_8H_4O5N_3Br$	
314.0824	$C_{13}H_{19}N_4Br$	
318.8959	$C_9H_5N_3Br_2$	

Table S14. Total Organic Carbon (TOC) concentration in source water prior to DBNPA addition.

Site	TOC (mg/L)	Standard Error
AB	8.57	2.14
LL	8.04	2.60
NH	6.84	1.62
EE	1.70	.62
WE	4.83	1.30
DR	5.73	2.06

Table S15. Geological coordinates and watershed physiochemical parameters.

	**Stream**	**Latitude**	**Longitude**	**pH**	**Temperature (°C)**	**Conductivity (µS)**	**Total Dissolved**

							**Solids (ppm)**
**HF+**	Alex Branch (AB)	41.168	-78.453	4.98	15.7	30.5	21.8
	Little Laurel (LL)	41.15	-78.474	4.68	20.6	34.8	25
	UNT Naval Hollow (NH)_	41.59339,	-78.5141	5.13	14.1	22.3	15.5
**HF-**	UNT East Elk (EE)	41.61288	-78.4214	7.3	13.2	43.4	30.8
	UNT West Elk (WE)	41.61185	-78.4222	6.48	13.6	34	24.1
	Dixon Run (DR)	41.154	-78.39	5.71	11.7	23.8	16.9

Supplemental References

Blanchard, F.A., Gonsior, S.J., and Hopkins, D.L. (1987) 2,2-Dibromo-3-nitrilopropionamide (DBNPA) chemical degradation in natural waters: Experimental evaluation and modeling of competitive pathways. *Water Research* **21**: 801-807.

Exner, J.H., Burk, G.A., and Kyriacou, D. (1973) Rates and Products of Decomposition of 2,2-Dibromo-3-Nitrilopropionamide. *Journal of Agricultural and Food Chemistry* **21**: 838-842.
